# Proxy-analysis of the genetics of cognitive decline in Parkinson’s disease through polygenic scores

**DOI:** 10.1038/s41531-023-00619-5

**Published:** 2024-01-04

**Authors:** Johann Faouzi, Manuela Tan, Fanny Casse, Suzanne Lesage, Christelle Tesson, Alexis Brice, Graziella Mangone, Louise-Laure Mariani, Hirotaka Iwaki, Olivier Colliot, Lasse Pihlstrøm, Jean-Christophe Corvol

**Affiliations:** 1 Sorbonne Université, Institut du Cerveau–Paris Brain Institute-ICM, CNRS, Inria, Inserm, AP-HP, Hôpital de la Pitié Salpêtrière, F-75013 Paris, France; 2 Univ Rennes, Ensai, CNRS, CREST–UMR 9194, F-35000 Rennes, France; 3 Department of Neurology, Oslo University Hospital, Oslo, Norway; 4 Sorbonne Université, Institut du Cerveau–Paris Brain Institute-ICM, CNRS, Inserm, AP-HP, Hôpital de la Pitié Salpêtrière, Paris, France; 5 Sorbonne Université, Institut du Cerveau–Paris Brain Institute-ICM, CNRS, Inserm, AP-HP, Hôpital de la Pitié Salpêtrière, DMU Neurosciences, Département de Génétique, F-75013 Paris, France; 6 Sorbonne Université, Institut du Cerveau–Paris Brain Institute-ICM, CNRS, Inserm, AP-HP, Hôpital de la Pitié Salpêtrière, DMU Neurosciences, Département de Neurologie, F-75013 Paris, France; 7 Department of Neurology, Movement Disorder Division, Rush University Medical Center, 1725 W. Harrison Street, Chicago, IL 60612 USA; 8 Laboratory of Neurogenetics, National Institute on Aging, National Institutes of Health, Bethesda, MD USA; 9 Center for Alzheimer’s and Related Dementias (CARD), National Institute on Aging and National Institute of Neurological Disorders and Stroke, National Institutes of Health, Bethesda, MD USA; 10 Data Tecnica International LLC, Washington, DC USA

**Keywords:** Parkinson's disease, Genome-wide association studies, Dementia

## Abstract

Cognitive decline is common in Parkinson’s disease (PD) and its genetic risk factors are not well known to date, besides variants in the *GBA* and *APOE* genes. However, variation in complex traits is caused by numerous variants and is usually studied with genome-wide association studies (GWAS), requiring a large sample size, which is difficult to achieve for outcome measures in PD. Taking an alternative approach, we computed 100 polygenic scores (PGS) related to cognitive, dementia, stroke, and brain anatomical phenotypes and investigated their association with cognitive decline in six longitudinal cohorts. The analysis was adjusted for age, sex, genetic ancestry, follow-up duration, *GBA* and *APOE* status. Then, we meta-analyzed five of these cohorts, comprising a total of 1702 PD participants with 6156 visits, using the Montreal Cognitive Assessment as a cognitive outcome measure. After correction for multiple comparisons, we found four PGS significantly associated with cognitive decline: intelligence (*p* = 5.26e–13), cognitive performance (*p* = 1.46e–12), educational attainment (*p* = 8.52e–10), and reasoning (*p* = 3.58e–5). Survival analyses highlighted an offset of several years between the first and last quartiles of PGS, with significant differences for the PGS of cognitive performance (5 years) and educational attainment (7 years). In conclusion, we found four PGS associated with cognitive decline in PD, all associated with general cognitive phenotypes. This study highlights the common genetic factors between cognitive decline in PD and the general population, and the importance of the participant’s cognitive reserve for cognitive outcome in PD.

## Introduction

Although Parkinson’s disease (PD) is clinically defined by its cardinal motor symptoms, numerous non-motor symptoms frequently occur during the course of the disease. Among them, cognitive decline is common, with the point prevalence of PD dementia being approximately 30% and the cumulative prevalence being at least 75% for PD participants surviving more than ten years^1^. Cognitive decline strongly impacts the quality of life and life expectancy of the participants^2,3^.

The genetic risk factors of cognitive decline in PD are still mostly unknown. Genetic risk factors for cognitive decline in PD have been investigated in specific genes related to genetic forms of PD or cognitive disorders^4,5^. Mutations in the glucocerebrosidase (*GBA*) gene, responsible for the autosomal recessive Gaucher’s disease, have been demonstrated to be a strong risk factor for PD^6^, but have also been associated with greater cognitive decline in PD^7–11^. Polymorphisms of the apolipoprotein E (*APOE*) gene associated with Alzheimer’s disease^12^ have also been shown to be associated with cognitive decline in PD^11,13–17^. Investigations in other genes, including microtubule-associated protein tau (*MAPT*)^15–17^, leucine-rich repeat serine/threonine-protein kinase 2 (*LRRK2*)^18–20^, α-synuclein (*SNCA*)^16,21^, catechol-O-methyltransferase (*COMT*)^14,15,17^, and brain-derived neurotrophic factor (*BNDF*)^22,23^, have led to conflicting results. Genome-wide investigation of cognitive decline in PD has been limited. No genome-wide significant association with cognitive decline could be reported in two different GWAS^24,25^. Other GWAS confirmed the association with mutations in *GBA* and *APOE* in PD^11,26^ and Lewy body dementia (LBD)^27^, and reported genome-wide significant associations with Apolipoprotein C1 (*APOC1)*, translocase of outer mitochondrial membrane 40 (*TOMM40*), the regulating synaptic membrane exocytosis 2 (*RIMS2*) genes, as well as suggestive associations in transmembrane protein 108 (*TMEM108*) and WW domain-containing oxidoreductase (*WWOX*) genes in PD, but with limited effect sizes^11,26^. Another study reported significant associations between PD dementia and variants in the mitochondrial E3 ubiquitin protein ligase 1 (*MUL1*), zinc fingers and homeoboxes 2 (*ZHX2*) and endoplasmic reticulum resident protein 29 (*ERP29*) genes^28^. Finally, a recent study showed an association between cognitive decline in PD and mitochondrial haplogroups^29^. All these studies suffered from limited power due to the limited number of PD participants included in the analyses and highlighted the limited effect sizes of individual variants.

Variation in complex phenotypes is caused by numerous genetic variants, each one usually carrying only a small relative risk. However, the combination of the risk of numerous low-risk variants can explain a substantial proportion of the genetic variance. Polygenic scores (PGS) additively combine the weighted risk of every trait-associated genetic variant into a single score. PGS is computed by estimating the joint effects of individual genotypes from the marginal effects obtained from summary statistics of large-scale genome-wide association studies (GWAS).

In this study, we performed a proxy-analysis of the genetics of cognitive decline in PD through PGS. We used clinical and genetic data from six longitudinal cohorts. We computed PGS from publicly available summary statistics for a broad range of phenotypes and investigated their associations with longitudinal cognitive scores. Our objective was to identify the genetic similarity between cognitive decline in PD and other phenotypes.

## Results

### Participants

**Fig. 1:**
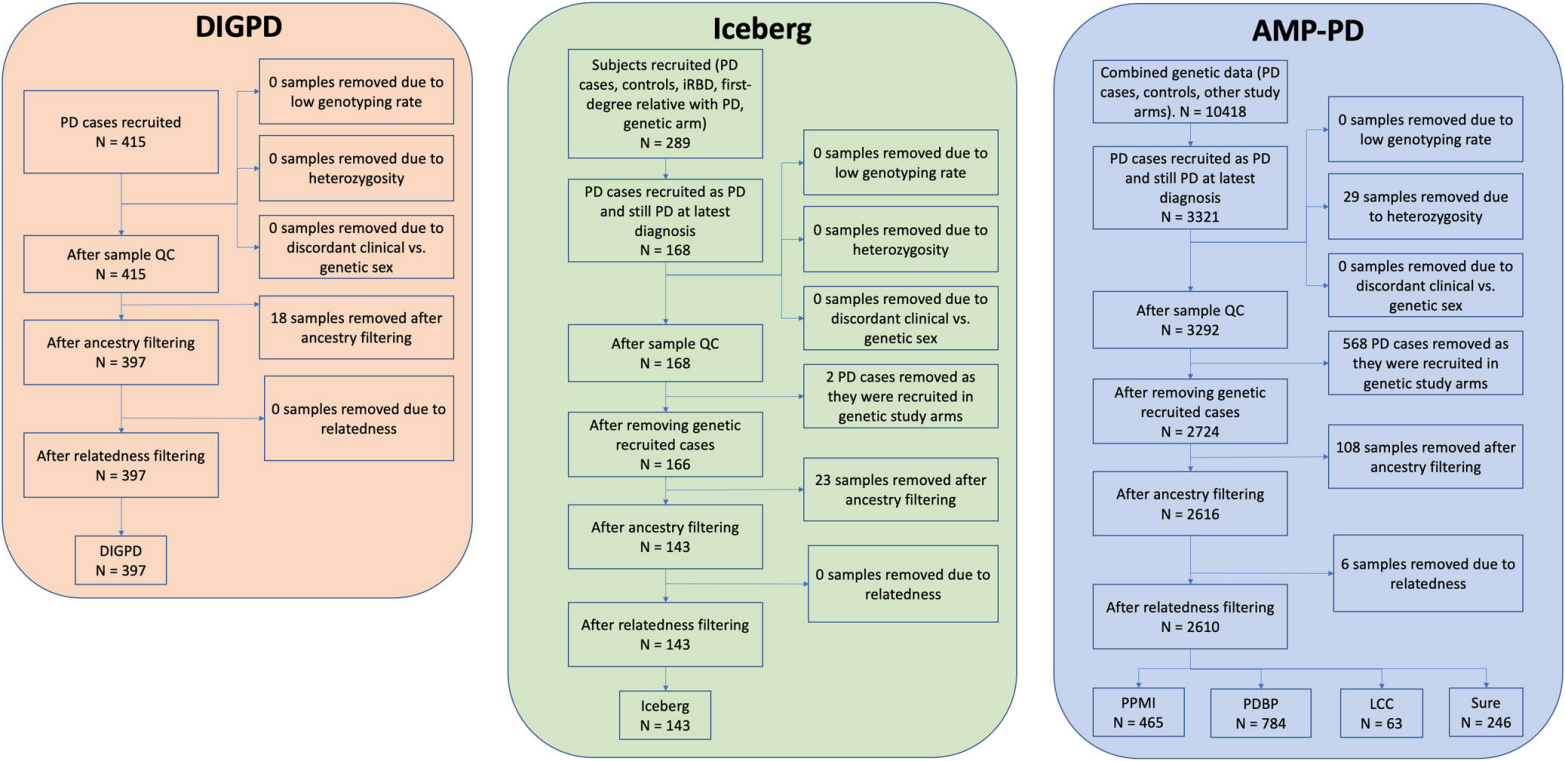
Flowchart. Flowchart indicating the initial number of participants, the number of participants at each step, and the final number of PD participants included in the analyses. iRBD idiopathic rapid eye movement sleep disorder, PD Parkinson’s disease.

In total, 2089 PD participants and 8141 visits were included in our analyses. The details on the inclusion and exclusion criteria of each cohort are provided in supplementary materials. A flowchart describing the number of participants at each step of the quality control is given in Fig. 1. Table 1 presents the characteristics of the participants in each cohort. There were differences across cohorts in terms of age, sex, length of follow-up, interval between visits, baseline MoCA scores, as well as baseline and lifetime cognitive decline that we adjusted for further analyses. The list of *GBA* mutations is described in Supplementary Table 1.

**Table 1:** Participants’ characteristics.

Characteristic	DIGPD	Iceberg	PPMI	PDBP	SURE-PD3	LCC	*p*-value
Number of participants	387	144	465	784	246	63	
Age at baseline (in years)	62.37 ± 9.63	62.94 ± 9.22	61.77 ± 9.71	64.49 ± 9.01	62.71 ± 9.50	68.03 ± 9.74	1.11e–08
Sex (F/M)	156/231 (40%)	52/92 (36%)	165/300 (35%)	283/501 (36%)	119/127 (48%)	22/41 (35%)	4.39e–15
Length of follow-up (in years)	5.13 ± 1.91	3.73 ± 1.36	5.23 ± 2.85	1.39 ± 1.60	2.04 ± 0.68	2.22 ± 1.92	6.97e–230
Interval between visits (in years)	1.09 ± 0.32	1.11 ± 0.27	1.06 ± 0.28	1.05 ± 0.24	0.79 ± 0.26	1.47 ± 0.70	8.28e–152
Baseline cognitive score	MMSE: 28.18 ± 1.90	MoCA: 27.53 ± 1.98	MoCA: 27.12 ± 2.32	MoCA: 25.37 ± 3.51	MoCA: 27.61 ± 1.88	MoCA: 25.41 ± 4.42	3.65e–38
Baseline cognitive decline (yes/no)	111/276 (29%)	11/133 (8%)	58/407 (12%)	266/518 (34%)	12/234 (5%)	20/43 (32%)	4.11e-–32
Lifetime cognitive decline (yes/no)	241/146 (62%)	35/109 (24%)	189/276 (41%)	350/434 (45%)	28/218 (11%)	30/33 (48%)	1.90e-–37
rs7412–number of T alleles (0/1/2)	337/49/1	126/18/0	392/68/5	673/106/5	221/23/2	51/12/0	
rs429358–number of C alleles (0/1/2)	289/92/6	115/25/4	345/109/11	587/182/15	171/68/7	51/12/0	
Number of severe GBA mutations (0/1)	387/0	144/0	464/1	781/3	245/1	63/0	
Number of mild GBA mutations (0/1/2)	380/7/0	141/3/0	420/44/1	716/65/3	230/16/0	58/5/0	
Number of undetermined GBA mutations (0/1/2+)	376/10/1	139/5/0	161/52/252	286/79/419	105/23/118	23/7/33	

For continuous variables, mean ± standard deviation is reported. For binary variables, the count for both categories and the proportion of the first category are reported. For count variables, the count is reported. Statistical differences were investigated using analysis of variance (ANOVA) F-tests for continuous variables and chi-squared tests for binary variables. Count variables were not tested for statistical differences due to very low frequencies for at least one category, making statistical tests inappropriate. Only MoCA scores were compared for the baseline cognitive scores. Cognitive decline was defined as a cognitive score below a given threshold (MMSE ≤ 27, MoCA ≤ 24). *MoCA* Montreal Cognitive Assessment. *MMSE* Mini-Mental State Examination.

### Genome-wide association studies

We identified 100 GWAS matching the defined criteria. The corresponding phenotypes consisted of height^30^, body mass index^31^, memory performance^32^, reasoning^32^, reaction time^33^, cognitive performance^34^, educational attainment^34^, intelligence^35^, Parkinson’s disease or first-degree relation to an individual with Parkinson’s disease^36^, Alzheimer’s disease^37^, Alzheimer’s disease or family history of Alzheimer’s disease^38^, Lewy body dementia^39^, five stroke subtypes^40^, major depressive disorder^41^, anxiety disorder^42^, sleeplessness or insomnia^43^, trouble falling asleep^43^, white matter hyperintensities^44^, intracranial volume^45^, subcortical volumes in seven brain regions^46^, and cortical surface areas and thicknesses in the whole brain and 34 brain regions^47^. Supplementary Table 2 provides detailed information about the phenotypes, the estimated SNP heritability, the number of participants, and the number of SNPs for each GWAS. Supplementary Table 3 provides the number of SNPs involved in each computed PGS.

### Association analyses

Partial correlation coefficients between the real phenotypes of height and body mass index and the corresponding PGS were coherent with the literature (*r* = 0.60 [0.57–0.63] for height, *r* = 0.26 [0.21–0.31] for body mass index), suggesting good PGS computation with regards to the current state of the art^30,31^.

**Fig. 2:**
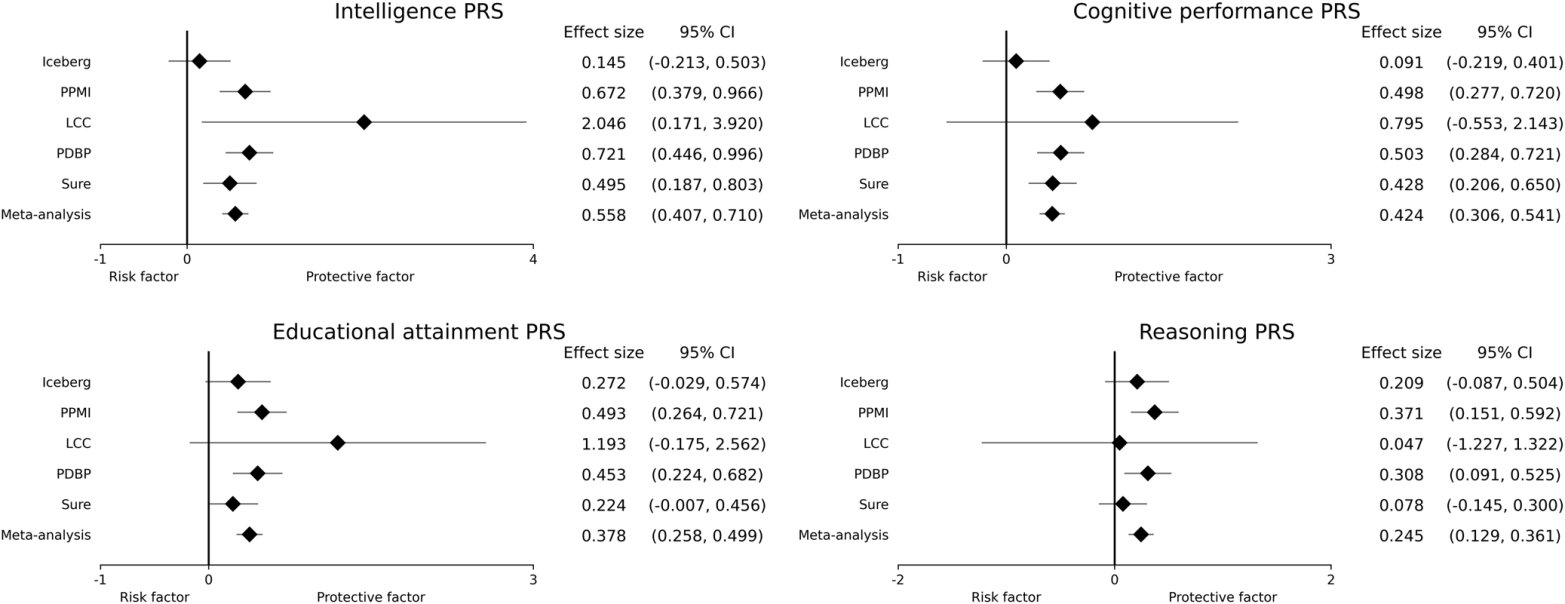
Forest plots. Forest plots for the four significant associations. Only the cohorts included in the meta-analysis are included.

Since DIGPD was the only cohort in which the MoCA was not used as the cognitive screening test, it was not included in the meta-analysis. The meta-analysis including the other five cohorts, for a total of 1702 PD patients with 6156 visits, revealed four significant associations, corresponding to the PGS of intelligence, cognitive performance, educational attainment, and reasoning (Table 2). All the associations were in the same direction as protective factors (the higher the PGS, the higher the cognitive scores, thus the less cognitive decline). The heterogeneity *p*-values were low for several PGS, suggesting heterogeneity in the results (Supplementary Table 5). Figure 2 illustrates the forest plots for the significant associations, confirming the heterogeneity in the effects with outlying values most found in LCC and Iceberg cohorts. Nonetheless, the directions were always identical in the five cohorts (Table 2).

**Table 2:** Statistical associations.

Cohort	Phenotype PGS	*p*-value	Direction	Effect size (95% CI)
Iceberg, PPMI, PDBP, SURE-PD3, LCC	Intelligence	5.26e−13	+	0.56 [0.41–0.71]
	Cognitive performance	1.46e−12	+	0.42 [0.31–0.54]
	Educational attainment	8.52e−10	+	0.38 [0.26–0.50]
	Reasoning	3.58e−05	+	0.25 [0.13–0.36]
DIGPD*	Reasoning	0.051		0.17 [−0.00–0.33]
	Intelligence	0.051		0.18 [−0.00–0.36]
	Educational attainment	0.085		0.15 [−0.02–0.33]
	Cognitive performance	0.12		0.13 [−0.04–0.30]
Iceberg	Educational attainment	0.08		0.27 [−0.03–0.57]
	Reasoning	0.17		0.21 [−0.09–0.50]
	Intelligence	0.43		0.15 [−0.21–0.50]
	Cognitive performance	0.56		0.09 [−0.22–0.40]
PPMI	Intelligence	7.05e−06	+	0.67 [0.38–0.97]
	Cognitive performance	1.04e−05	+	0.67 [0.38–0.97]
	Educational attainment	2.42e−05	+	0.50 [0.28–0.72]
	White matter hyperintensities	3.08e−04	−	−0.47 [−0.73–−0.21]
	Reasoning	9.77e−04		0.37 [0.15–0.59]
PDBP	Intelligence	2.71e−07	+	0.72 [0.45–1.00]
	Cognitive performance	6.40e−06	+	0.50 [0.28–0.72]
	Educational attainment	1.04e−04	+	0.45 [0.22–0.68]
	Reasoning	5.45e−03		0.31 [0.09–0.52]
SURE-PD3	Cognitive performance	1.56e−04	+	0.43 [0.21–0.65]
	Intelligence	1.65e−03		0.49 [0.19–0.80]
	Educational attainment	0.058		0.22 [-0.01–0.46]
	Reasoning	0.49		0.08 [-0.14–0.30]
LCC	Intelligence	0.033		2.05 [0.17–3.92]
	Educational attainment	0.087		1.19 [−0.18–2.56]
	Cognitive performance	0.25		0.79 [−0.55–2.14]
	Reasoning	0.94		0.05 [−1.23–1.32]

^*^DIGPD was not included in the meta-analysis due to the use of a different cognitive scale (Mini-Mental State Examination) compared to the other cohorts (Montreal Cognitive Assessment).

Associations with the meta-analysis (excluding DIGPD) and with independent analyses in all the cohorts after Bonferroni correction for multiple associations. Associations with the four significant PGS in the meta-analysis are also reported for the independent analyses, although not always significant. The usual 0.05 threshold was used to determine significance, which became 5e−04 after Bonferroni correction. The direction indicates the sign of the association: positive directions correspond to protective factors (the higher the PGS, the higher the cognitive score, the less cognitive decline), and negative directions correspond to risk factors (the higher the PGS, the lower the cognitive score, the more cognitive decline). Non-significant associations are denoted by the lack of direction. CI: confidence interval.

Several significant associations were also obtained in the independent analyses in each cohort (Table 2 and Supplementary Table 4): the PGS for intelligence, cognitive performance, educational attainment, reasoning, and white matter hyperintensities in PPMI, the PGS for intelligence, cognitive performance and educational attainment in PDBP, the PGS for cognitive performance and intelligence in SURE-PD3, and the PGS for intelligence in LCC. The directions for significant associations in different cohorts were always identical. In particular, associations with PGS of cognitive phenotypes (intelligence, cognitive performance, educational attainment, and reasoning) all had positive directions, meaning that these PGS were protective factors (the higher the PGS, the higher the cognitive scores, the less cognitive decline). The models’ residuals were normally distributed (Supplementary Figs. 1–4).

We also performed additional analyses and ablation experiments. We investigated the potential associations with interaction terms between the *APOE* and *GBA* covariates and each PGS, but did not obtain any significant association in the meta-analysis after correction for multiple comparisons (Supplementary Table 6). We also performed the same analyses without the *APOE* and *GBA* covariates and obtained the same four significant PGS in the meta-analysis (Supplementary Table 7). We finally investigated the cumulative predictive power of the model with four significant PGS compared to the models with each single PGS. The model including the four significant PGS was significantly better than three models including a single PGS, for the PGS of reasoning (*p* = 3.97e−8), educational attainment (*p* = 1.34e−4) and cognitive performance (*p* = 0.0076). However, the combined model was not significantly better than the model with only the PGS of intelligence (*p* = 0.069).

**Fig. 3:**
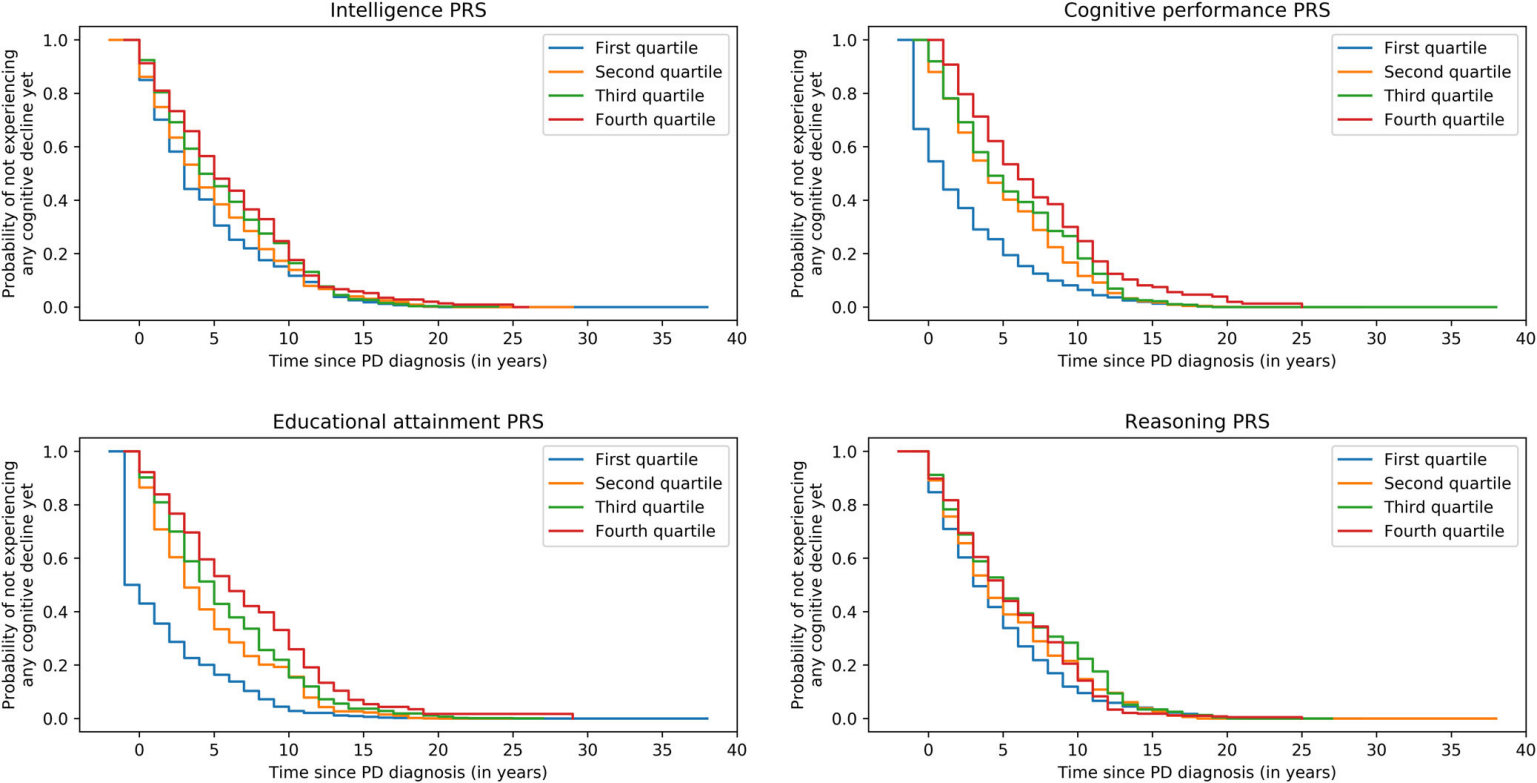
Survival plots. Survival plots for the four significant associations on the whole population (six cohorts merged).

Figure 3 highlights the survival plots on the whole population (six cohorts) for the four significant associations in the meta-analysis, with survivability being defined as not having a cognitive score below the defined cutoff values. Participants were grouped into four groups based on the quartiles of each PGS. Survival plots were significantly different between quartiles for the PGS of cognitive performance (*p* = 1.50e−4) and educational attainment (*p* = 1.68e−5), but not for the PGS of intelligence (*p* = 0.02) and reasoning (*p* = 0.02) after correction for multiple comparisons. Participants from a higher quartile tend to remain cognitively unimpaired longer than participants from a lower quartile, by the protective aspect of the four associations. The difference in years between the fourth and first quartile, for the probability of not experiencing any cognitive disorder yet equal to 0.5, was equal to 2 years for the PGS of intelligence, 5 years for cognitive performance, 7 years for educational attainment and 2 years for reasoning.

## Discussion

This study demonstrates that genetic variants linked to higher cognitive or educational performance in healthy individuals are also associated with reduced cognitive decline in PD.

We report four significant associations with PGS, all corresponding to phenotypes related to cognition. The results were consistent across the cohorts despite their heterogeneity in terms of cognitive scales used and baseline characteristics. Survival plots highlighted an offset of several years between the first and last quartiles of PGS, especially for the PGS of cognitive performance and educational attainment. Importantly, the known mutations in the *GBA* and *APOE* genes were not involved in the PGS computation, and the associations were corrected for these mutations, implying that these significant associations involve other genetic variants. These corrections may explain why we did not find any association with PGS of disease-related dementia phenotypes such as in Alzheimer’s disease (AD), AD or a family history of AD, and Lewy body dementia (LBD).

The causal relationships between genetic variants and multiple interrelated phenotypic traits are often complex. In principle, a genetic variant could increase cognitive reserve and thereby indirectly protect against cognitive decline in PD through mechanisms that are non-specific and potentially important long before PD onset. Patients with higher PGS for intelligence, cognitive performance, educational attainment, and reasoning will plausibly have had higher cognitive performance before the onset of PD-related pathology. Alternatively, variants promoting cognition in healthy individuals might also act directly on molecular disease pathways over the course of PD. Our study was not designed to differentiate between these different modes of action. If there had been available data in our PD cohorts to adjust for educational attainment or cognitive performance early in life for instance, it could have indicated of whether these variables in themselves fully account for the difference in PD cognitive outcome, or if the PGS makes an additional, independent contribution. It seems likely, however, that differences in the rate of neuropathological change is not the main driver, and that the significant PGS in our study can be thought as a proxy for cognitive reserve.

Cognitive reserve focuses on the idea that there are individual differences in adaptability of functional brain processes that allow some people to cope better than others with age- and disease-related brain change^48^. Higher cognitive reserve has been suggestively associated with better cognitive function and lower risk of longitudinal progression to mild cognitive impairment in PD^49^, notably as cognitive reserve may have greater effects on the cognitive areas mostly affected in PD^50^. Higher cognitive reserve has also been suggestively associated with fewer motor symptoms in PD^51^. Nonetheless, further studies are required to investigate the impact of cognitive reserve on PD progression.

We acknowledge that the observed associations are not necessarily specific to PD, and we do not know whether the prognostic value of these PGS extends beyond what could be captured equally well or even better with cognitive assessments. Such assessments are resource-demanding, however, and not practical as an initial screening in large cohorts. Regardless of the causal relationship, the PGS highlighted in our study provides valid information on a PD participant’s risk of cognitive decline, without the need to measure cognitive reserve, suggesting a potential tool for risk stratification.

We did not observe any significant association with PGS of brain imaging phenotypes. However, the PGS of the cortical surface area in the whole brain was close to significance. Even though DIGPD was not included in this meta-analysis due to the different cognitive scales used to assess cognition, this PGS also had one of the lowest *p*-values in this cohort (although not being significant after Bonferroni correction). In addition, the directions were all identical (except in the Iceberg cohort, but the effect was very close to zero) with a protective effect (the higher the PGS, the higher the cognitive scores, thus the less cognitive decline). Associations with the real (not PGS) phenotypes have been reported in the literature, with cortical thickness in the left caudal anterior cingulate, lateral occipital and right superior temporal areas being thinner in participants with mild cognitive impairment than normal older adults^52^. In our study, PGS associated with brain imaging features represented the majority of the PGS investigated, leading to a lower significance threshold to account for multiple comparisons and, thus less power. Further studies with larger sample sizes or fewer phenotypes are required to draw conclusions regarding the PGS of these phenotypes.

We did not either observe any significant association with the PGS of AD, AD or family history of AD, LBD and PD, whether the *APOE* and *GBA* covariates were included or not in the models. Nonetheless, the *p*-values of the three coefficients for the PGS of AD, AD or family history of AD, and LBD were smaller when excluding the *APOE* and *GBA* covariates, and the associations would have been significant without correction for multiple comparisons. These results show that these PGS still capture some information about the APOE and GBA status, although these variants were not included in the computation of the PGS, which might be explained by the inclusion of variants in linkage disequilibrium in the PGS computation. On the other hand, the PGS of PD was far from being significantly associated with cognitive scores, with and without *APOE* and *GBA* covariates. These results suggest that the genetics of cognitive decline in PD might be more related to the genetics of cognitive decline in general than the genetics of PD.

Our study has limitations. The total sample size is still relatively small and limits the statistical power to detect weaker associations. The variable size of the different GWAS used to compute PGS is another limitation since PGS are imperfect predictors of the genetic liability of phenotypes. Imputation may introduce noise in the PGS calculation. Nonetheless, the quality control based on the PGS of height and body mass index suggests good PGS computation (relative to the SNP heritability of each phenotype) even in the cohorts with imputed genotype data. Our effect sizes obtained were heterogeneous, which might be explained by the heterogeneity between cohorts. Our approach does not allow for identifying individual genetic variants associated with the phenotype of interest (cognitive decline in PD in our case) which is inherent to the methodology. We only performed a meta-analysis and did not perform any replication analysis in external cohorts, nor compare our results to the potential effect of these PGS in the general healthy population. The definition of cognitive decline based on cognitive score cut-offs is suboptimal, and additional assessment is further required for better diagnosis.

Our study identifies associations between cognitive scores in PD and PGS of several cognitive phenotypes, with higher PGS of cognitive phenotypes being associated with reduced cognitive impairment in PD. The real phenotypes and their PGS have also been associated with cognitive decline in the general population, suggesting genetic similarity between cognitive decline in PD and in the general population, and supporting the importance of the cognitive reserve in the susceptibility to cognitive decline in PD.

## Methods

### Populations

We used data from six research cohorts, including the Drug Interaction with Genes in Parkinson’s Disease (DIGPD) study^53^, the Iceberg study^54^, and four cohorts from the Accelerating Medicines Partnership® Parkinson’s disease (AMP PD) program^55^: the Parkinson’s Progression Markers Initiative (PPMI), the National Institute of Neurological Disorders and Stroke Parkinson’s Disease Biomarkers Program (PDBP), the Study of Urate Elevation in Parkinson’s disease (SURE-PD3), and the LRRK2 Cohort Consortium (LCC).

DIGPD is a French multicenter longitudinal cohort with annual follow-up of PD patients. Eligible criteria consist in recent PD diagnosis (UK Parkinson’s Disease Society Brain Bank criteria) with a disease duration of less than 5 years at recruitment. Data was gathered during face-to-face visits every 12 months following standard procedures.

Iceberg is a French longitudinal cohort with annual follow-up of idiopathic PD patients, patients with a genetic form of PD, and patients with idiopathic rapid eye movement sleep disorders. Data was gathered during face-to-face visits every 12 months following standard procedures.

PPMI is a multicenter observational clinical study using advanced imaging, biologic sampling and clinical and behavioral assessments to identify biomarkers of PD progression. Data was gathered during face-to-face visits every 6-12 months. PD subjects were de-novo and drug-naïve at baseline.

PDBP is an American clinical study developed to accelerate the discovery of promising new diagnostic and progression biomarkers for Parkinson’s disease.

SURE-PD3 is a randomized, double-blind, placebo-controlled trial of urate-elevating inosine treatment to slow clinical decline in early PD.

LCC consists of three closed studies: the LRRK2 cross-sectional study, the LRRK2 longitudinal study and the 23andMe Blood Collection Study. The LCC followed standardized data acquisition protocols.

All studies were conducted according to good clinical practice, obtained approval from local ethic committees and regulatory authorities, and all participants provided informed consent before inclusion.

### Key inclusion criteria

Further details of inclusion and exclusion criteria for AMP-PD cohorts can be found at https://amp-pd.org/unified-cohorts. As several of these studies included multiple study arms, only the inclusion criteria for the idiopathic PD study arm are summarized here as these are the patients we included for our analysis.

- **PPMI**: PD subjects must have 2 of the following symptoms: resting tremor, bradykinesia, rigidity, OR either asymmetric resting tremor or asymmetric bradykinesia. PD participants were required to be 30 years or older at time of PD diagnosis, have a diagnosis of PD for 2 years or less at screening, Hoehn and Yahr stage I or II at baseline, confirmation of dopamine transporter deficit by DaTSCAN, and not expected to require PD medication for at least 6 months from baseline visit.
- **SURE-PD3**: PD subjects had to fulfill diagnostic criteria for idiopathic PD with at least 2 of the cardinal signs of PD (resting tremor, bradykinesia, and rigidity), Hoehn and Yahr stage 1 to 2.5 (inclusive), absence of current or imminent PD disability requiring dopaminergic therapy (within 90 days of enrollment), aged 30 years or older at time of PD diagnosis, a diagnosis of PD within 3 years prior to the screening visit, and non-fasting serum urate ≤ 5.7 mg/dL at the first screening visit.
- **LCC**: Idiopathic PD participants were eligible for this study if they were of Ashkenazi Jewish (AJ) descent and have PD or parkinsonism, aged 18 years or older, and no history of neurological or psychological illness.
- **PDBP**: Clinically diagnosed with PD and aged 21 years or older.
- **DIGPD:** Subjects were recruited in this longitudinal multicenter study at 4 University Hospitals and 4 General hospital in France between 2009 and 2013 and followed annually for up to 7 years. Inclusion criteria were patients with a diagnosis of Parkinson’s disease according to the UK Parkinson’s disease Society Brain Bank criteria with a disease duration of less than 6 years at baseline. Exclusion criteria were atypical parkinsonism or a history of treatment with neuroleptics. Patients for whom the diagnosis was revised to atypical parkinsonism during the follow-up of the study were excluded from the analysis. A complete description of the population is available elsewhere^53^.
- **Iceberg:** This is an ongoing monocenter longitudinal clinical study conducted at the Pitié-Salpêtrière Hospital, Paris, France. Inclusion criteria for PD patients were a diagnosis of Parkinson’s disease according to UK Parkinson’s Disease Society Brain Bank criteria with a disease duration of less than 4 years at baseline. Exclusion criteria were atypical parkinsonism such as multiple system atrophy, supranuclear palsy, dementia with Lewy bodies, or a history of treatment with neuroleptics.

### Participants

For our analysis, inclusion criteria consisted of having (i) a PD diagnosis, (ii) at least one visit assessing cognition with a cognitive scale, and (iii) genetic data available. Participants recruited in the genetically enriched arms (for carrying specific genetic mutations) of any cohort were excluded. Cognition was assessed using the Mini-Mental State Examination (MMSE) in DIGPD and the Montreal Cognitive Assessment (MoCA) in the other cohorts. As a measure of cognitive outcome, we used time from diagnosis to MMSE ≤ 27, or MoCA ≤ 24, as previously proposed as cut-off to define mild cognitive impairment in PD^56^.

### Genotyping and quality control

Genotype data were acquired using Illumina Multi-Ethnic Genotyping Arrays in the DIGPD cohort (1,779,819 variants), Illumina NeurochipHumanCore-24-v1_A Genotyping Arrays in the Iceberg cohort (487,687 variants) and Illumina HiSeq XTen sequencer in the AMP PD cohorts (whole genome).

Standard quality control steps were performed in each cohort using PLINK^57^. We excluded variants with missing rates greater than 2% and variants deviating from Hardy-Weinberg equilibrium (*p* < 1e−8). We excluded related individuals (third-degree family relationships), individuals with mismatching between reported sex and genetically determined sex, and individuals with outlying heterozygosity (±3 standard deviations). For cohorts without whole-genome sequencing (DIGPD and Iceberg), we imputed missing SNPs using the Sanger Imputation Server^58^ for DIGPD and the Michigan Imputation Server^59^ for Iceberg, using the reference panel of the Haplotype Reference Consortium (release 1.1)^58^, then selected SNPs that were imputed with sufficient accuracy (INFO Score > 0.9 for DIGPD, R2 > 0.7 for Iceberg).

### Genetic ancestry

To estimate the genetic ancestry of the participants, we used raw genotype data from the HapMap3 project to learn a low-dimensional representation of the genetic data, which captures the main dimension of ancestry, using principal component analysis. We then projected the raw genotype data of the participants onto the main principal components to identify in which clusters the participants were the closest to. Participants projected too far away (further than 6 standard deviations) from the European cluster were excluded. In further analyses, genetic ancestry was defined as the first four components of the principal component analysis.

### *GBA* and *APOE* mutations

Specific *GBA* sequencing was performed in DIGPD and Iceberg. *GBA* mutations were extracted from such sequencing in DIGPD and Iceberg. For AMP PD cohorts, *GBA* mutations were extracted from whole-genome sequencing although this method could not formally distinguish these variants from variants of the pseudogene. *GBA* mutations were classified based on their association with PD severity^60^ and the numbers of mild, severe and undetermined *GBA* mutations were respectively computed.

The two SNPs involved in the *APOE* allelic variants associated with modified risks of developing Alzheimer’s disease (rs7412 and rs429358)^61^ were extracted from raw genotype data if available or from imputed genotype data otherwise.

### Phenotypes and genome-wide association studies

We used the NHGRI-EBI GWAS Catalog^62^ to select the largest GWAS to date on samples of European ancestry for each phenotype of interest. From this database, we selected all phenotypes based on their known or putative implication as factors clinically associated with cognitive decline in PD and the general population, such as educational attainment, stroke, and Alzheimer’s disease (AD). A total of 19 such phenotypes were selected among 12 available GWAS. In addition, we selected the 79 brain anatomical phenotypes in all GWAS (such as white matter hyperintensities, subcortical volumes as well as cortical surface areas and thicknesses in several regions of the brain), as there is growing evidence of associations with brain anatomical phenotypes in PD^63^ and the general population^52^. Finally, two more general phenotypes (height and body mass index) were also considered, not only because height has been inversely associated with dementia in men^64^, but also because the real phenotypes were available and could be used as a sanity check of our methodology by assessing the quality of the computed PGS for these phenotypes.

Altogether, a total of 100 phenotypes were selected for this analysis among the 18 GWAS available in this database. When summary statistics from several GWAS were available for a given phenotype, we only included the largest study.

### Polygenic scores

We used the LDpred2 algorithm^65^ implemented in the *bigsnpr* R package to compute all the PGS. More precisely, we used the LDpred2-auto variant which does not require any tuning samples^65^. This criterion was necessary as we computed PGS for phenotypes that were not assessed (i.e., the real phenotypes were not available).

The objective of the algorithm is to derive the joint effects (i.e., the coefficients in the PGS computation) from the marginal effects (i.e., the coefficients from the summary statistics of a GWAS). We used the linkage disequilibrium (LD) reference provided in the software, which is computed based on genetic data of 362,320 individuals enrolled in the UK BioBank study. The list of SNPs used to compute each PGS in each cohort consisted of the intersection of (i) the list of SNPs available in the given cohort, (ii) the list of SNPs in the LD reference (i.e., the list of SNPs from the HapMap3 project) and (iii) the list of SNPs in the summary statistics of the given GWAS, minus the SNPs matching exclusion criteria as recommended in the quality control step preceding the LDpred2 algorithm. No SNP is excluded based on their *p*-value with the LDpred2 algorithm: the *p*-value is used as a confidence measure of the marginal effect when deriving the joint effect. Such methods have been proven to generally perform better than clumping & thresholding^65,66^. None of the extracted mutations in the *GBA* and *APOE* genes were included in the PGS computation, as they are not part of the list of SNPs from the HapMap3 project.

### Statistical analyses

Participants’ characteristics in all the cohorts were compared with chi-squared tests for categorical variables and analysis of variance F-tests for continuous variables. The quality of the height and body mass index PGS was assessed using partial correlation coefficients with correction for sex, age, age at PD diagnosis, and genetic ancestry. Longitudinal analyses were performed using linear fixed effects models to investigate associations between cognitive scores and each PGS, with correction for age at PD diagnosis, sex, time from PD onset, genetic ancestry, number of mild, severe, and undetermined *GBA* mutations, and *APOE* status. Visits with any missing clinical value among the variables used in the longitudinal analyses were excluded. Meta-analysis for cohorts using the same cognitive screening test was performed with linear fixed effects models. The usual 0.05 threshold was used to determine the significance of any statistical test, and per-sample Bonferroni correction for multiple comparisons was applied, leading to a significance threshold of 0.0005 for potential GWAS associations (100 GWAS included, see Results). Associations for significant PGS for the meta-analysis were visually inspected using forest plots. Survival plots were generated for such PGS, grouping participants each time into four groups (corresponding to the four quartiles for each PGS), and groups were compared using the log-rank test.

### Reporting summary

Further information on research design is available in the Nature Research Reporting Summary linked to this article.

### Supplementary information

Supplementary Information

Reporting summary

## Data Availability

The datasets generated and analyzed during the current study are available from the corresponding author upon request (jean-christophe.corvol@aphp.fr). The genotype and clinical data for the AMP PD cohorts (PPMI, PDBP, Sure-PD3 and LCC) are available through the Accelerating Medicine Partnership® (AMP®) Parkinson’s Disease (AMP PD) Knowledge Platform. For up-to-date information on the study, visit https://www.amp-pd.org. Clinical longitudinal data and genotyping data for the other cohorts included are accessible through appropriate data-sharing agreements that protect participant privacy with the institutions that conducted or are conducting study consents and clinical assessments under local institutional review board approvals.

## Code availability

The source code associated with the analyses performed is publicly available at https://github.com/johannfaouzi/genetics_cognitive_decline_PD.

## References

[1] Litvan I (2011). MDS task force on mild cognitive impairment in Parkinson’s disease: Critical review of PD-MCI. Mov. Disord..

[2] Baiano C, Barone P, Trojano L, Santangelo G (2020). Prevalence and clinical aspects of mild cognitive impairment in Parkinson’s disease: A meta-analysis. Mov. Disord..

[3] Levy G (2002). The association of incident dementia with mortality in PD. Neurology.

[4] Aarsland D (2017). Cognitive decline in Parkinson’s disease. Nat. Rev. Neurol..

[5] Fagan ES, Pihlstrøm L (2017). Genetic risk factors for cognitive decline in Parkinson’s disease: a review of the literature. Eur. J. Neurol..

[6] Hruska KS, LaMarca ME, Scott CR, Sidransky E (2008). Gaucher disease: mutation and polymorphism spectrum in the glucocerebrosidase gene (GBA). Hum. Mutat..

[7] Alcalay RN (2012). Cognitive performance of GBA mutation carriers with early-onset PD. Neurology.

[8] Setó-Salvia N (2012). Glucocerebrosidase mutations confer a greater risk of dementia during Parkinson’s disease course. Mov. Disord..

[9] Winder-Rhodes SE (2013). Glucocerebrosidase mutations influence the natural history of Parkinson’s disease in a community-based incident cohort. Brain.

[10] Mata IF (2016). GBA Variants are associated with a distinct pattern of cognitive deficits in Parkinson’s disease. Mov. Disord..

[11] Liu G (2021). Genome-wide survival study identifies a novel synaptic locus and polygenic score for cognitive progression in Parkinson’s disease. Nat. Genet.

[12] Yamazaki Y (2019). and Alzheimer disease: pathobiology and targeting strategies. Nat. Rev. Neurol..

[13] Williams-Gray CH (2009). Apolipoprotein E genotype as a risk factor for susceptibility to and dementia in Parkinson’s disease. J. Neurol..

[14] Nombela C (2014). Genetic impact on cognition and brain function in newly diagnosed Parkinson’s disease: ICICLE-PD study. Brain.

[15] Morley JF (2012). Genetic influences on cognitive decline in Parkinson’s disease. Mov. Disord..

[16] Mata IF (2014). APOE, MAPT, and SNCA genes and cognitive performance in Parkinson’s disease. JAMA Neurol..

[17] Paul KC (2016). APOE, MAPT, and COMT and Parkinson’s disease susceptibility and cognitive symptom progression. J. Parkinsons Dis..

[18] Srivatsal S (2015). Cognitive profile of LRRK2-related Parkinson’s disease. Mov. Disord..

[19] Shanker V (2011). Mood and cognition in Leucine-rich repeat Kinase 2 G2019S Parkinson’s disease. Mov. Disord..

[20] Ben Sassi S (2012). Cognitive dysfunction in Tunisian LRRK2 associated Parkinson’s disease. Parkinsonism Relat. Disord..

[21] Somme JH (2011). Initial neuropsychological impairments in patients with the E46K mutation of the α-synuclein gene (PARK 1). J. Neurol. Sci..

[22] Svetel M (2013). No association between brain-derived neurotrophic factor G196A polymorphism and clinical features of Parkinson’s disease. Eur. Neurol..

[23] Białecka M (2014). BDNF G196A (Val66Met) polymorphism associated with cognitive impairment in Parkinson’s disease. Neurosci. Lett..

[24] Iwaki H (2019). Genomewide association study of Parkinson’s disease clinical biomarkers in 12 longitudinal patients’ cohorts. Mov. Disord..

[25] Park KW (2020). Genomic association study for cognitive impairment in Parkinson’s disease. Front Neurol..

[26] Tan MMX (2021). Genome-wide association studies of cognitive and motor progression in Parkinson’s disease. Mov. Disord..

[27] Rongve A (2019). GBA and APOE ε4 associate with sporadic dementia with Lewy bodies in European genome wide association study. Sci. Rep..

[28] Jo S (2021). Microarray genotyping identifies new Loci associated with Dementia in Parkinson’s disease. Genes.

[29] Liu G (2023). Mitochondrial haplogroups and cognitive progression in Parkinson’s disease. Brain.

[30] Yengo L (2018). Meta-analysis of genome-wide association studies for height and body mass index in ∼700,000 individuals of European ancestry. Hum. Mol. Genet..

[31] Pulit SL (2019). Meta-analysis of genome-wide association studies for body fat distribution in 694 649 individuals of European ancestry. Hum. Mol. Genet..

[32] Davies G (2016). Genome-wide association study of cognitive functions and educational attainment in UK Biobank (N=112151). Mol. Psychiatry.

[33] Davies G (2018). Study of 300,486 individuals identifies 148 independent genetic loci influencing general cognitive function. Nat. Commun..

[34] Lee JJ (2018). Gene discovery and polygenic prediction from a genome-wide association study of educational attainment in 1.1 million individuals. Nat. Genet..

[35] Savage JE (2018). Genome-wide association meta-analysis in 269,867 individuals identifies new genetic and functional links to intelligence. Nat. Genet..

[36] Nalls MA (2019). Identification of novel risk loci, causal insights, and heritable risk for Parkinson’s disease: a meta-analysis of genome-wide association studies. Lancet Neurol..

[37] Kunkle BW (2019). Genetic meta-analysis of diagnosed Alzheimer’s disease identifies new risk loci and implicates Aβ, tau, immunity and lipid processing. Nat. Genet..

[38] Schwartzentruber J (2021). Genome-wide meta-analysis, fine-mapping and integrative prioritization implicate new Alzheimer’s disease risk genes. Nat. Genet..

[39] Chia R (2021). Genome sequencing analysis identifies new loci associated with Lewy body dementia and provides insights into its genetic architecture. Nat. Genet..

[40] Malik R (2018). Multiancestry genome-wide association study of 520,000 subjects identifies 32 loci associated with stroke and stroke subtypes. Nat. Genet..

[41] Howard DM (2019). Genome-wide meta-analysis of depression identifies 102 independent variants and highlights the importance of the prefrontal brain regions. Nat. Neurosci..

[42] Otowa T (2016). Meta-analysis of genome-wide association studies of anxiety disorders. Mol. Psychiatry.

[43] Neale lab. UK Biobank. *Neale lab* http://www.nealelab.is/uk-biobank (2018).

[44] Sargurupremraj M (2020). Cerebral small vessel disease genomics and its implications across the lifespan. Nat. Commun..

[45] Adams HHH (2016). Novel genetic loci underlying human intracranial volume identified through genome-wide association. Nat. Neurosci..

[46] Satizabal CL (2019). Genetic architecture of subcortical brain structures in 38,851 individuals. Nat. Genet..

[47] Grasby KL (2020). The genetic architecture of the human cerebral cortex. Science.

[48] Perneczky R (2019). Translational research on reserve against neurodegenerative disease: consensus report of the International Conference on Cognitive Reserve in the Dementias and the Alzheimer’s Association Reserve, Resilience and Protective Factors Professional Interest Area working groups. BMC Med.

[49] Gu L, Xu H (2022). Effect of cognitive reserve on cognitive function in Parkinson’s disease. Neurol. Sci..

[50] Ciccarelli N (2018). The role of cognitive reserve in cognitive aging: what we can learn from Parkinson’s disease. Aging Clin. Exp. Res.

[51] Lee P-C (2019). Examining the reserve hypothesis in Parkinson’s disease: a longitudinal study. Mov. Disord..

[52] Cheng CP-W (2018). Relationship between cortical thickness and neuropsychological performance in normal older adults and those with mild cognitive impairment. Aging Dis..

[53] Corvol J-C (2018). Longitudinal analysis of impulse control disorders in Parkinson’s disease. Neurology.

[54] Czernecki V (2021). Social cognitive impairment in early Parkinson’s disease: A novel “mild impairment”?. Parkinsonism Relat. Disord..

[55] Iwaki H (2021). Accelerating medicines partnership: Parkinson’s disease. Genetic resource. Mov. Disord..

[56] Hoops S (2009). Validity of the MoCA and MMSE in the detection of MCI and dementia in Parkinson’s disease. Neurology.

[57] Chang CC (2015). Second-generation PLINK: rising to the challenge of larger and richer datasets. Gigascience.

[58] McCarthy S (2016). A reference panel of 64,976 haplotypes for genotype imputation. Nat. Genet..

[59] Das S (2016). Next-generation genotype imputation service and methods. Nat. Genet..

[60] Höglinger G (2022). GBA-associated PD: chances and obstacles for targeted treatment strategies. J. Neural Transm..

[61] Liu C-C, Kanekiyo T, Xu H, Bu G, Apolipoprotein E (2013). and Alzheimer disease: risk, mechanisms and therapy. Nat. Rev. Neurol..

[62] Buniello A (2019). The NHGRI-EBI GWAS Catalog of published genome-wide association studies, targeted arrays and summary statistics 2019. Nucleic Acids Res.

[63] Sasikumar S, Strafella AP (2020). Imaging mild cognitive impairment and dementia in Parkinson’s disease. Front Neurol..

[64] Jørgensen TSH, Okholm GT, Christensen K, Sørensen TI, Osler M (2020). Body height in young adult men and risk of dementia later in adult life. eLife.

[65] Privé F, Arbel J, Vilhjálmsson BJ (2020). LDpred2: better, faster, stronger. Bioinformatics.

[66] Ni G (2021). A comparison of ten polygenic score methods for psychiatric disorders applied across multiple cohorts. Biol. Psychiatry.

