# Supplementary material for: Proxy-analysis of the genetics of cognitive decline in Parkinson’s disease through polygenic scores
Source: NPJ Parkinsons Dis. 2024 Jan 4;10:8. doi: 10.1038/s41531-023-00619-5 (PMC10767119; doi:10.1038/s41531-023-00619-5)
Supplement: Supplementary file 1 — Supplementary Information [file 41531_2023_619_MOESM1_ESM.pdf]

Supplementary Figure 1

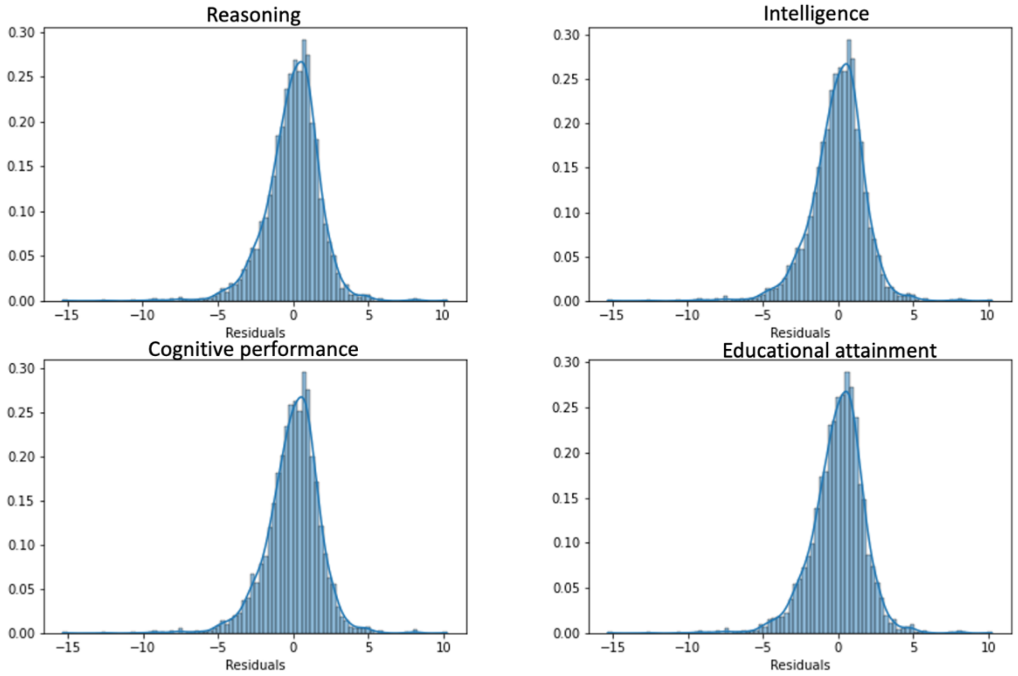

Distribution of the models' residuals for the PGS of reasoning, intelligence, cognitive performance and educational attainment ($p < 0.05$) in PPMI.

Supplementary Figure 2

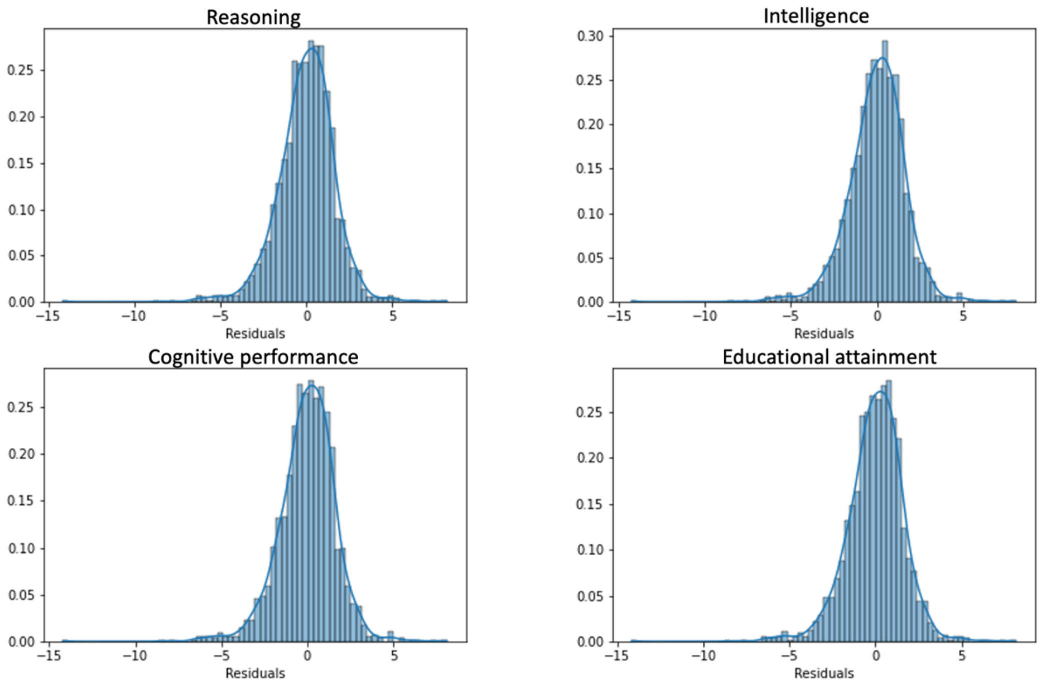

Distribution of the models' residuals for the PGS of reasoning, intelligence, cognitive performance and educational attainment ($p < 0.05$) in PDBP.

Supplementary Figure 3

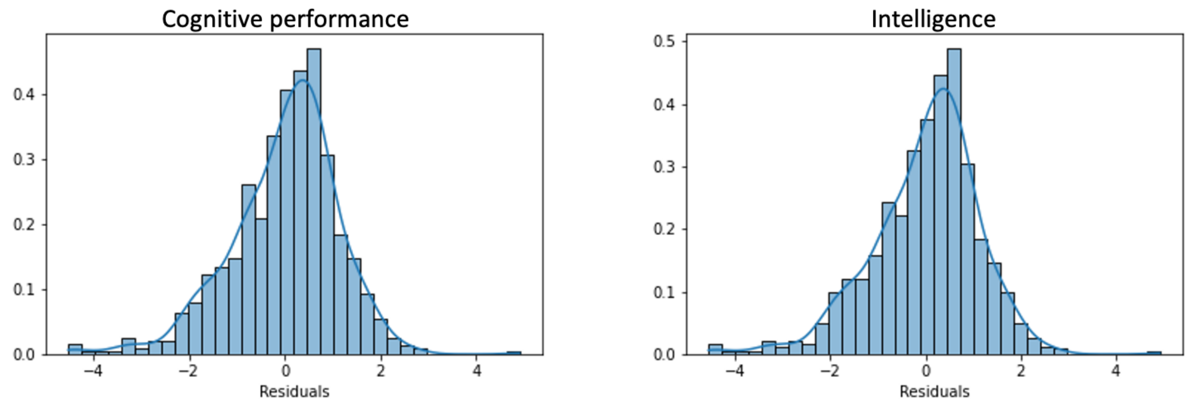

Distribution of the models' residuals for the PGS of intelligence and cognitive performance ($p < 0.05$) in SURE-PD3.

Supplementary Figure 4

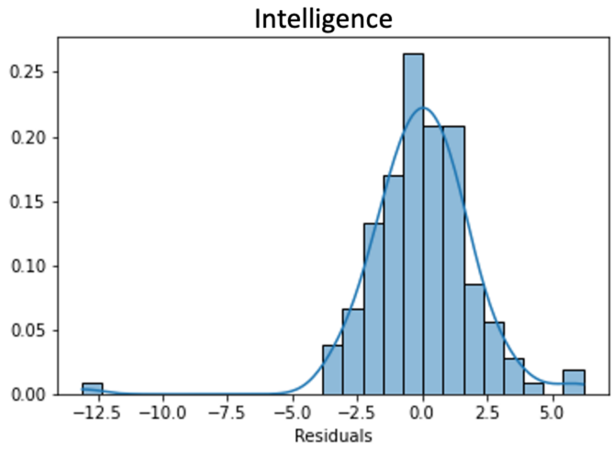

Distribution of the models' residuals for the PGS of intelligence ($p < 0.05$) in LCC.

Supplementary Table 1

chromosome	base pair	A1	A2	id	rsid	Severity
1	155235196	A	G	1:155235196:G:A	rs80356771	Severe
1	155235203	G	C	1:155235203:C:G	rs1135675	Unknown
1	155235217	G	C	1:155235217:C:G	rs368060	Severe
1	155235227	A	G	1:155235227:G:A	rs149257166	Unknown
1	155235252	A	G	1:155235252:G:A	rs421016	Severe
1	155235409	T	G	1:155235409:G:T	rs183510604	Unknown
1	155235587	T	C	1:155235587:C:T	rs12752133	Unknown
1	155235772	A	C	1:155235772:C:A	rs80356769	Severe
1	155235843	C	T	1:155235843:T:C	rs76763715	Mild
1	155235847	G	A	1:155235847:A:G	rs377143075	Unknown
1	155235990	G	T	1:155235990:T:G	rs1178983093	Unknown
1	155236102	C	T	1:155236102:T:C	rs758462709	Unknown
1	155236109	A	T	1:155236109:T:A	rs538683487	Unknown
1	155236245	T	C	1:155236245:C:T	rs138498426	Unknown
1	155236246	A	G	1:155236246:G:A	rs75548401	Unknown
1	155236269	T	C	1:155236269:C:T	rs149487315	Unknown
1	155236376	T	C	1:155236376:C:T	rs2230288	Unknown
1	155236550	G	T	1:155236550:T:G	rs9628662	Unknown
1	155236582	C	T	1:155236582:T:C	rs568893377	Unknown
1	155236789	G	A	1:155236789:A:G	rs146697312	Unknown
1	155236794	A	T	1:155236794:T:A	rs191036459	Unknown
1	155237239	A	G	1:155237239:G:A	rs72704130	Unknown
1	155237438	T	C	1:155237438:C:T	rs140955685	Unknown
1	155237453	T	C	1:155237453:C:T	rs78973108	Severe
1	155237576	T	A	1:155237576:A:T	rs74500255	Mild
1	155237596	T	A	1:155237596:A:T	rs140335079	Unknown
1	155237731	A	G	1:155237731:G:A	rs1345592581	Unknown
1	155237758	C	T	1:155237758:T:C	rs762488	Unknown
1	155237789	T	C	1:155237789:C:T	rs938033488	Unknown
1	155237835	A	G	1:155237835:G:A	rs2009578	Unknown
1	155237942	T	C	1:155237942:C:T	rs28678003	Unknown
1	155238055	C	T	1:155238055:T:C	rs145066479	Unknown
1	155238057	T	G	1:155238057:G:T	rs183540501	Unknown
1	155238174	G	A	1:155238174:A:G	rs409652	Severe
1	155238200	T	C	1:155238200:C:T	rs1376479747	Unknown
1	155238631	A	G	1:155238631:G:A	rs147411159	Unknown
1	155238820	G	A	1:155238820:A:G	rs569282073	Unknown
1	155238833	G	A	1:155238833:A:G	rs188328778	Unknown
1	155239060	C	T	1:155239060:T:C	rs149120852	Unknown
1	155239258	A	G	1:155239258:G:A	rs531177999	Unknown
1	155239288	A	G	1:155239288:G:A	rs114452199	Unknown
1	155239385	T	C	1:155239385:C:T	rs1370900181	Unknown
1	155239506	T	C	1:155239506:C:T	rs183903019	Unknown
1	155239569	T	C	1:155239569:C:T	rs2075569	Unknown
1	155239961	A	G	1:155239961:G:A	rs146774384	Unknown
1	155240122	G	T	1:155240122:T:G	rs199565854	Unknown
1	155240171	T	C	1:155240171:C:T	rs114217696	Unknown
1	155240239	A	G	1:155240239:G:A	rs151028758	Unknown
1	155240336	T	C	1:155240336:C:T	rs142348200	Unknown
1	155240355	T	C	1:155240355:C:T	rs1305943514	Unknown
1	155240379	A	G	1:155240379:G:A	rs962460364	Unknown
1	155240549	T	C	1:155240549:C:T	NA	Unknown
1	155240707	C	T	1:155240707:T:C	rs150466109	Unknown
1	155240779	C	T	1:155240779:T:C	rs2361534	Unknown
1	155240850	C	A	1:155240850:A:C	rs2070679	Unknown
1	155241127	C	T	1:155241127:T:C	rs41264927	Unknown
1	155241231	C	T	1:155241231:T:C	rs759420154	Unknown
1	155241315	C	T	1:155241315:T:C	rs188978150	Unknown
1	155241932	C	A	1:155241932:A:C	rs1053562776	Unknown
1	155241946	A	G	1:155241946:G:A	rs893532874	Unknown
1	155242309	G	A	1:155242309:A:G	rs1800442	Unknown
1	155242370	T	C	1:155242370:C:T	rs569157832	Unknown
1	155242386	A	C	1:155242386:C:A	rs538272827	Unknown
1	155242419	T	C	1:155242419:C:T	rs989161650	Unknown
1	155242420	G	A	1:155242420:A:G	rs3754485	Unknown
1	155242425	A	G	1:155242425:G:A	rs534257059	Unknown
1	155242497	T	C	1:155242497:C:T	rs1800438	Unknown
1	155242531	G	C	1:155242531:C:G	rs1048398656	Unknown
1	155242601	G	T	1:155242601:T:G	rs768531904	Unknown
1	155242734	G	A	1:155242734:A:G	rs192028662	Unknown
1	155242881	T	C	1:155242881:C:T	rs919059358	Unknown
1	155242917	G	A	1:155242917:A:G	rs766355861	Unknown
1	155243200	A	G	1:155243200:G:A	rs118125215	Unknown
1	155243333	A	T	1:155243333:T:A	rs11264345	Unknown
1	155243793	A	G	1:155243793:G:A	rs184145634	Unknown
1	155243797	T	C	1:155243797:C:T	rs150974694	Unknown
1	155244065	G	T	1:155244065:T:G	rs190716448	Unknown
1	155244216	A	G	1:155244216:G:A	rs149732676	Unknown
1	155244242	C	T	1:155244242:T:C	rs779921478	Unknown
1	155244275	T	C	1:155244275:C:T	rs10908459	Unknown
1	155244372	T	C	1:155244372:C:T	rs552066784	Unknown
1	155244413	A	T	1:155244413:T:A	rs774329309	Unknown

Supplementary Table 2

Study	Phenotype	SNP heritability	Number of subjects	Number of SNPs
Adams et al., 2016	Intracranial volume	0.2484	26,577	9,702,043
Chia et al., 2021	Lewy body dementia	0.1081	6,618	7,843,595
Davies et al., 2016	Memory performance	0.05	112,067	17,344,579
	Reasoning	0.31	36,035	17,361,492
Davies et al., 2018	Reaction time	0.0742	330,069	18,485,867
Grasby et al., 2020	Banks of the superior temporal sulcus cortical surface area	0.1513	51,665	8,562,600
	Banks of the superior temporal sulcus cortical thickness	0.0338	51,665	8,575,874
	Caudal anterior cingulate cortical surface area	0.161	51,665	8,606,348
	Caudal anterior cingulate cortical thickness	0.0541	51,665	8,615,904
	Caudal middle frontal cortical surface area	0.1949	51,665	8,604,516
	Caudal middle frontal cortical thickness	0.0854	51,665	8,613,442
	Cuneus cortical surface area	0.175	51,665	8,607,614
	Cuneus cortical thickness	0.0643	51,665	8,604,723
	Entorhinal cortical surface area	0.1579	51,665	8,535,097
	Entorhinal cortical thickness	0.049	51,665	8,568,396
	Frontal pole cortical surface area	0.0826	51,665	8,616,334
	Frontal pole cortical thickness	0.0132	51,665	8,616,589
	Whole cortical surface area	0.34	51,665	8,567,615
	Whole cortical thickness	0.26	51,665	8,619,054
	Fusiform cortical surface area	0.1726	51,665	8,584,552
	Fusiform cortical thickness	0.0488	51,665	8,608,485
	Inferior parietal cortical surface area	0.1926	51,665	8,590,146
	Inferior parietal cortical thickness	0.0985	51,665	8,603,794
	Inferior temporal cortical surface area	0.1842	51,665	8,601,235
	Inferior temporal cortical thickness	0.0793	51,665	8,603,070
	Insula cortical surface area	0.2384	51,665	8,590,192
	Insula cortical thickness	0.0864	51,665	8,566,707
	Isthmus cingulate cortical surface area	0.1467	51,665	8,588,468
	Isthmus cingulate cortical thickness	0.0986	51,665	8,609,094
	Lateral occipital cortical surface area	0.2173	51,665	8,606,805
	Lateral occipital cortical thickness	0.0863	51,665	8,603,758
	Lateral orbitofrontal cortical surface area	0.2522	51,665	8,614,256
	Lateral orbitofrontal cortical thickness	0.0465	51,665	8,578,266
	Lingual cortical surface area	0.2302	51,665	8,611,655
	Lingual cortical thickness	0.0817	51,665	8,604,808
	Medial orbitofrontal cortical surface area	0.1393	51,665	8,598,592
	Medial orbitofrontal cortical thickness	0.0523	51,665	8,587,654
	Middle temporal cortical surface area	0.2025	51,665	8,567,536
	Middle temporal cortical thickness	0.0837	51,665	8,581,180
	Paracentral cortical surface area	0.1587	51,665	8,600,953
	Paracentral cortical thickness	0.0834	51,665	8,613,261
	Para hippocampal cortical surface area	0.1489	51,665	8,448,047
	Para hippocampal cortical thickness	0.1064	51,665	8,530,381
	Pars opercularis cortical surface area	0.1549	51,665	8,606,052
	Pars opercularis cortical thickness	0.0448	51,665	8,613,538
	Pars orbitalis cortical surface area	0.1589	51,665	8,614,364
	Pars orbitalis cortical thickness	0.0109	51,665	8,615,116
	Pars triangularis cortical surface area	0.1974	51,665	8,608,759
	Pars triangularis cortical thickness	0.0712	51,665	8,611,582
	Pericalcarine cortical surface area	0.3102	51,665	8,608,441
	Pericalcarine cortical thickness	0.0525	51,665	8,588,778
	Postcentral cortical surface area	0.1692	51,665	8,579,710
	Postcentral cortical thickness	0.0964	51,665	8,596,913
	Posterior cingulate cortical surface area	0.1597	51,665	8,614,330
	Posterior cingulate cortical thickness	0.0707	51,665	8,609,400
	Precentral cortical surface area	0.1958	51,665	8,587,822
	Precentral cortical thickness	0.0835	51,665	8,604,415
	Precuneus cortical surface area	0.2077	51,665	8,612,181
	Precuneus cortical thickness	0.069	51,665	8,614,835
	Rostral anterior cingulate cortical surface area	0.1546	51,665	8,595,545
	Rostral anterior cingulate cortical thickness	0.0452	51,665	8,614,560
	Rostral middle frontal cortical surface area	0.2116	51,665	8,607,860
	Rostral middle frontal cortical thickness	0.066	51,665	8,594,930
	Superior frontal cortical surface area	0.1676	51,665	8,592,540
	Superior frontal cortical thickness	0.0844	51,665	8,594,825
	Superior parietal cortical surface area	0.1837	51,665	8,594,123
	Superior parietal cortical thickness	0.07	51,665	8,595,065
	Superior temporal cortical surface area	0.2637	51,665	8,550,865
	Superior temporal cortical thickness	0.1317	51,665	8,559,521
	Supramarginal cortical surface area	0.1626	51,665	8,569,308
	Supramarginal cortical thickness	0.1062	51,665	8,572,419
	Temporal pole cortical surface area	0.1241	51,665	8,599,231
	Temporal pole cortical thickness	0.0196	51,665	8,603,637
	Transverse temporal cortical surface area	0.227	51,665	8,615,820
	Transverse temporal cortical thickness	0.0966	51,665	8,609,408
Howard et al., 2019	Major depressive disorder	0.089	807,553	8,483,301
Kunkle et al., 2019	Alzheimer's disease	0.071	63,926	11,480,632
Lee et al., 2018	Cognitive performance	0.209	257,828	10,098,325
	Educational attainment	0.122	766,345	10,101,242
Malik et al., 2018	Any ischemic stroke	0.0135	440,328	8,340,184
	Any stroke	0.0127	446,696	8,255,860
	Cardioembolic stroke	0.0147	211,763	8,306,090
	Large artery stroke	0.0015	150,765	8,451,005
	Small vessel stroke	0.0058	198,048	8,765,828
Nalls et al., 2019	Parkinson's disease or first degree relation to individual with Parkinson's disease	0.22	482,730	17,510,617
Neale lab, 2018	Sleeplessness or insomnia	0.0624	360,738	13,791,467
	Trouble falling asleep	0.0581	117,822	13,791,467
Otowa et al., 2016	Anxiety disorder	0.138	1,731	6,330,995
Pulit et al., 2019	Body mass index	0.279	806,834	27,381,302
Sargurupremraj et al., 2020	White matter hyperintensities	0.54	48,454	6,334,466
Satizabal et al., 2019	Nucleus accumbens subcortical volume	0.191	32,562	7,563,414
	Amygdala subcortical volume	0.0906	34,431	7,066,804
	Brainstem subcortical volume	0.3266	28,809	7,049,062
	Caudate subcortical volume	0.2558	37,741	6,778,918
	Pallidum subcortical volume	0.1672	34,413	7,601,583
	Putamen subcortical volume	0.257	37,571	6,785,508
	Thalamus subcortical volume	0.1786	34,464	7,609,351
Savage et al., 2018	Intelligence	0.197	269,867	9,295,118
Schwartzentruber et al., 2021	Alzheimer's disease or family history of Alzheimer's disease	0.1339	472,868	10,687,077
Yengo et al., 2018	Height	0.483	456,426	2,334,001

Supplementary Table 3

Study	Phenotype	Number of SNPs in DIGPD	Number of SNPs in Iceberg	Number of SNPs in AMP-PD cohorts (PPMI, PDBP, SURE-PD3, LCC)
Adams et al., 2016	Intracranial volume	950,611	991,901	990,690
Chia et al., 2021	Lewy body dementia	873,284	912,900	918,566
Davies et al., 2016	Memory performance	956,779	1,001,468	1,004,156
	Reasoning	956,778	1,001,467	1,004,155
Davies et al., 2018	Reaction time	956,921	1,001,631	1,004,214
Grasby et al., 2020	Banks of the superior temporal sulcus cortical surface area	953,651	995,790	995,837
	Banks of the superior temporal sulcus cortical thickness	953,734	995,835	996,033
	Caudal anterior cingulate cortical surface area	953,886	995,941	996,305
	Caudal anterior cingulate cortical thickness	953,951	995,993	996,431
	Caudal middle frontal cortical surface area	953,873	995,936	996,280
	Caudal middle frontal cortical thickness	953,927	995,978	996,392
	Cuneus cortical surface area	953,884	995,949	996,317
	Cuneus cortical thickness	953,822	995,931	996,179
	Entorhinal cortical surface area	953,499	995,660	995,527
	Entorhinal cortical thickness	953,710	995,806	995,977
	Frontal pole cortical surface area	953,947	995,991	996,422
	Frontal pole cortical thickness	953,953	995,993	996,432
	Whole cortical surface area	953,769	995,825	995,924
	Whole cortical thickness	953,967	995,998	996,449
	Fusiform cortical surface area	953,753	995,858	996,032
	Fusiform cortical thickness	953,893	995,948	996,341
	Inferior parietal cortical surface area	953,776	995,867	996,085
	Inferior parietal cortical thickness	953,860	995,922	996,264
	Inferior temporal cortical surface area	953,866	995,925	996,283
	Inferior temporal cortical thickness	953,874	995,931	996,302
	Insula cortical surface area	953,747	995,860	996,026
	Insula cortical thickness	953,634	995,786	995,806
	Isthmus cingulate cortical surface area	953,780	995,895	996,143
	Isthmus cingulate cortical thickness	953,845	995,948	996,208
	Lateral occipital cortical surface area	953,884	995,944	996,312
	Lateral occipital cortical thickness	953,823	995,933	996,175
	Lateral orbitofrontal cortical surface area	953,931	995,982	996,398
	Lateral orbitofrontal cortical thickness	953,676	995,834	995,897
	Lingual cortical surface area	953,918	995,972	996,382
	Lingual cortical thickness	953,826	995,929	996,177
	Medial orbitofrontal cortical surface area	953,838	995,920	996,207
	Medial orbitofrontal cortical thickness	953,363	995,618	995,220
	Middle temporal cortical surface area	953,642	995,786	995,823
	Middle temporal cortical thickness	953,733	995,837	996,032
	Paracentral cortical surface area	953,861	995,927	996,252
	Paracentral cortical thickness	953,933	995,986	996,394
	Para hippocampal cortical surface area	952,797	995,209	994,150
	Para hippocampal cortical thickness	953,413	995,642	995,240
	Pars opercularis cortical surface area	953,880	995,943	996,300
	Pars opercularis cortical thickness	953,934	995,981	996,401
	Pars orbitalis cortical surface area	953,927	995,981	996,393
	Pars orbitalis cortical thickness	953,938	995,986	996,411
	Pars triangularis cortical surface area	953,891	995,954	996,326
	Pars triangularis cortical thickness	953,911	995,966	996,363
	Pericalcarine cortical surface area	953,890	995,949	996,325
	Pericalcarine cortical thickness	953,543	995,744	995,601
	Postcentral cortical surface area	953,675	995,834	995,876
	Postcentral cortical thickness	953,829	995,906	996,216
	Posterior cingulate cortical surface area	953,934	995,984	996,401
	Posterior cingulate cortical thickness	953,850	995,951	996,215
	Precentral cortical surface area	953,728	995,864	996,004
	Precentral cortical thickness	953,880	995,945	996,299
	Precuneus cortical surface area	953,919	995,972	996,376
	Precuneus cortical thickness	953,945	995,990	996,420
	Rostral anterior cingulate cortical surface area	953,818	995,902	996,151
	Rostral anterior cingulate cortical thickness	953,935	995,986	996,409
	Rostral middle frontal cortical surface area	953,886	995,949	996,321
	Rostral middle frontal cortical thickness	953,789	995,914	996,100
	Superior frontal cortical surface area	953,784	995,874	996,114
	Superior frontal cortical thickness	953,791	995,914	996,106
	Superior parietal cortical surface area	953,762	995,885	996,078
	Superior parietal cortical thickness	953,784	995,913	996,092
	Superior temporal cortical surface area	953,600	995,756	995,747
	Superior temporal cortical thickness	953,650	995,780	995,854
	Supramarginal cortical surface area	953,661	995,792	995,846
	Supramarginal cortical thickness	953,684	995,821	995,863
	Temporal pole cortical surface area	953,843	995,903	996,225
	Temporal pole cortical thickness	953,869	995,930	996,281
	Transverse temporal cortical surface area	953,941	995,988	996,421
	Transverse temporal cortical thickness	953,900	995,956	996,341
Howard et al., 2019	Major depressive disorder	945,679	984,269	982,664
Kunkle et al., 2019	Alzheimer's disease	955,838	1,000,470	1,003,432
Lee et al., 2018	Cognitive performance	954,017	994,264	997,232
	Educational attainment	954,016	994,263	997,232
Malik et al., 2018	Any ischemic stroke	952,723	992,984	997,086
	Any stroke	952,514	992,803	996,946
	Cardioembolic stroke	952,394	992,707	996,794
	Large artery stroke	953,063	993,343	997,218
	Small vessel stroke	952,548	992,886	996,897
Nalls et al., 2019	Parkinson's disease or first degree relation to individual with Parkinson's disease	929,467	969,700	967,088
Neale lab, 2018	Sleeplessness or insomnia	954,454	999,162	1,000,430
	Trouble falling asleep	954,454	999,162	1,000,430
Otowa et al., 2016	Anxiety disorder	895,277	941,142	938,063
Pulit et al., 2019	Body mass index	956,831	1,001,448	1,004,173
Sargurupremraj et al., 2020	White matter hyperintensities	918,495	958,314	947,803
Satizabal et al., 2019	Nucleus accumbens subcortical volume	941,421	983,068	980,663
	Amygdala subcortical volume	941,266	982,846	980,381
	Brainstem subcortical volume	941,721	983,427	980,773
	Caudate subcortical volume	939,604	981,450	977,651
	Pallidum subcortical volume	941,249	982,836	980,361
	Putamen subcortical volume	939,710	981,542	977,832
	Thalamus subcortical volume	941,273	982,855	980,387
Savage et al., 2018	Intelligence	954,824	1,000,307	998,934
Schwartzentruber et al., 2021	Alzheimer's disease or family history of Alzheimer's disease	955,892	1,000,537	1,003,499
Yengo et al., 2018	Height	838,690	870,907	867,632

Supplementary Table 4

		DIGPD				Iceberg				PPMI				PDBP				SURE-PD3				LCC			
Study	Phenotype	Coefficient	Lower bound (95% CI)	Upper bound (95% CI)	p-value	Coefficient	Lower bound (95% CI)	Upper bound (95% CI)	p-value	Coefficient	Lower bound (95% CI)	Upper bound (95% CI)	p-value	Coefficient	Lower bound (95% CI)	Upper bound (95% CI)	p-value	Coefficient	Lower bound (95% CI)	Upper bound (95% CI)	p-value	Coefficient	Lower bound (95% CI)	Upper bound (95% CI)	p-value
Adams et al., 2016	Intracranial volume	-0.0174135639	-0.1998399904	0.1650128626	0.8515909369	-0.07459894222	-0.4366264226	0.2874285381	0.68630994	0.307568477	0.01653330869	0.5986036454	0.03833012955	0.356841759	0.08963049732	0.6240530206	0.008860415566	0.1477043351	-0.1110965924	0.4065052626	0.2633100656	-0.7484686083	-2.389941453	0.8930042363	0.3714865874
Chia et al., 2021	Lewy body dementia	-0.2116862039	-0.4889324086	0.06556000091	0.1345249199	-0.09716481104	-0.5558943665	0.3615647445	0.6780353617	-0.1852249244	-0.5095810686	0.1391312198	0.2630353851	-0.06295825918	-0.3978015685	0.2718850502	0.712486728	-0.05485494857	-0.4022436104	0.2925337132	0.7569479276	-0.2508494122	-2.056524399	1.554825575	0.785403911
Davies et al., 2016	Memory performance	-0.1589302189	-0.3198494762	0.001989038341	0.05289981009	0.09409301746	-0.2033287237	0.3915147587	0.5352191697	-0.302462795	-0.5219868424	-0.08293874752	0.006924340161	-0.02520187605	-0.2482940562	0.1978903041	0.8247734517	-0.1026393143	-0.319564483	0.1142858543	0.3537357394	0.1594342789	-1.30598727	1.624855827	0.8311403271
	Reasoning	0.1651394926	-0.001006189174	0.3312851743	0.05140367908	0.2089577216	-0.08654599586	0.504461439	0.1657660661	0.3713438212	0.1505877849	0.5920998574	0.0009774263099	0.3076944719	0.09070603014	0.5246829137	0.005448074332	0.07762450541	-0.1445876841	0.2998366949	0.4935544133	0.04743491941	-1.227189898	1.322059737	0.9418541134
Davies et al., 2018	Reaction time	-0.06664546645	-0.2292416198	0.09595068687	0.4217685774	-0.349430629	-0.638145651	-0.06071560695	0.01768555467	-0.1467587666	-0.3674566003	0.07393906718	0.1924624492	-0.2068761196	-0.4255411745	0.01178893528	0.06369681945	0.1805506051	-0.03893345172	0.4000346619	0.1068981216	0.2363685061	-1.12421183	1.596948842	0.7334821508
Grasby et al., 2020	Banks of the superior temporal sulcus cortical surface area	-0.1075988436	-0.2891247798	0.07392709269	0.245332325	-0.02355862208	-0.3476713575	0.3005541134	0.8867143562	-0.06373702494	-0.34463864	0.2171645901	0.6565228677	0.07894834725	-0.1777680998	0.3356647943	0.5466748095	-0.1083588526	-0.3691088707	0.1523911656	0.4153617376	0.01431841671	-1.603838644	1.632475478	0.9861630393
	Banks of the superior temporal sulcus cortical thickness	-0.06775307451	-0.2297963547	0.0942902057	0.412504307	-0.07756725145	-0.3772398373	0.2221053344	0.6119323609	0.1145446969	-0.1146582217	0.3437476156	0.3273346653	0.08139217949	-0.1457968812	0.3085812401	0.4825720606	-0.03952447158	-0.2603544659	0.1813055228	0.7257405078	-0.1561092061	-1.450652845	1.138434433	0.8131592737
	Caudal anterior cingulate cortical surface area	0.1240880877	-0.04806032171	0.2962364972	0.1577198175	0.1552925614	-0.1657200292	0.4763051521	0.34305351	-0.04053805443	-0.2996009874	0.2185248785	0.7590760635	-0.1154276858	-0.3552608602	0.1244054886	0.3455285983	-0.1546833737	-0.3911798089	0.08181306164	0.1998642865	0.9241821297	-0.7331732937	2.581537553	0.2744271246
	Caudal anterior cingulate cortical thickness	-0.1443970392	-0.3112910618	0.02249698326	0.08993044509	-0.1269894432	-0.4346771559	0.1806982696	0.4185611819	-0.1680975767	-0.3931414943	0.05694634094	0.1431928547	-0.125512982	-0.3450944777	0.09406851359	0.2625786188	0.01624301745	-0.1957120946	0.2281981295	0.8806065602	-0.9944740834	-2.674342643	0.6853944759	0.2459310704
	Caudal middle frontal cortical surface area	0.111069814	-0.09809371498	0.320233343	0.2979785132	-0.2597727117	-0.6437049643	0.1241595409	0.1847957083	-0.07253037672	-0.4426713389	0.2976105855	0.7009327997	-0.1759403479	-0.5235913354	0.1717106397	0.3212438805	0.3174737803	-0.01571384317	0.6506614038	0.06182790747	-0.3560194099	-2.651747712	1.939708893	0.7611664476
	Caudal middle frontal cortical thickness	0.008426331286	-0.1586554299	0.1755080925	0.9212608198	-0.1328353931	-0.4450729318	0.1794021457	0.4043775828	-0.0496777112	-0.2855991473	0.1862437249	0.6798209954	-0.1056987741	-0.3304868207	0.1190892726	0.3567346434	0.0356938764	-0.1837875516	0.2551753044	0.7499195271	0.3397774927	-1.052981274	1.732536259	0.6325413803
	Cuneus cortical surface area	0.02036094314	-0.1579205205	0.1986424068	0.8228809642	-0.01411716295	-0.3314483076	0.3032139817	0.9305179119	-0.1687749239	-0.4061103387	0.06856049092	0.1633847904	-0.02171617989	-0.2519429185	0.2085105587	0.8533278903	0.08583670002	-0.1442492069	0.3159226069	0.4646622412	0.9619300919	-0.3281286232	2.251988807	0.1438936827
	Cuneus cortical thickness	0.1504171478	-0.02252528961	0.3233595852	0.08825339019	0.1145438629	-0.228286398	0.4573741239	0.5125653687	-0.06305050698	-0.3092404467	0.1831394327	0.6156978848	0.1085720948	-0.1226395314	0.3397837209	0.3573858772	0.09860178682	-0.1350534071	0.3322569807	0.4081810146	0.4861024729	-0.8197345829	1.791939529	0.4656325572
	Entorhinal cortical surface area	-0.01256405901	-0.182420631	0.157292513	0.8847299288	0.4028523798	0.09084611096	0.7148586487	0.01138540022	0.3838503363	0.134618894	0.6330817787	0.002539361521	-0.01932823584	-0.2622201765	0.2235637048	0.8760605945	-0.04015002499	-0.2900370824	0.2097370325	0.7528279539	-0.4451134556	-1.834510814	0.9442839032	0.5300676873
	Entorhinal cortical thickness	0.04148518338	-0.1193675673	0.202337934	0.6132146837	0.01719264696	-0.2767880513	0.3111733452	0.9087438966	-0.1193740284	-0.3404338462	0.1016857894	0.2898750052	0.1639316982	-0.05839355299	0.3862569495	0.1484070574	0.006517204473	-0.2066773617	0.2197117707	0.9522235811	0.7849465208	-0.5447830699	2.114676112	0.2472816474
	Frontal pole cortical surface area	-0.0149084351	-0.1838700194	0.1540531492	0.8626996158	-0.02941257988	-0.3368655935	0.2780404337	0.8512682228	-0.03852426473	-0.2634574501	0.1864089206	0.7371101784	-0.09869181365	-0.3178088821	0.1204252548	0.3773545697	0.1036970919	-0.1200076128	0.3274017966	0.363597964	-0.8138851518	-2.192975405	0.5652051013	0.2473973534
	Frontal pole cortical thickness	0.08481067467	-0.08391550865	0.253536858	0.3245350615	-0.2073974985	-0.5019361225	0.08714112549	0.1675570357	-0.001300534223	-0.2222443038	0.2196432353	0.9907951112	-0.04645439144	-0.2663522584	0.1734434755	0.678836749	0.1968285082	-0.01082427772	0.4044812942	0.06319780321	-0.01092226061	-1.317843205	1.295998684	0.9869313132
	Whole cortical surface area	0.1592198759	-0.02700300821	0.3454427599	0.093784831	-0.06541129265	-0.3962400726	0.2654174873	0.6983690241	0.2598461643	-0.02354924156	0.5432415701	0.07232017158	0.1299786734	-0.1352759085	0.3952332552	0.3368482137	0.3816324461	0.1233285495	0.6399363427	0.003782426821	2.35872169	0.9969998944	3.720443486	0.0006863521573
	Whole cortical thickness	0.0658854874	-0.1068475515	0.2386185263	0.4547085818	-0.1289013595	-0.458703539	0.2009008201	0.4436518516	0.03997774006	-0.1938628124	0.2738182926	0.7375659059	0.06417255632	-0.1651881578	0.2935332704	0.5834337876	0.08234992229	-0.1365223786	0.3012222231	0.4608612278	0.2650273463	-1.090620759	1.620675452	0.7015935184
	Fusiform cortical surface area	-0.1059825258	-0.2692012274	0.05723617583	0.2031386724	0.1760178957	-0.1227403395	0.4747761309	0.2481960204	0.1045435267	-0.1322504384	0.3413374917	0.3868655094	0.05372214561	-0.1808854244	0.2883297157	0.6535710397	-0.1725723404	-0.4013166823	0.05617200151	0.1392306664	-1.164454196	-2.608057929	0.2791495373	0.1138858332
	Fusiform cortical thickness	0.06134552519	-0.1070343443	0.2297253947	0.4751838715	-0.1022149015	-0.4354586837	0.2310288807	0.5477240436	0.01823244238	-0.223059607	0.2595244917	0.8822652299	-0.06276458523	-0.3002171567	0.1746879862	0.6044115671	-0.06855910617	-0.2983917257	0.1612735134	0.558777995	-0.1734457016	-1.661190845	1.314299442	0.8192588293
	Inferior parietal cortical surface area	-0.1122677596	-0.3096629309	0.08512741168	0.2649695154	0.09466141831	-0.2583495453	0.4476723819	0.5991850396	-0.03832264985	-0.3434416371	0.2667963374	0.8055509613	-0.03397079458	-0.3240464934	0.2561049043	0.8184557773	0.007868918504	-0.2935420599	0.3092798969	0.9591911222	-0.2487143817	-2.268414138	1.770985375	0.8092774558
	Inferior parietal cortical thickness	0.09374882322	-0.0703371411	0.2578347875	0.2627967802	0.06882737683	-0.2284047747	0.3660595284	0.6499359026	0.07793758532	-0.1442140278	0.3000891984	0.4916950362	0.1382517549	-0.08888905055	0.3653925603	0.2328875132	0.06336692558	-0.1577342411	0.2844680922	0.5743069763	1.337727858	0.08196689833	2.593488817	0.03680720716
	Inferior temporal cortical surface area	-0.03905804875	-0.2070685434	0.1289524459	0.6486485872	0.2605331667	-0.04726039478	0.5683267281	0.09711179554	-0.008877340439	-0.2365219868	0.218767306	0.9390756496	-0.03804584956	-0.2591080364	0.1830163373	0.7358763731	0.0775718387	-0.1402212251	0.2953649025	0.485124213	0.2253673701	-1.18237246	1.6331072	0.7536928162
	Inferior temporal cortical thickness	0.06860998865	-0.09801112448	0.2352311018	0.4196323614	-0.2994045628	-0.6563671456	0.05755802006	0.1001902799	0.000233535949	-0.2225546524	0.2230217243	0.998360734	0.03030906054	-0.1930290713	0.2536471924	0.7902505059	-0.02715107007	-0.2520363953	0.1977342552	0.8129421023	0.02660926688	-1.406034853	1.459253387	0.9709606543
	Insula cortical surface area	0.1570621794	-0.0407280506	0.3548524093	0.1196184109	-0.1354122517	-0.518545607	0.2477211036	0.4884869261	-0.08147736233	-0.4387019853	0.2757472607	0.6548481998	-0.02489713281	-0.367106219	0.3173119533	0.8866096334	-0.08634859723	-0.4344708832	0.2617736888	0.6268598181	0.8127168799	-1.41358164	3.0390154	0.4743059055
	Insula cortical thickness	0.01042557504	-0.1538417484	0.1746928985	0.9010038918	-0.2553205997	-0.5471032372	0.0364620378	0.08633771426	0.0575361354	-0.1628277651	0.2779000359	0.6088338426	-0.001499064844	-0.2185353632	0.2155372336	0.9891990257	-0.1290797192	-0.3394441307	0.08128469235	0.2291176035	-0.4519993361	-1.825743129	0.921744457	0.5190037634
	Isthmus cingulate cortical surface area	0.1192131702	-0.09894477562	0.337371116	0.2841563212	0.05226850614	-0.3547570724	0.4592940846	0.8012804559	-0.02978766541	-0.40251376	0.3429384292	0.8755308263	0.02918739007	-0.3060146827	0.3643894628	0.8644895975	0.2125004921	-0.1166584129	0.5416593972	0.2057545003	1.175655648	-0.8369889883	3.188300284	0.2522576066
	Isthmus cingulate cortical thickness	-0.1558861233	-0.3221374925	0.01036524595	0.06609682854	-0.2057927982	-0.5168074856	0.1052218892	0.1946749742	0.0354633692	-0.18915263	0.2600793685	0.7569809156	-0.1557899902	-0.3784271153	0.06684713491	0.1702248064	0.1263253592	-0.09242996656	0.345080685	0.2577072312	-1.722675829	-2.917379369	-0.5279722882	0.004711474649
	Lateral occipital cortical surface area	0.04877842982	-0.1337903848	0.2313472445	0.6005151171	-0.1526203199	-0.4770656612	0.1718250213	0.3565418783	-0.02650803866	-0.2830436311	0.2300275537	0.8395065288	0.09397593694	-0.134149813	0.3221016869	0.4194342458	-0.1951346108	-0.4380048608	0.04773563912	0.115317125	0.009871691893	-1.248378812	1.268122196	0.9877313856
	Lateral occipital cortical thickness	0.105445581	-0.06159396849	0.2724851304	0.2159945986	-0.1345618056	-0.4468520487	0.1777284375	0.3983772171	-0.2363469513	-0.4726533255	-0.00004057705249	0.04996067327	0.1461656598	-0.08619701608	0.3785283356	0.2176139011	-0.08330248134	-0.3056738567	0.139068894	0.4628137014	-0.8634201214	-2.246301108	0.5194608649	0.2210542645
	Lateral orbitofrontal cortical surface area	0.01580352331	-0.1656291314	0.197236178	0.8644432698	-0.08828685555	-0.421686274	0.2451125629	0.6037507872	0.1573357798	-0.148260759	0.4629323186	0.3129345324	-0.2720447587	-0.5390686974	-0.005020819981	0.04584507063	0.02641133984	-0.2721923443	0.3250150239	0.8623702011	-0.4670068579	-2.12208901	1.188075294	0.5802401623
	Lateral orbitofrontal cortical thickness	0.03749398573	-0.1281000342	0.2030880057	0.6572036254	-0.2589324058	-0.5686489709	0.05078415932	0.1012988625	-0.02456370586	-0.2708795592	0.2217521474	0.845035766	-0.1374035813	-0.3687721949	0.09396503227	0.2444362649	0.05001826047	-0.1694138393	0.2694503602	0.6550466959	0.2902321603	-1.124490789	1.70495511	0.6876185044
	Lingual cortical surface area	-0.08144625729	-0.2752361634	0.1123436488	0.4100895534	-0.02325340553	-0.405108576	0.3586017649	0.9049951205	-0.2129943556	-0.6027695552	0.1767808441	0.2841555557	-0.0987501691	-0.4499103369	0.2524099987	0.5815213883	0.1955434355	-0.1653703348	0.5564572057	0.2882763572	0.3421750021	-2.007772814	2.692122818	0.77534567
	Lingual cortical thickness	0.09542728865	-0.0682524994	0.2591070767	0.2531703888	0.008708614187	-0.3123973723	0.3298146007	0.9576079595	-0.1979717241	-0.42053189	0.02458844169	0.08125901853	0.2047141307	-0.009185553375	0.4186138147	0.06068320017	0.2711608872	0.05119286191	0.4911289124	0.01568759025	-0.2973375615	-1.792385443	1.19771032	0.6966838266
	Medial orbitofrontal cortical surface area	0.003219417857	-0.1820021727	0.1884410084	0.9728237272	0.1123555672	-0.2124715828	0.4371827173	0.4978106207	0.1097292254	-0.1714329373	0.390891388	0.4443217924	-0.0596845584	-0.3209081364	0.2015390196	0.6542874123	0.05283683537	-0.2150511275	0.3207247982	0.6990722366	-0.3235725819	-1.86084992	1.213704756	0.6799426133
	Medial orbitofrontal cortical thickness	-0.009170859619	-0.1820741927	0.1637324735	0.9172032764	-0.3657953847	-0.6746880695	-0.05690269992	0.02028582028	0.05906779435	-0.196042374	0.3141779627	0.6499678312	0.02828503604	-0.2089990255	0.2655690976	0.8152690399	0.08002514941	-0.1400120494	0.3000623482	0.4759585693	-1.205639435	-2.702564714	0.2912858437	0.1144334118
	Middle temporal cortical surface area	0.0001260481077	-0.1646062856	0.1648583818	0.9988034097	0.1372234099	-0.1717410121	0.4461878319	0.3840282588	-0.2987005107	-0.5232345351	-0.07416648626	0.009124193894	0.0269922648	-0.1958532798	0.2498378094	0.8123453251	-0.03529652805	-0.2464293967	0.1758363406	0.743168292	0.698669589	-0.6239600591	2.021299237	0.3005116198
	Middle temporal cortical thickness	0.06525311779	-0.1048702738	0.2353765094	0.4521890372	0.3580087803	0.02243248367	0.693585077	0.03652959218	-0.1758745663	-0.4182086179	0.06645948527	0.1548959632	-0.1319966645	-0.3572821951	0.09328886613	0.2508203153	-0.08921396342	-0.3218747452	0.1434468183	0.4523218645	0.4632822579	-1.194720281	2.121284796	0.5839275076
	Paracentral cortical surface area	0.06558643732	-0.1153667135	0.2465395882	0.4774631922	-0.02978403118	-0.3663260344	0.3067579721	0.8622920793	0.3063905371	0.01682407417	0.595957	0.03809420356	-0.1351803231	-0.4097637683	0.1394031221	0.3345894315	-0.08044770921	-0.3483048591	0.1874094407	0.5560948295	0.1121322543	-1.752923183	1.977187692	0.9061956895
	Paracentral cortical thickness	-0.04132899801	-0.2115287467	0.1288707506	0.6341234898	0.009233767323	-0.3180191196	0.3364866543	0.9558975956	0.1439439318	-0.09777588605	0.3856637497	0.2431470648	0.0178820739	-0.2090569432	0.244821091	0.8772636538	-0.02686118259	-0.2447985311	0.191076166	0.8091139919	0.8137100904	-0.5301067214	2.157526902	0.2353063196
	Para hippocampal cortical surface area	0.09661720477	-0.07189419542	0.2651286049	0.2611151809	0.07119236292	-0.2349502574	0.3773349832	0.6485463461	0.02614060972	-0.1972613859	0.2495426053	0.818605919	0.02415208363	-0.198696963	0.2470011302	0.8317807157	-0.1629556158	-0.3817607051	0.05584947352	0.144375873	-1.141812004	-2.420661738	0.1370377288	0.08012865356
	Para hippocampal cortical thickness	-0.1226767144	-0.2889152443	0.04356181546	0.1480742618	0.2334641701	-0.06836627508	0.5352946152	0.1295139823	0.2679039615	0.0111030204	0.5247049026	0.04088397053	0.02373388657	-0.2358311169	0.2832988901	0.8577700332	-0.0379539644	-0.2778924154	0.2019844866	0.7565372386	0.6028734826	-0.8844667732	2.090213738	0.4269363048
	Pars opercularis cortical surface area	0.04631822681	-0.1835596204	0.276196074	0.6929061128	-0.06083229582	-0.5489408504	0.4272762587	0.807023569	0.1374217077	-0.3257684673	0.6006118828	0.5609085934	0.1919934108	-0.1890664935	0.5730533151	0.3233930631	0.002845968208	-0.4254222537	0.4311141901	0.989608217	-2.312923073	-5.324770436	0.6989242907	0.1322886007
	Pars opercularis cortical thickness	-0.02074297329	-0.1831135035	0.141627557	0.8022880314	-0.1337183747	-0.4370605974	0.169623848	0.3875960073	-0.05338144612	-0.2753110224	0.1685481301	0.6373290264	0.2177767276	-0.00584139612	0.4413948513	0.05629235906	0.05268734826	-0.168466737	0.2738414335	0.6405440468	-0.02661670435	-1.305488747	1.252255338	0.9674616962
	Pars orbitalis cortical surface area	0.1437235577	-0.02443048775	0.3118776032	0.09389343472	-0.04776289094	-0.362045119	0.2665193372	0.7658065173	-0.1649018087	-0.4146345696	0.08483095232	0.1955998498	-0.09545487901	-0.334493132	0.143583384	0.4338208064	0.01541972194	-0.225352106	0.2561915499	0.9001105123	-0.9457840291	-2.428492192	0.5369241339	0.2112213517
	Pars orbitalis cortical thickness	-0.02946764345	-0.2003735627	0.1414382758	0.7354102839	-0.1006861661	-0.44116197	0.2397896378	0.5621814544	-0.009065008846	-0.2583345453	0.2402045276	0.9431775998	-0.1332826989	-0.3769337065	0.1103683086	0.2836547616	0.03614258379	-0.201889617	0.2741747846	0.7660091815	-0.3556791254	-1.957870013	1.246511763	0.6634875691
	Pars triangularis cortical surface area	-0.1340022489	-0.2993105107	0.03130601304	0.1121085383	0.06876767596	-0.2446584014	0.3821937533	0.6671748579	0.08540646408	-0.1548241888	0.325637117	0.4859256871	0.035105796	-0.205061507	0.275273099	0.7745010494	-0.1112312799	-0.3418505522	0.1193879923	0.3444948841	-0.1317077064	-1.604413608	1.340998195	0.860856174
	Pars triangularis cortical thickness	-0.1193641168	-0.2848526388	0.04612440519	0.1574531179	0.1122374082	-0.1914702824	0.4159450989	0.4688698602	-0.02238556636	-0.2526003714	0.2078292387	0.8488527333	0.1478435569	-0.07712290254	0.3728100164	0.1977285605	0.2354277441	0.01601666031	0.4548388279	0.03546242694	-0.8352961206	-2.32237261	0.6517803693	0.2709320435
	Pericalcarine cortical surface area	-0.002170540735	-0.163305717	0.1589646356	0.9789372397	0.07065604556	-0.2251618407	0.3664739318	0.6396866049	0.0843311145	-0.1428009659	0.3114631949	0.4667920415	-0.05776471112	-0.2832262825	0.1676968603	0.6155581885	0.0919049416	-0.1268489013	0.3106587845	0.4102585748	0.9148794384	-0.257800682	2.087559559	0.1262427039
	Pericalcarine cortical thickness	0.2045042855	0.04268647407	0.3663220969	0.01324957667	0.3583238514	0.04611039713	0.6705373056	0.02448524677	0.04717344167	-0.1801277292	0.2744746126	0.6841803176	0.1499658732	-0.08213127039	0.3820630168	0.205370084	0.005164147175	-0.2139137496	0.224242044	0.9631503199	0.8679357041	-0.4913716811	2.227243089	0.2107656522
	Postcentral cortical surface area	0.1264486929	-0.08360664711	0.3365040328	0.238057813	0.1284703369	-0.2369320623	0.493872736	0.4907630263	-0.01841977029	-0.3361082881	0.2992687475	0.9095232691	0.03998759503	-0.2745254805	0.3545006706	0.8032120122	-0.1013599014	-0.4134442664	0.2107244637	0.5244087046	-0.5660512459	-2.391676286	1.259573794	0.5433836584
	Postcentral cortical thickness	0.00852543089	-0.1573566346	0.1744074963	0.9197636615	0.1928586316	-0.1337715467	0.5194888098	0.2471662358	0.103274133	-0.1256299486	0.3321782146	0.3765491374	0.01030500158	-0.2139491678	0.2345591709	0.9282356323	0.007202150965	-0.2126854661	0.227089768	0.9488139776	0.2210405988	-1.082480794	1.524561992	0.7396213527
	Posterior cingulate cortical surface area	0.05770806568	-0.1216105807	0.2370267121	0.5282021987	-0.05826452319	-0.3984037523	0.2818747059	0.7370713243	0.01015726776	-0.2427982573	0.2631127928	0.9372703676	0.04541442792	-0.1967368359	0.2875656917	0.713184331	-0.03779636365	-0.2748754408	0.1992827135	0.7546852428	1.182933745	-0.1142507356	2.480118225	0.07388282336
	Posterior cingulate cortical thickness	-0.1609084147	-0.3217741945	-0.00004263501026	0.04993931133	-0.1913814914	-0.4995821187	0.116819136	0.2235787035	-0.04822586657	-0.2694843635	0.1730326304	0.6692358611	-0.137897184	-0.358215636	0.08242126806	0.2199202323	-0.05305361775	-0.2675668337	0.1614595982	0.6278606439	-0.922482719	-2.129263485	0.2842980472	0.1340739862
	Precentral cortical surface area	-0.03199406575	-0.224511118	0.1605229865	0.7446340008	0.2562565283	-0.09302586646	0.6055389231	0.1504458475	-0.06151958541	-0.383220509	0.2601813382	0.7078030515	-0.06157669644	-0.3566696151	0.2335162222	0.6825518381	-0.04949129126	-0.3772880812	0.2783054987	0.7672922899	0.084439889	-2.225520338	2.394400116	0.9428837241
	Precentral cortical thickness	-0.001047855379	-0.1694745385	0.1673788277	0.9902710081	0.02893476295	-0.2743582466	0.3322277725	0.8516727818	0.003680749652	-0.2426623309	0.2500238302	0.976637356	-0.1242859094	-0.3484678397	0.09989602095	0.2772139787	0.02471705756	-0.2064569666	0.2558910817	0.834012067	0.5348756793	-1.176973846	2.246725205	0.5402729803
	Precuneus cortical surface area	0.04261424069	-0.1313676315	0.2165961129	0.6311821165	0.08588636783	-0.2411363242	0.4129090598	0.6067293168	0.01426948707	-0.2286250282	0.2571640023	0.9083315244	-0.08837876026	-0.3333930447	0.1566355241	0.4795814727	-0.004814900716	-0.2403404928	0.2307106914	0.9680389388	0.5879810773	-0.849738437	2.025700592	0.422806203
	Precuneus cortical thickness	-0.2166510659	-0.3850670803	-0.0482350515	0.0116920169	0.2863942782	-0.03394521502	0.6067337713	0.07972675525	0.1741501072	-0.06961160709	0.4179118214	0.161437734	0.07650705783	-0.1572936188	0.3103077345	0.5212869371	0.07682397193	-0.1503371563	0.3039851002	0.5074309574	0.3028729378	-0.9716069692	1.577352845	0.6413770271
	Rostral anterior cingulate cortical surface area	0.09211728484	-0.1447104346	0.3289450043	0.4458487493	-0.04916036799	-0.5430436385	0.4447229026	0.8453211223	0.07089868564	-0.4281195622	0.5699169335	0.7806558072	-0.359368572	-0.8012579339	0.08252079	0.11094728	0.07666719423	-0.4009524225	0.554286811	0.7530561451	1.44551472	-1.353113165	4.244142605	0.3113765682
	Rostral anterior cingulate cortical thickness	-0.08497520592	-0.25427936	0.08432894817	0.325251865	-0.1930679708	-0.4959826917	0.1098467501	0.211585655	-0.0913839219	-0.3286488453	0.1458810015	0.4503142841	-0.04108033177	-0.2719769449	0.1898162814	0.7273069945	-0.0247767899	-0.2451735759	0.195618196	0.8256015988	-0.7032358896	-2.164048458	0.7575766791	0.3454110911
	Rostral middle frontal cortical surface area	-0.03796084497	-0.2041926121	0.1282709222	0.6544568843	0.0744856847	-0.2175924453	0.3665638147	0.617195152	-0.1294070621	-0.3465706849	0.08775656077	0.2428326409	-0.0740737314	-0.2978649759	0.1497175131	0.5165078496	0.04842130057	-0.1659642933	0.2628068945	0.6579979176	1.474899957	0.04408690185	2.905713012	0.04334654235
	Rostral middle frontal cortical thickness	0.02507662373	-0.1399119406	0.190065188	0.7657832534	0.005073450174	-0.3122067472	0.3223536475	0.9749978421	0.01803070335	-0.2099503537	0.2460117604	0.8768127922	-0.008920946012	-0.229351993	0.211510101	0.9367775855	0.2057991076	-0.01146387349	0.4230620886	0.06337569378	-0.7682696065	-2.134739001	0.5981997879	0.2704834699
	Superior frontal cortical surface area	0.1276681921	-0.03883884779	0.294175232	0.1328932922	-0.01910988922	-0.3267777751	0.2885579967	0.9031069768	0.1450118172	-0.08002002583	0.3700436601	0.2065848338	0.04943750738	-0.1721743309	0.2710493457	0.6619432793	0.02813673338	-0.1905376778	0.2468111446	0.8008960299	0.8390173156	-0.4951949131	2.173229544	0.2177547702
	Superior frontal cortical thickness	0.01102063276	-0.1518810675	0.173922333	0.8945132386	-0.03145140033	-0.3259234008	0.2630206001	0.8341858941	-0.2109235459	-0.4324287638	0.01058167198	0.06199475544	-0.1887098933	-0.4046599158	0.02724012922	0.08676178666	-0.02346672673	-0.2368715706	0.1899381171	0.8293585333	-0.1573927988	-1.498936758	1.184151161	0.8181328334
	Superior parietal cortical surface area	0.01319943888	-0.1608147496	0.1872136274	0.8818152345	-0.3501132128	-0.6576074761	-0.04261894945	0.02564031676	0.02615496901	-0.2104247438	0.2627346818	0.8284554125	0.007235218216	-0.2139548021	0.2284252386	0.9488816497	0.2801187834	0.06026091978	0.4999766471	0.01251884984	-0.4825806209	-1.830854265	0.8656930236	0.4829786921
	Superior parietal cortical thickness	-0.1721962152	-0.343207796	-0.001184634415	0.0484339489	0.2529019604	-0.04649818836	0.5523021093	0.09780836808	-0.05207006457	-0.2933650481	0.189224919	0.672332534	-0.05740021159	-0.3008116781	0.186011255	0.6439450688	-0.1342111165	-0.3600671568	0.0916449237	0.2441504448	0.100082704	-1.420205507	1.620370915	0.8973361448
	Superior temporal cortical surface area	0.05257437373	-0.1226003768	0.2277491243	0.5563747224	-0.1906157089	-0.5241131543	0.1428817364	0.2626079946	-0.1186887742	-0.4183792792	0.1810017308	0.4376191799	0.08216635317	-0.1922699215	0.3566026278	0.5573285234	-0.09085307614	-0.414372373	0.2326662207	0.5820371295	-0.8877674215	-2.638194918	0.8626600747	0.320204166
	Superior temporal cortical thickness	-0.08782290677	-0.2492775451	0.07363173153	0.2863701968	0.07504470338	-0.2459777906	0.3960671974	0.6468257466	0.1614308822	-0.06439127338	0.3872530378	0.1611851078	-0.1380851997	-0.3611122351	0.08494183565	0.224940923	-0.07202557519	-0.2875969689	0.1435458185	0.5125625242	-0.0818540279	-1.357373869	1.193665813	0.8999085183
	Supramarginal cortical surface area	0.03457447843	-0.1614426176	0.2305915745	0.7295619634	-0.1239589188	-0.4724115328	0.2244936952	0.4856527612	0.02508284442	-0.2886628945	0.3388285834	0.8754875311	0.2405227239	-0.0451200721	0.5261655199	0.09886758719	-0.03321663426	-0.3341340173	0.2677007488	0.8287151161	0.4661874309	-1.461648899	2.394023761	0.6355309219
	Supramarginal cortical thickness	0.06690837477	-0.1045977456	0.2384144951	0.4444944003	-0.1158002878	-0.4367294683	0.2051288926	0.4794358538	0.1223859306	-0.1235959759	0.368367837	0.3294802948	-0.2573884704	-0.4908714079	-0.02390553294	0.03072330537	-0.05833699589	-0.2842582222	0.1675842304	0.6127874502	0.7941862006	-0.7230129785	2.31138538	0.3049133345
	Temporal pole cortical surface area	0.172786967	-0.01322778133	0.3588017154	0.06866949711	0.2612563464	-0.09497620694	0.6174888998	0.1506008951	0.181651282	-0.1126453843	0.4759479482	0.2263688661	-0.01421486242	-0.2994646391	0.2710349142	0.922193579	0.1135738426	-0.1593961795	0.3865438646	0.4147996049	0.131963412	-1.577579637	1.841506461	0.879743835
	Temporal pole cortical thickness	0.01965987318	-0.1464927934	0.1858125398	0.8166070831	0.04504370885	-0.2589199632	0.3490073809	0.771477537	-0.08651539417	-0.319675259	0.1466444706	0.4670687631	0.1135995579	-0.1163915132	0.3435906289	0.3330016251	-0.05512385583	-0.2765160758	0.1662683641	0.6255454619	0.06519195146	-1.294474861	1.424858763	0.9251294902
	Transverse temporal cortical surface area	0.1064446722	-0.06479724963	0.2776865939	0.2231018035	-0.4434171762	-0.7714432292	-0.1153911232	0.008062794221	-0.1090473879	-0.3801234759	0.1620287001	0.4304356667	-0.006799701769	-0.2784155815	0.2648161779	0.9608665057	0.0472200473	-0.2357943257	0.3302344203	0.7436574837	-0.6803746713	-2.542945253	1.18219591	0.474021413
	Transverse temporal cortical thickness	-0.1005292421	-0.2739163551	0.07285787091	0.2557974504	0.1846145693	-0.1533043744	0.522533513	0.2842669505	0.08568507143	-0.1396522502	0.311022393	0.4561017853	-0.1106056551	-0.3401251577	0.1189138475	0.3449099063	-0.07862853985	-0.3026575273	0.1454004476	0.4915169772	0.8651742283	-0.7344976517	2.464846108	0.2891280663
Howard et al., 2019	Major depressive disorder	0.05370686018	-0.1190929755	0.2265066958	0.5424154613	0.1254966432	-0.1664423559	0.4174356423	0.3994884109	-0.2775957884	-0.5031422773	-0.05204929962	0.01585378345	-0.1604818359	-0.382718688	0.06175501622	0.1569715854	-0.0684816892	-0.2894286212	0.1524652428	0.5435300028	-0.1301188154	-1.283656328	1.023418697	0.8250273627
Kunkle et al., 2019	Alzheimer's disease	-0.173996382	-0.4210291222	0.07303635817	0.16743531	-0.4892711419	-0.9479358377	-0.0306064461	0.03655036221	-0.1532700289	-0.5431443075	0.2366042497	0.4409948306	-0.04025407107	-0.4368109636	0.3563028214	0.8422988274	-0.0755274613	-0.437715435	0.2866605124	0.6827499465	-0.05765320593	-2.222489892	2.10718348	0.9583716399
Lee et al., 2018	Cognitive performance	0.1323370594	-0.03547242725	0.3001465461	0.1221874812	0.09112460597	-0.2185409436	0.4007901555	0.5641054266	0.4984580363	0.2768565577	0.720059515	0.00001040246807	0.5028720299	0.2844573829	0.7212866769	0.00000640472235	0.4278832734	0.2060974686	0.6496690783	0.00015601961	0.7948560309	-0.5532398631	2.142951925	0.2478358295
	Educational attainment	0.1541240761	-0.02113607325	0.3293842254	0.08478085689	0.2724100425	-0.02890943703	0.5737295221	0.076407915	0.4927136837	0.2639783347	0.7214490327	0.00002422360574	0.4532139214	0.2243503469	0.6820774958	0.0001039093397	0.22412979	-0.007299276368	0.4555588563	0.05767676112	1.193471431	-0.175120325	2.562063188	0.08741878088
Malik et al., 2018	Any ischemic stroke	-0.05931495729	-0.233669814	0.1150398994	0.5049168881	-0.03694484691	-0.3621817835	0.2882920897	0.8238161383	-0.03255919192	-0.2566407829	0.1915223991	0.7758096593	-0.185271053	-0.4031839336	0.03264182763	0.09563873346	-0.1693972621	-0.385797948	0.04700342377	0.1249684107	-0.06604427498	-1.454423645	1.322335095	0.9257175276
	Any stroke	-0.06694475257	-0.2408433895	0.1069538844	0.4505394415	-0.2041880122	-0.5288168251	0.1204408007	0.2176518289	-0.03776128975	-0.2618535036	0.1863309241	0.7411965237	-0.1681482081	-0.3878518244	0.05155540823	0.1336037829	-0.17097413	-0.3934932074	0.05154494729	0.1320793951	-0.135472974	-1.473953778	1.20300783	0.8427510795
	Cardioembolic stroke	0.138765414	-0.07760757745	0.3551384055	0.2087630686	0.1816865555	-0.1979276064	0.5613007173	0.348215953	0.02024557097	-0.2071938741	0.247685016	0.8614987662	-0.1303508506	-0.3468067824	0.08610508121	0.2378808517	-0.06266150497	-0.2771873022	0.1518642923	0.5669887349	-0.1216509055	-1.447999394	1.204697583	0.8573368504
	Large artery stroke	-0.09729845934	-0.3329906046	0.138393686	0.4184504019	0.02947691494	-0.36327968	0.4222335099	0.8830547045	-0.1420523626	-0.386110606	0.1020058808	0.2539602034	-0.1408951139	-0.3660132304	0.08422300262	0.219940453	-0.08787037342	-0.3084663472	0.1327256004	0.4349696523	-1.179592229	-2.476524942	0.1173404849	0.07464565855
	Small vessel stroke	-0.02637531817	-0.2519703147	0.1992196784	0.8187537789	-0.02204153137	-0.4643226773	0.4202396146	0.9221889373	-0.205339519	-0.4315876317	0.02090859369	0.07526681697	-0.1857509835	-0.4140647616	0.04256279449	0.1108055473	-0.06263280621	-0.2870850152	0.1618194028	0.5844317287	0.2635023374	-1.168573816	1.695578491	0.718373205
Nalls et al., 2019	Parkinson's disease or first degree relation to individual with Parkinson's disease	0.01547284094	-0.1612866327	0.1922323145	0.8637774264	-0.1119527481	-0.4194078061	0.1955023098	0.4754274329	-0.01112348512	-0.2484675967	0.2262206264	0.92681195	0.04842118414	-0.1849738533	0.2818162215	0.6842849438	-0.05381472559	-0.2764579734	0.1688285223	0.6356856081	-0.6229887262	-2.126987751	0.8810102988	0.4168724201
Neale lab, 2018	Sleeplessness or insomnia	0.1620834697	-0.002983344507	0.3271502839	0.0542868739	-0.1675492327	-0.4796893425	0.144590877	0.2927715259	0.2926816912	0.0751571762	0.5102062063	0.008360494746	0.08472283604	-0.1350619806	0.3045076526	0.449931899	-0.07415868112	-0.2934710583	0.1451536961	0.5074934088	-0.461919063	-1.728850882	0.8050127564	0.4748585626
	Trouble falling asleep	0.00034105817	-0.1644812112	0.1651633276	0.9967640669	-0.0928677071	-0.402375668	0.2166402538	0.5564744339	0.08894750534	-0.1296428461	0.3075378568	0.4251391863	-0.2870344542	-0.5065219448	-0.06754696357	0.01037301767	0.05163429225	-0.16439103	0.2676596145	0.6394486176	0.6408749761	-0.7076353842	1.989385336	0.3516124413
Otowa et al., 2016	Anxiety disorder	0.08248757243	-0.08119744488	0.2461725897	0.3232966629	-0.1719776362	-0.4675034929	0.1235482204	0.2540460238	-0.1274965255	-0.3443290138	0.08933596275	0.2491362103	-0.03097665348	-0.2544788272	0.1925255203	0.7858954176	-0.1094406343	-0.3278633544	0.1089820859	0.3260804729	0.959751582	-0.2248593526	2.144362517	0.1123023037
Pulit et al., 2019	Body mass index	0.04548247514	-0.1449836361	0.2359485864	0.6397625387	0.01991408451	-0.3092490705	0.3490772395	0.9056111885	-0.06379610979	-0.3020722095	0.1744799899	0.5997491326	-0.2089770772	-0.4317834043	0.01382924984	0.0660164437	-0.05818274014	-0.2704467434	0.1540812631	0.5911039201	0.8284471831	-0.4287615659	2.085655932	0.1965189047
Sargurupremraj et al., 2020	White matter hyperintensities	0.01912558095	-0.1492033585	0.1874545204	0.8237754978	-0.01222720651	-0.3387123172	0.3142579042	0.9414856794	-0.465667356	-0.7185737854	-0.2127609267	0.0003076048097	-0.0306566186	-0.2794004276	0.2180871904	0.8091232573	0.1452665476	-0.1067963218	0.3973294171	0.2586665799	-0.9596704402	-2.492744239	0.573403359	0.2198623227
Satizabal et al., 2019	Accumbens subcortical volume	-0.04052607255	-0.2020261033	0.1209739582	0.6228435433	0.002219009153	-0.3030624422	0.3075004605	0.9886333592	0.2610560995	0.04082899644	0.4812832027	0.02016135397	0.031898429	-0.1892617596	0.2530586176	0.7774144885	-0.02211243166	-0.2375721494	0.1933472861	0.8405818402	1.371959603	0.02952355757	2.714395649	0.04516993216
	Amygdala subcortical volume	0.006211196067	-0.1564361249	0.168858517	0.9403361694	0.03756384407	-0.2516575862	0.3267852744	0.7990640941	-0.04323761923	-0.2673467285	0.18087149	0.7053280602	0.04449732424	-0.1786701835	0.267664832	0.6959474239	-0.1572067937	-0.3733756157	0.05896202835	0.1540514783	-0.52884896	-1.953888759	0.8961908387	0.4670020275
	Brainstem subcortical volume	0.0710084618	-0.09239031223	0.2344072358	0.3943558461	0.2577859976	-0.04859505685	0.564167052	0.09912830351	-0.03950460753	-0.2621213336	0.1831121185	0.727985293	0.2352159019	0.02156211867	0.4488696851	0.03094609255	0.1573204605	-0.0540976072	0.3687385282	0.1447168656	-0.1646301862	-1.540528648	1.211268275	0.8145849593
	Caudate subcortical volume	-0.1495692001	-0.3129093004	0.01377090026	0.07269783397	-0.4435225234	-0.7574321007	-0.1296129461	0.005618875998	0.028985967	-0.204323179	0.262295113	0.8076158464	0.102690284	-0.1255903883	0.3309709563	0.3779528294	-0.05678922378	-0.2906614107	0.1770829631	0.634130209	0.1184658224	-1.182281028	1.419212673	0.8583270416
	Pallidum subcortical volume	-0.007277867992	-0.1753509677	0.1607952317	0.9323647651	0.01321687989	-0.2906925351	0.3171262949	0.9320722388	0.05265478978	-0.1719280057	0.2772375853	0.6458569399	-0.07287907535	-0.2996803132	0.1539221625	0.5288227242	-0.06312111016	-0.2799747388	0.1537325185	0.5683382862	0.5716829176	-0.7728221523	1.916187988	0.4046328434
	Putamen subcortical volume	-0.07500236193	-0.2472886537	0.09728392981	0.3935250265	-0.1498240016	-0.4673314058	0.1676834026	0.3550391464	0.01720140518	-0.2196899736	0.2540927839	0.8868280711	0.1436707933	-0.08344709111	0.3707886777	0.2150349991	0.1574197182	-0.06664036727	0.3814798038	0.168503637	1.346652806	-0.108926375	2.802231986	0.0697866996
	Thalamus subcortical volume	0.0870608024	-0.07943443027	0.2535560351	0.3054243387	0.3010080587	0.007226645404	0.594789472	0.04462453069	-0.04010984314	-0.2630965253	0.182876839	0.7244261869	0.0675626581	-0.1542106456	0.2893359618	0.5504420118	0.0860284256	-0.1272534885	0.2993103397	0.4291995216	-0.09721461566	-1.514544339	1.320115108	0.893059486
Savage et al., 2018	Intelligence	0.1809827234	-0.001527553636	0.3634930004	0.05194856931	0.1451950669	-0.2126025798	0.5029927135	0.426405477	0.6722484161	0.3789457108	0.9655511214	0.00000704818407	0.7212243794	0.446352764	0.9960959948	0.00000027086221	0.4949030063	0.1866873061	0.8031187064	0.00164886775	2.04551964	0.1706853648	3.920353915	0.03248432666
Schwartzentruber et al., 2021	Alzheimer's disease or family history of Alzheimer's disease	-0.2688213109	-0.5219442304	-0.01569839135	0.03738645529	-0.3190402226	-0.8254133829	0.1873329377	0.2168771837	-0.2735080991	-0.6264775702	0.07946137192	0.1288299733	-0.1376419542	-0.520086355	0.2448024466	0.4805662362	-0.1008893275	-0.4851898325	0.2834111774	0.6068718344	0.6539292385	-1.417639766	2.725498244	0.5361146421
Yengo et al., 2018	Height	-0.05407703791	-0.2419506627	0.1337965869	0.5726515408	0.4446951995	0.0891602392	0.8002301599	0.0142270392	0.06027250734	-0.2059789482	0.3265239629	0.6572698894	-0.150727928	-0.402609721	0.101153865	0.2408531775	0.217663651	-0.01295831794	0.44828562	0.06433713034	0.6603172616	-0.8422277313	2.162862254	0.3890522965

Supplementary Table 5

Study	Phenotype	Meta-analysis (Iceberg, PPMI, PDBP, SURE-PD3, LCC)		
		**Coefficient (95% CI)**	**p-value (coefficient)**	**Heterogeneity p-value**
Adams et al., 2016	Intracranial volume	0.2049 [0.0617 - 0.3481]	0.005051585958	0.2403236785
Chia et al., 2021	Lewy body dementia	-0.1044 [-0.2818 - 0.0731]	0.2489742332	0.982334596
Davies et al., 2016	Memory performance	-0.1060 [-0.2223 - 0.0104]	0.07430713672	0.2444946454
	Reasoning	0.2450 [0.1288 - 0.3612]	0.00003581378235	0.4186213245
Davies et al., 2018	Reaction time	-0.1024 [-0.2181 - 0.0132]	0.08261453478	0.03325684671
Grasby et al., 2020	Banks of the superior temporal sulcus cortical surface area	-0.0271 [-0.1652 - 0.1110]	0.7003066371	0.8947593818
	Banks of the superior temporal sulcus cortical thickness	0.0282 [-0.0908 - 0.1471]	0.6426358223	0.7955366129
	Caudal anterior cingulate cortical surface area	-0.0587 [-0.1875 - 0.0702]	0.3722765219	0.4162985139
	Caudal anterior cingulate cortical thickness	-0.0985 [-0.2150 - 0.0180]	0.09758758755	0.6132333192
	Caudal middle frontal cortical surface area	-0.0303 [-0.2083 - 0.1477]	0.738914315	0.1738961903
	Caudal middle frontal cortical thickness	-0.0495 [-0.1696 - 0.0706]	0.4192033887	0.8454741083
	Cuneus cortical surface area	-0.0204 [-0.1434 - 0.1027]	0.7453200701	0.3367358685
	Cuneus cortical thickness	0.0648 [-0.0616 - 0.1912]	0.3150348049	0.7843850705
	Entorhinal cortical surface area	0.1523 [0.0231 - 0.2816]	0.0209103479	0.03015494441
	Entorhinal cortical thickness	0.0222 [-0.0934 - 0.1378]	0.7068414081	0.3518213451
	Frontal pole cortical surface area	-0.0207 [-0.1388 - 0.0974]	0.7311993159	0.5624383719
	Frontal pole cortical thickness	0.0154 [-0.0989 - 0.1298]	0.7913598044	0.2434521359
	Whole cortical surface area	0.2236 [0.0840 - 0.3632]	0.001690739872	0.006243684389
	Whole cortical thickness	0.0387 [-0.0826 - 0.1601]	0.5317704834	0.8614727026
	Fusiform cortical surface area	0.0143 [-0.1080 - 0.1367]	0.8187337146	0.1403398226
	Fusiform cortical thickness	-0.0489 [-0.1746 - 0.0768]	0.4455420469	0.9769275656
	Inferior parietal cortical surface area	-0.0007 [-0.1552 - 0.1538]	0.9927290679	0.9781554104
	Inferior parietal cortical thickness	0.0997 [-0.0181 - 0.2175]	0.09723373272	0.4024403157
	Inferior temporal cortical surface area	0.0494 [-0.0686 - 0.1673]	0.4119908245	0.5944765175
	Inferior temporal cortical thickness	-0.0331 [-0.1541 - 0.0879]	0.5917785188	0.6369512696
	Insula cortical surface area	-0.0734 [-0.2512 - 0.1044]	0.4184558333	0.9392966243
	Insula cortical thickness	-0.0652 [-0.1794 - 0.0490]	0.2629184845	0.4320031969
	Isthmus cingulate cortical surface area	0.0826 [-0.0952 - 0.2605]	0.3624588092	0.6991281967
	Isthmus cingulate cortical thickness	-0.0436 [-0.1615 - 0.0743]	0.468811266	0.01460031482
	Lateral occipital cortical surface area	-0.0545 [-0.1820 - 0.0731]	0.4026039928	0.5053828612
	Lateral occipital cortical thickness	-0.0748 [-0.1965 - 0.0470]	0.2288718177	0.15470007
	Lateral orbitofrontal cortical surface area	-0.0618 [-0.2103 - 0.0868]	0.4150390367	0.2928328579
	Lateral orbitofrontal cortical thickness	-0.0671 [-0.1894 - 0.0552]	0.2821035857	0.5117340271
	Lingual cortical surface area	-0.0272 [-0.2116 - 0.1572]	0.7727245647	0.6284498221
	Lingual cortical thickness	0.0828 [-0.0343 - 0.2000]	0.1659532352	0.0310349126
	Medial orbitofrontal cortical surface area	0.0426 [-0.0973 - 0.1824]	0.5506249058	0.8766700231
	Medial orbitofrontal cortical thickness	-0.0202 [-0.1445 - 0.1041]	0.7497769967	0.07350669209
	Middle temporal cortical surface area	-0.0590 [-0.1757 - 0.0576]	0.3213793946	0.09885274824
	Middle temporal cortical thickness	-0.0605 [-0.1850 - 0.0641]	0.3415154056	0.1042636673
	Paracentral cortical surface area	0.0106 [-0.1334 - 0.1545]	0.8857582327	0.2309330156
	Paracentral cortical thickness	0.0412 [-0.0805 - 0.1630]	0.5068154377	0.6598497627
	Para hippocampal cortical surface area	-0.0322 [-0.1498 - 0.0853]	0.5909751813	0.2664583081
	Para hippocampal cortical thickness	0.1121 [-0.0183 - 0.2425]	0.09208703428	0.3544467847
	Pars opercularis cortical surface area	0.0689 [-0.1477 - 0.2855]	0.5330912607	0.5168788918
	Pars opercularis cortical thickness	0.0399 [-0.0778 - 0.1575]	0.5067102638	0.355674629
	Pars orbitalis cortical surface area	-0.0809 [-0.2085 - 0.0468]	0.2143820397	0.6602017323
	Pars orbitalis cortical thickness	-0.0463 [-0.1758 - 0.0832]	0.4834677727	0.8652038294
	Pars triangularis cortical surface area	0.0099 [-0.1150 - 0.1348]	0.8761957785	0.7993046216
	Pars triangularis cortical thickness	0.1164 [-0.0025 - 0.2353]	0.05509904497	0.3827443842
	Pericalcarine cortical surface area	0.0540 [-0.0638 - 0.1717]	0.3690706157	0.5232784053
	Pericalcarine cortical thickness	0.1143 [-0.0056 - 0.2342]	0.06170781243	0.297019116
	Postcentral cortical surface area	-0.0007 [-0.1628 - 0.1614]	0.9932585495	0.8572375411
	Postcentral cortical thickness	0.0612 [-0.0586 - 0.1811]	0.3166641718	0.8712688498
	Posterior cingulate cortical surface area	0.0077 [-0.1217 - 0.1371]	0.9070816313	0.4729359153
	Posterior cingulate cortical thickness	-0.1031 [-0.2194 - 0.0132]	0.08219587286	0.6219836207
	Precentral cortical surface area	0.0091 [-0.1513 - 0.1696]	0.9113132067	0.652573334
	Precentral cortical thickness	-0.0219 [-0.1447 - 0.1009]	0.726574665	0.8243029189
	Precuneus cortical surface area	-0.0037 [-0.1313 - 0.1238]	0.9542339319	0.8409119318
	Precuneus cortical thickness	0.1356 [0.0115 - 0.2598]	0.0322949272	0.8237944569
	Rostral anterior cingulate cortical surface area	-0.0697 [-0.3070 - 0.1677]	0.5650826724	0.4856586596
	Rostral anterior cingulate cortical thickness	-0.0780 [-0.1988 - 0.0429]	0.2061428063	0.8101414178
	Rostral middle frontal cortical surface area	-0.0210 [-0.1363 - 0.0944]	0.7215915085	0.185710249
	Rostral middle frontal cortical thickness	0.0582 [-0.0601 - 0.1765]	0.3345902381	0.4392714927
	Superior frontal cortical surface area	0.0655 [-0.0522 - 0.1833]	0.2752732942	0.7001979983
	Superior frontal cortical thickness	-0.1227 [-0.2375 - -0.0079]	0.03616237749	0.7044576006
	Superior parietal cortical surface area	0.0348 [-0.0846 - 0.1542]	0.5676195254	0.02211024357
	Superior parietal cortical thickness	-0.0250 [-0.1488 - 0.0988]	0.692545741	0.3606811888
	Superior temporal cortical surface area	-0.0717 [-0.2237 - 0.0803]	0.3553985447	0.6199651524
	Superior temporal cortical thickness	-0.0067 [-0.1250 - 0.1115]	0.9109796652	0.3955899799
	Supramarginal cortical surface area	0.0462 [-0.1081 - 0.2006]	0.5571475039	0.5312226814
	Supramarginal cortical thickness	-0.0715 [-0.1959 - 0.0529]	0.2598202818	0.1876238197
	Temporal pole cortical surface area	0.1221 [-0.0262 - 0.2703]	0.1066954503	0.8043604265
	Temporal pole cortical thickness	-0.0007 [-0.1210 - 0.1197]	0.9914046764	0.7729737233
	Transverse temporal cortical surface area	-0.1077 [-0.2503 - 0.0348]	0.1384653668	0.1941183656
	Transverse temporal cortical thickness	-0.0004 [-0.1218 - 0.1211]	0.9954524115	0.3807780243
Howard et al., 2019	Major depressive disorder	-0.1199 [-0.2370 - -0.0027]	0.04492611865	0.2947076891
Kunkle et al., 2019	Alzheimer's disease	-0.1637 [-0.3616 - 0.0343]	0.1050677432	0.6361680374
Lee et al., 2018	Cognitive performance	0.4237 [0.3064 - 0.5411]	1,46092E-12	0.2255709518
	Educational attainment	0.3784 [0.2575 - 0.4993]	.000000000851656502	0.2961590315
Malik et al., 2018	Any ischemic stroke	-0.1183 [-0.2359 - -0.0007]	0.04872338014	0.8467579094
	Any stroke	-0.1369 [-0.2557 - -0.0181]	0.02390442983	0.8966952938
	Cardioembolic stroke	-0.0366 [-0.1562 - 0.0830]	0.5483422939	0.6801751652
	Large artery stroke	-0.1166 [-0.2415 - 0.0082]	0.06714713677	0.5147086699
	Small vessel stroke	-0.1372 [-0.2620 - -0.0123]	0.03126482571	0.8253461002
Nalls et al., 2019	Parkinson's disease or first degree relation to individual with Parkinson's disease	-0.0275 [-0.1494 - 0.0943]	0.6578256616	0.8493087187
Neale lab, 2018	Sleeplessness or insomnia	0.0597 [-0.0569 - 0.1764]	0.3155845951	0.07264745101
	Trouble falling asleep	-0.0487 [-0.1648 - 0.0675]	0.4114749451	0.09290005254
Otowa et al., 2016	Anxiety disorder	-0.0929 [-0.2088 - 0.0230]	0.1163538897	0.444783486
Pulit et al., 2019	Body mass index	-0.0847 [-0.2044 - 0.0349]	0.1651702025	0.4482328099
Sargurupremraj et al., 2020	White matter hyperintensities	-0.1049 [-0.2369 - 0.0272]	0.1196590648	0.009287680842
Satizabal et al., 2019	Accumbens subcortical volume	0.0859 [-0.0305 - 0.2022]	0.1480647067	0.1144450243
	Amygdala subcortical volume	-0.0426 [-0.1589 - 0.0738]	0.4734357913	0.6613096303
	Brainstem subcortical volume	0.1393 [0.0243 - 0.2543]	0.01759316166	0.4003128806
	Caudate subcortical volume	-0.0445 [-0.1671 - 0.0780]	0.4761796529	0.08263645569
	Pallidum subcortical volume	-0.0175 [-0.1354 - 0.1005]	0.7716327331	0.8176011843
	Putamen subcortical volume	0.0796 [-0.0421 - 0.2013]	0.1996529875	0.2027507687
	Thalamus subcortical volume	0.0791 [-0.0367 - 0.1950]	0.1805499427	0.4989251619
Savage et al., 2018	Intelligence	0.5585 [0.4069 - 0.7102]	5,25612E-13	0.04714298995
Schwartzentruber et al., 2021	Alzheimer's disease or family history of Alzheimer's disease	-0.1906 [-0.3876 - 0.0064]	0.05791699694	0.8475713161
Yengo et al., 2018	Height	0.1118 [-0.0206 - 0.2443]	0.09787226594	0.06094669228

Supplementary Table 6

		PGS			PGS x Number of mild GBA mutations			PGS x Number of undetermined GBA mutations			PGS x rs429358 (APOE)			PGS x rs7412		
Study	Phenotype	Coefficient (95% CI)	p-value (coefficient)	Heterogeneity p-value	Coefficient (95% CI)	p-value (coefficient)	Heterogeneity p-value	Coefficient (95% CI)	p-value (coefficient)	Heterogeneity p-value	Coefficient (95% CI)	p-value (coefficient)	Heterogeneity p-value	Coefficient (95% CI)	p-value (coefficient)	Heterogeneity p-value
Adams et al., 2016	Intracranial volume	0.1884 [-0.0365 - 0.4132]	0.100586057	0.2524621938	-0.3114 [-0.7090 - 0.0861]	0.1247087778	0.2065961033	0.0068 [-0.0148 - 0.0285]	0.5362380676	0.929688257	0.1030 [-0.1391 - 0.3451]	0.4043050196	0.2539519707	0.1021 [-0.2322 - 0.4363]	0.5495936339	0.2113049998
Chia et al., 2021	Lewy body dementia	-0.1463 [-0.3941 - 0.1015]	0.2472370517	0.0823478724	0.0280 [-0.4174 - 0.4733]	0.9019708177	0.1317095553	0.0037 [-0.0183 - 0.0257]	0.7395169045	0.1181131667	0.0398 [-0.2011 - 0.2807]	0.7462343668	0.09414286361	0.0467 [-0.3712 - 0.4645]	0.8267752423	0.5331922426
Davies et al., 2016	Memory performance	-0.2298 [-0.4291 - -0.0305]	0.02385613191	0.02277779883	-0.0524 [-0.4724 - 0.3676]	0.8067418607	0.5225790148	0.0077 [-0.0139 - 0.0293]	0.4838557916	0.02107950795	0.0559 [-0.1888 - 0.3007]	0.654078025	0.811310367	0.1330 [-0.1811 - 0.4470]	0.4066145319	0.1102384291
	Reasoning	0.3240 [0.1201 - 0.5280]	0.001847513278	0.8969299416	-0.3026 [-0.7654 - 0.1603]	0.2001268645	0.8781556367	-0.0006 [-0.0226 - 0.0213]	0.9548173466	0.1246330317	0.0300 [-0.2076 - 0.2677]	0.80441876	0.02381491841	-0.1058 [-0.3700 - 0.1585]	0.4327269578	0.8661820676
Davies et al., 2018	Reaction time	-0.0727 [-0.2692 - 0.1239]	0.4686805884	0.03742714115	-0.2143 [-0.6758 - 0.2472]	0.3627097981	0.3155151586	0.0126 [-0.0093 - 0.0345]	0.2583282252	0.1438991555	-0.0798 [-0.3207 - 0.1611]	0.5160461746	0.06243976669	0.0912 [-0.2189 - 0.4014]	0.5642436166	0.2368561332
Grasby et al., 2020	Banks of the superior temporal sulcus cortical surface area	0.0246 [-0.2016 - 0.2508]	0.8311979892	0.4429572132	0.3663 [-0.0548 - 0.7874]	0.08824563851	0.02521359643	-0.0092 [-0.0309 - 0.0125]	0.4069643154	0.4000126442	-0.0761 [-0.3270 - 0.1747]	0.5519316408	0.1605944703	-0.1295 [-0.4679 - 0.2089]	0.4530880433	0.3459819841
	Banks of the superior temporal sulcus cortical thickness	0.0739 [-0.1248 - 0.2726]	0.4659609463	0.7501263882	0.1962 [-0.1834 - 0.5758]	0.3111531886	0.6516515348	-0.0115 [-0.0318 - 0.0088]	0.2685300946	0.4154706878	0.2507 [0.0232 - 0.4782]	0.0307908199	0.008100622953	-0.1196 [-0.4650 - 0.2257]	0.4971473226	0.2803657073
	Caudal anterior cingulate cortical surface area	0.0687 [-0.1453 - 0.2826]	0.5293099153	0.7553508692	-0.5767 [-1.0264 - -0.1271]	0.0119442932	0.6021615443	-0.0085 [-0.0307 - 0.0137]	0.4535797691	0.5057973888	-0.0911 [-0.3238 - 0.1416]	0.4428429021	0.0006356846212	-0.0791 [-0.4433 - 0.2851]	0.670342666	0.6290284727
	Caudal anterior cingulate cortical thickness	-0.0517 [-0.2537 - 0.1502]	0.6155994517	0.6851251622	0.3797 [-0.0847 - 0.8441]	0.1090587684	0.5501208966	-0.0121 [-0.0326 - 0.0085]	0.2489781035	0.3587595905	0.0041 [-0.2444 - 0.2527]	0.9739887619	0.3965890111	-0.0587 [-0.3795 - 0.2620]	0.7196949207	0.6176869394
	Caudal middle frontal cortical surface area	0.0052 [-0.2657 - 0.2760]	0.9701365814	0.3769338319	-0.1136 [-0.5016 - 0.2743]	0.565892189	0.1920511819	0.0045 [-0.0176 - 0.0266]	0.6905434424	0.1188053096	-0.0525 [-0.2830 - 0.1780]	0.65530876	0.03843938836	0.1130 [-0.2074 - 0.4335]	0.4893385394	0.3445462979
	Caudal middle frontal cortical thickness	0.0691 [-0.1390 - 0.2772]	0.5154572214	0.4193946825	-0.1226 [-0.5432 - 0.2980]	0.5678077715	0.02616113369	-0.0101 [-0.0320 - 0.0117]	0.3635380848	0.9559194848	-0.1131 [-0.3554 - 0.1293]	0.3605016659	0.7090783774	-0.0213 [-0.3486 - 0.3061]	0.8987110503	0.2491126961
	Cuneus cortical surface area	0.0882 [-0.1127 - 0.2891]	0.3896459877	0.7500727658	-0.4508 [-0.9123 - 0.0108]	0.05559965221	0.6992250304	-0.0202 [-0.0417 - 0.0013]	0.06592696974	0.8540184847	0.0816 [-0.1531 - 0.3162]	0.4957293893	0.09458260293	0.0972 [-0.2008 - 0.3952]	0.5225605944	0.5911860498
	Cuneus cortical thickness	0.0881 [-0.1209 - 0.2971]	0.4084854567	0.8738076908	-0.2480 [-0.6980 - 0.2021]	0.2802051512	0.4007265259	0.0053 [-0.0161 - 0.0267]	0.6303126658	0.855848152	-0.1735 [-0.4112 - 0.0643]	0.1527772431	0.177409451	-0.0339 [-0.3807 - 0.3129]	0.8481190055	0.5330215102
	Entorhinal cortical surface area	0.0739 [-0.1357 - 0.2836]	0.4894870289	0.1350731723	0.2143 [-0.1962 - 0.6247]	0.3062915085	0.8308145352	0.0005 [-0.0206 - 0.0215]	0.9642075659	0.3415538806	0.1156 [-0.1351 - 0.3662]	0.366190096	0.5816375221	-0.0538 [-0.4105 - 0.3029]	0.767657094	0.1077004232
	Entorhinal cortical thickness	0.0471 [-0.1521 - 0.2464]	0.6430116836	0.5554637705	0.4707 [0.0501 - 0.8913]	0.02827051719	0.6255854843	-0.0145 [-0.0367 - 0.0077]	0.1989775423	0.1579313692	0.0043 [-0.2198 - 0.2285]	0.9698803144	0.2447472027	0.0488 [-0.2906 - 0.3882]	0.7781591103	0.4788717388
	Frontal pole cortical surface area	-0.1222 [-0.3212 - 0.0767]	0.228459071	0.6420908145	0.2632 [-0.1927 - 0.7191]	0.2577946059	0.0006215509816	0.0180 [-0.0035 - 0.0395]	0.1014511283	0.1215441082	-0.0621 [-0.3007 - 0.1766]	0.6103335259	0.3830225273	-0.0061 [-0.3331 - 0.3209]	0.9707908478	0.5104665148
	Frontal pole cortical thickness	0.0858 [-0.1132 - 0.2848]	0.397869961	0.5712174037	0.2293 [-0.2685 - 0.7270]	0.3665973903	0.02196087946	-0.0086 [-0.0300 - 0.0128]	0.4321228597	0.6010476092	-0.1269 [-0.3737 - 0.1198]	0.3133235808	0.9698847082	0.0917 [-0.2048 - 0.3883]	0.5444294606	0.410191925
	Whole cortical surface area	0.3472 [0.1195 - 0.5749]	0.002801986149	0.02835315072	-0.6028 [-1.0091 - -0.1964]	0.003647860062	0.5276079532	-0.0012 [-0.0239 - 0.0215]	0.9172908445	0.918062439	-0.0553 [-0.3002 - 0.1895]	0.6578393202	0.756838172	0.0458 [-0.2873 - 0.3788]	0.7876169418	0.007780183805
	Whole cortical thickness	0.0892 [-0.1113 - 0.2897]	0.3833801124	0.7751370482	0.1452 [-0.2697 - 0.5602]	0.4926779977	0.8311219176	-0.0119 [-0.0340 - 0.0101]	0.288889594	0.495912177	-0.0298 [-0.2653 - 0.2056]	0.8039391498	0.6738372907	0.1819 [-0.2149 - 0.5787]	0.3689058745	0.9526633945
	Fusiform cortical surface area	0.2321 [0.0219 - 0.4423]	0.0304619194	0.3149048347	-0.6833 [-1.1713 - -0.1953]	0.006066667458	0.326708888	-0.0214 [-0.0431 - 0.0002]	0.05268226182	0.2640172424	-0.1273 [-0.3749 - 0.1203]	0.31362658	0.6309265929	-0.2047 [-0.5596 - 0.1503]	0.2584566015	0.6562602937
	Fusiform cortical thickness	0.0365 [-0.1704 - 0.2434]	0.729590052	0.5024638237	-0.0236 [-0.4000 - 0.3528]	0.9022131142	0.1155705233	-0.0172 [-0.0397 - 0.0052]	0.1328849531	0.2080227343	-0.0128 [-0.2464 - 0.2209]	0.9147165325	0.9639059073	0.1730 [-0.1531 - 0.4992]	0.2985023824	0.4148735558
	Inferior parietal cortical surface area	-0.0003 [-0.2446 - 0.2440]	0.9982002912	0.4659811296	0.2590 [-0.1060 - 0.6241]	0.1643244964	0.2617891333	0.0022 [-0.0203 - 0.0247]	0.8476345389	0.01087736318	-0.0529 [-0.3009 - 0.1952]	0.6761011855	0.0008948057938	-0.1758 [-0.5026 - 0.1511]	0.2919447425	0.1855254453
	Inferior parietal cortical thickness	0.0853 [-0.1170 - 0.2876]	0.4085910456	0.480233798	0.0002 [-0.4390 - 0.4395]	0.9992704247	0.2023334587	0.0006 [-0.0212 - 0.0223]	0.9599346228	0.3335511658	0.1862 [-0.0644 - 0.4367]	0.1452848232	0.05977536263	-0.0039 [-0.3355 - 0.3278]	0.9818164846	0.8992675032
	Inferior temporal cortical surface area	0.0174 [-0.1842 - 0.2190]	0.865514901	0.9694185095	0.9317 [0.4693 - 1.3940]	0.00007824617559	0.4032571809	-0.0098 [-0.0299 - 0.0103]	0.3403575922	0.8050862429	0.0584 [-0.1763 - 0.2931]	0.6256819928	0.7817653515	-0.2586 [-0.6020 - 0.0848]	0.1399798469	0.6142223832
	Inferior temporal cortical thickness	0.0269 [-0.1769 - 0.2308]	0.7958157825	0.5900223872	0.0600 [-0.3746 - 0.4946]	0.7865693732	0.1132970626	-0.0096 [-0.0307 - 0.0116]	0.3761251379	0.9472909058	-0.0039 [-0.2433 - 0.2355]	0.974564748	0.07522494134	0.1025 [-0.2235 - 0.4285]	0.5376543814	0.1096282325
	Insula cortical surface area	0.0389 [-0.2202 - 0.2980]	0.7682975574	0.5959619091	-0.4157 [-0.7841 - -0.0472]	0.02701546919	0.8383848311	-0.0091 [-0.0316 - 0.0134]	0.4285169753	0.02277290583	-0.0001 [-0.2589 - 0.2588]	0.999639076	0.00516861176	0.0013 [-0.3303 - 0.3330]	0.9936521457	0.0183175128
	Insula cortical thickness	0.0389 [-0.1589 - 0.2368]	0.6996754829	0.3500007786	0.4299 [-0.0861 - 0.9458]	0.1024710573	0.7363617557	-0.0058 [-0.0262 - 0.0146]	0.5782389565	0.5573942281	-0.0557 [-0.3032 - 0.1919]	0.6593086771	0.5010973749	-0.1849 [-0.5178 - 0.1481]	0.2765500132	0.5988717792
	Isthmus cingulate cortical surface area	-0.1103 [-0.3694 - 0.1489]	0.4042470244	0.2806244599	0.2605 [-0.1163 - 0.6374]	0.1754017957	0.3249326776	0.0173 [-0.0052 - 0.0398]	0.1327234146	0.5858070052	0.0608 [-0.2026 - 0.3243]	0.6509271364	0.1274301584	0.1916 [-0.1136 - 0.4968]	0.2184559616	0.07695988696
	Isthmus cingulate cortical thickness	0.0116 [-0.1873 - 0.2105]	0.9091364107	0.2991521668	-0.0667 [-0.4858 - 0.3525]	0.7551638959	0.7059535239	-0.0132 [-0.0342 - 0.0079]	0.2197642748	0.8902343892	0.0649 [-0.1733 - 0.3031]	0.5933678182	0.2768496447	0.1375 [-0.2032 - 0.4783]	0.4289644629	0.536810944
	Lateral occipital cortical surface area	0.0158 [-0.1951 - 0.2267]	0.8831931736	0.2032211054	-0.3552 [-0.7530 - 0.0427]	0.08014505506	0.09904302996	-0.0031 [-0.0248 - 0.0185]	0.7765499757	0.3169797414	-0.0573 [-0.2944 - 0.1798]	0.6356035236	0.09164550629	-0.0239 [-0.3470 - 0.2992]	0.8847021457	0.5152852696
	Lateral occipital cortical thickness	-0.1630 [-0.3687 - 0.0427]	0.1204129148	0.5258288938	-0.1496 [-0.5895 - 0.2904]	0.5051781956	0.8151211456	0.0116 [-0.0099 - 0.0330]	0.2898636851	0.2422671854	-0.0010 [-0.2547 - 0.2527]	0.9940055268	0.7118338316	0.1903 [-0.1241 - 0.5046]	0.2355089188	0.4813864366
	Lateral orbitofrontal cortical surface area	0.0295 [-0.2038 - 0.2629]	0.8040069274	0.01948472976	-0.2994 [-0.6981 - 0.0992]	0.1409747059	0.1551633269	-0.0109 [-0.0333 - 0.0115]	0.3388606927	0.1341146417	0.0125 [-0.2327 - 0.2578]	0.9202092174	0.03454369991	-0.1348 [-0.4626 - 0.1930]	0.4203107629	0.240543787
	Lateral orbitofrontal cortical thickness	-0.0558 [-0.2647 - 0.1531]	0.6008677395	0.5985746033	0.5389 [0.0946 - 0.9832]	0.01743150559	0.1677178539	-0.0036 [-0.0247 - 0.0174]	0.7368886817	0.05408853408	-0.0834 [-0.3338 - 0.1670]	0.51381369	0.2774543991	0.2075 [-0.1370 - 0.5520]	0.2377424254	0.3800534209
	Lingual cortical surface area	-0.1063 [-0.3770 - 0.1644]	0.4414383154	0.3210699532	0.2464 [-0.1212 - 0.6140]	0.1889380246	0.118310523	0.0068 [-0.0163 - 0.0299]	0.565711795	0.06725519861	0.1377 [-0.1165 - 0.3920]	0.2882762783	0.194648979	-0.0270 [-0.3775 - 0.3236]	0.880114903	0.2992664383
	Lingual cortical thickness	0.0647 [-0.1312 - 0.2606]	0.5174933038	0.07140507394	0.1252 [-0.3064 - 0.5567]	0.5697704103	0.5720919576	0.0036 [-0.0179 - 0.0252]	0.7403810988	0.1141713904	-0.1362 [-0.3975 - 0.1251]	0.307012785	0.3229178584	0.1650 [-0.1376 - 0.4675]	0.2851832474	0.684632199
	Medial orbitofrontal cortical surface area	0.1266 [-0.1024 - 0.3556]	0.2786405741	0.8309760145	-0.2547 [-0.6123 - 0.1029]	0.162667664	0.3189438707	-0.0106 [-0.0337 - 0.0125]	0.3696486761	0.6229072262	-0.0574 [-0.3174 - 0.2026]	0.6652346663	0.7831352696	-0.0617 [-0.3921 - 0.2687]	0.7144988257	0.6964453818
	Medial orbitofrontal cortical thickness	-0.0893 [-0.2968 - 0.1183]	0.3991871613	0.7991195506	0.4643 [0.0382 - 0.8904]	0.03268683701	0.8773265742	0.0075 [-0.0144 - 0.0294]	0.5002962828	0.7467446626	0.0646 [-0.1791 - 0.3083]	0.6032102307	0.7678104225	0.1899 [-0.1611 - 0.5409]	0.2889442337	0.2093374749
	Middle temporal cortical surface area	-0.0498 [-0.2580 - 0.1585]	0.6394393767	0.1040329701	0.2797 [-0.1607 - 0.7201]	0.2132099951	0.3739711722	-0.0069 [-0.0278 - 0.0139]	0.5140711581	0.3937080286	-0.0389 [-0.2843 - 0.2064]	0.7556610551	0.9974775718	-0.1232 [-0.4673 - 0.2209]	0.4829261875	0.6171022271
	Middle temporal cortical thickness	-0.1619 [-0.3760 - 0.0521]	0.1381881359	0.7265546515	0.0184 [-0.4284 - 0.4653]	0.9355786733	0.3495362366	-0.0093 [-0.0309 - 0.0123]	0.3970924637	0.6656309032	0.2174 [-0.0033 - 0.4382]	0.05350130181	0.1857091027	0.2307 [-0.1061 - 0.5675]	0.1795008212	0.6955097132
	Paracentral cortical surface area	0.1531 [-0.0802 - 0.3864]	0.1984640576	0.1579535454	-0.4507 [-0.8240 - -0.0774]	0.01795663782	0.4668360037	-0.0086 [-0.0301 - 0.0129]	0.4323625033	0.02773899511	-0.1537 [-0.4117 - 0.1043]	0.2430224716	0.0002453615741	0.1242 [-0.2408 - 0.4893]	0.5047670313	0.2916704943
	Paracentral cortical thickness	0.0164 [-0.1851 - 0.2179]	0.8732337472	0.3180213612	-0.1719 [-0.6136 - 0.2697]	0.4454494465	0.4077333628	0.0169 [-0.0046 - 0.0383]	0.1236091336	0.4127991778	-0.1056 [-0.3500 - 0.1389]	0.397356049	0.5129599769	-0.0594 [-0.3836 - 0.2647]	0.7193038546	0.2471381039
	Para hippocampal cortical surface area	0.0880 [-0.1171 - 0.2930]	0.4005205835	0.6935490099	-0.7299 [-1.1833 - -0.2764]	0.001605748888	0.8240316119	-0.0053 [-0.0279 - 0.0173]	0.6466803494	0.9188580872	0.0352 [-0.1992 - 0.2696]	0.7685689617	0.66302676	-0.3492 [-0.6783 - -0.0201]	0.03757304285	0.9637610757
	Para hippocampal cortical thickness	0.0110 [-0.2163 - 0.2384]	0.9243309369	0.6781794797	0.6780 [0.2787 - 1.0773]	0.0008746466536	0.8526006856	-0.0026 [-0.0252 - 0.0199]	0.8187117419	0.900246773	0.1050 [-0.1442 - 0.3543]	0.4086877472	0.4527923508	-0.0339 [-0.3617 - 0.2940]	0.8394576791	0.03964580571
	Pars opercularis cortical surface area	0.0847 [-0.2094 - 0.3787]	0.5724681503	0.4381320531	-0.1802 [-0.5397 - 0.1793]	0.3258221364	0.03633098225	-0.0027 [-0.0259 - 0.0205]	0.8195177431	0.4674249021	-0.0581 [-0.3191 - 0.2028]	0.6624254893	0.04673355247	0.2205 [-0.1066 - 0.5476]	0.1864727548	0.2574694838
	Pars opercularis cortical thickness	0.1708 [-0.0263 - 0.3679]	0.08939138611	0.9757698296	-0.1448 [-0.5495 - 0.2598]	0.4830003511	0.5300166638	-0.0071 [-0.0286 - 0.0144]	0.5195029478	0.1272450972	-0.1084 [-0.3541 - 0.1373]	0.3873712371	0.06973658121	-0.0816 [-0.4201 - 0.2570]	0.6367780422	0.9277084292
	Pars orbitalis cortical surface area	0.0341 [-0.1792 - 0.2473]	0.7541735609	0.3781241618	-0.4873 [-0.8905 - -0.0840]	0.01788133234	0.2669158065	-0.0103 [-0.0327 - 0.0121]	0.3682252076	0.2023784556	-0.0452 [-0.2851 - 0.1948]	0.7121952068	0.04669807358	-0.0331 [-0.3562 - 0.2900]	0.8407898876	0.5696175288
	Pars orbitalis cortical thickness	-0.0562 [-0.2718 - 0.1594]	0.6095986497	0.6360395944	0.3019 [-0.1324 - 0.7361]	0.1730301178	0.694548129	0.0051 [-0.0168 - 0.0269]	0.6490907643	0.3110844285	-0.1121 [-0.3688 - 0.1446]	0.392026707	0.4012660157	-0.1150 [-0.4355 - 0.2055]	0.48180527	0.09811864483
	Pars triangularis cortical surface area	0.0969 [-0.1021 - 0.2959]	0.3401163653	0.322749323	-0.2208 [-0.5964 - 0.1549]	0.249362415	0.1291222961	-0.0088 [-0.0311 - 0.0136]	0.4427055005	0.2027555799	-0.1372 [-0.3797 - 0.1053]	0.2675038293	0.6985096437	0.0376 [-0.3013 - 0.3764]	0.8280370848	0.02989377165
	Pars triangularis cortical thickness	0.1955 [-0.0130 - 0.4039]	0.06608975667	0.6581752391	0.1169 [-0.3114 - 0.5452]	0.5926468747	0.6653806095	-0.0055 [-0.0266 - 0.0155]	0.6055578932	0.6845229747	-0.0655 [-0.2915 - 0.1606]	0.5703403874	0.09098012142	-0.0444 [-0.3888 - 0.3000]	0.8006152017	0.7711258906
	Pericalcarine cortical surface area	0.1125 [-0.0853 - 0.3104]	0.2649784233	0.7505463168	0.0267 [-0.3814 - 0.4348]	0.8979930663	0.5687113946	-0.0242 [-0.0457 - -0.0028]	0.02693924029	0.7012271511	0.1141 [-0.1164 - 0.3445]	0.3320906214	0.682037139	0.2132 [-0.1356 - 0.5619]	0.230990037	0.59129884
	Pericalcarine cortical thickness	0.1352 [-0.0640 - 0.3344]	0.1835341314	0.4167145121	0.0710 [-0.3194 - 0.4615]	0.7213503222	0.8000623026	-0.0022 [-0.0240 - 0.0195]	0.8405080149	0.2067330186	-0.2702 [-0.5186 - -0.0218]	0.03302261671	0.1593553236	0.0935 [-0.2285 - 0.4154]	0.5693217752	0.3815128139
	Postcentral cortical surface area	-0.0889 [-0.3290 - 0.1512]	0.4680408148	0.2584306538	0.2028 [-0.1944 - 0.6001]	0.3169414518	0.7052396408	-0.0020 [-0.0245 - 0.0206]	0.8651170756	0.7194258996	0.1142 [-0.1332 - 0.3615]	0.3656438008	0.4592958205	-0.0644 [-0.3699 - 0.2410]	0.6792097907	0.04358217074
	Postcentral cortical thickness	0.0583 [-0.1374 - 0.2540]	0.5590468567	0.7380844829	-0.8583 [-1.3134 - -0.4032]	0.0002186330838	0.03703282838	0.0168 [-0.0044 - 0.0379]	0.1205951169	0.1752846544	-0.2736 [-0.5191 - -0.0281]	0.02893102832	0.03311325207	-0.0124 [-0.3191 - 0.2943]	0.9367129919	0.2867230223
	Posterior cingulate cortical surface area	0.0818 [-0.1317 - 0.2953]	0.4525566862	0.222858898	-0.3210 [-0.7390 - 0.0970]	0.132286993	0.1775049762	0.0022 [-0.0193 - 0.0236]	0.8441298866	0.3247632368	-0.1925 [-0.4484 - 0.0634]	0.1403895892	0.1410112251	-0.0993 [-0.3968 - 0.1982]	0.5129383364	0.2155701507
	Posterior cingulate cortical thickness	-0.1691 [-0.3654 - 0.0271]	0.09123789004	0.4731286609	-0.1873 [-0.6133 - 0.2388]	0.3890013271	0.7768294131	0.0036 [-0.0167 - 0.0239]	0.7292301429	0.7640166989	0.2089 [-0.0165 - 0.4342]	0.06925376988	0.4514535028	0.1477 [-0.1935 - 0.4889]	0.396082225	0.161165831
	Precentral cortical surface area	-0.0801 [-0.3170 - 0.1569]	0.5078761116	0.8970518133	0.2062 [-0.2256 - 0.6381]	0.3492851715	0.1634895925	0.0059 [-0.0164 - 0.0283]	0.6032206828	0.6350931161	0.0389 [-0.2068 - 0.2846]	0.7563120128	0.7204120769	-0.1819 [-0.5117 - 0.1478]	0.2794368488	0.9079045951
	Precentral cortical thickness	-0.0161 [-0.2181 - 0.1860]	0.8760995181	0.9363683349	-0.3357 [-0.7290 - 0.0576]	0.09433052735	0.3472756818	0.0025 [-0.0192 - 0.0243]	0.8188839455	0.8949933201	0.0155 [-0.2281 - 0.2591]	0.900789356	0.7393723571	0.0659 [-0.2564 - 0.3883]	0.6884317625	0.9606017457
	Precuneus cortical surface area	-0.1232 [-0.3365 - 0.0900]	0.2574097065	0.9760236209	-0.1502 [-0.5732 - 0.2727]	0.4862930669	0.6554662256	0.0144 [-0.0073 - 0.0360]	0.1937053779	0.9807424063	0.0159 [-0.2203 - 0.2521]	0.8948666837	0.3989400701	0.3432 [-0.0165 - 0.7029]	0.06145548308	0.5172731429
	Precuneus cortical thickness	0.1132 [-0.0890 - 0.3154]	0.2726082748	0.2391768874	0.1341 [-0.3465 - 0.6148]	0.5844324891	0.6600394741	0.0025 [-0.0190 - 0.0240]	0.8187796267	0.6748538744	0.0644 [-0.1665 - 0.2953]	0.584677893	0.1649897726	-0.2823 [-0.6468 - 0.0823]	0.1291105578	0.0506409533
	Rostral anterior cingulate cortical surface area	0.0725 [-0.2455 - 0.3905]	0.6548130137	0.8239993966	-0.2099 [-0.5758 - 0.1560]	0.2608077936	0.5539448934	-0.0058 [-0.0291 - 0.0176]	0.6292847157	0.4509088836	-0.2400 [-0.4899 - 0.0098]	0.05964616011	0.007423380374	-0.0171 [-0.3686 - 0.3344]	0.9239037007	0.2904358451
	Rostral anterior cingulate cortical thickness	-0.0552 [-0.2653 - 0.1549]	0.6068159567	0.2990252106	0.5339 [0.1433 - 0.9245]	0.007381691618	0.7268823945	-0.0007 [-0.0211 - 0.0197]	0.9467041938	0.3100470973	-0.0545 [-0.2929 - 0.1838]	0.6539089204	0.1922555265	-0.2107 [-0.5553 - 0.1338]	0.2306513678	0.2069493727
	Rostral middle frontal cortical surface area	-0.0205 [-0.2219 - 0.1809]	0.8417168073	0.07102156305	0.0134 [-0.3880 - 0.4147]	0.9479642367	0.7974080981	-0.0018 [-0.0227 - 0.0190]	0.8647864684	0.09997019447	-0.1140 [-0.3503 - 0.1224]	0.344560355	0.9198006698	0.1809 [-0.1485 - 0.5102]	0.2818301081	0.6479607888
	Rostral middle frontal cortical thickness	0.1123 [-0.0985 - 0.3232]	0.296492323	0.6736211993	-0.0012 [-0.4350 - 0.4327]	0.9958487305	0.1033949824	-0.0100 [-0.0311 - 0.0111]	0.35348256	0.5185812588	0.0710 [-0.1769 - 0.3190]	0.5744935084	0.1279812344	-0.2020 [-0.5185 - 0.1144]	0.2108498206	0.02909280401
	Superior frontal cortical surface area	-0.0275 [-0.2295 - 0.1745]	0.7894908251	0.01597507387	0.2808 [-0.1240 - 0.6856]	0.1740180011	0.2814873674	0.0085 [-0.0134 - 0.0304]	0.4457286493	0.02685263915	0.0506 [-0.1908 - 0.2921]	0.6810730824	0.01164847907	0.0984 [-0.2006 - 0.3974]	0.5188403586	0.056457749
	Superior frontal cortical thickness	-0.0560 [-0.2540 - 0.1421]	0.5796330789	0.7952087628	-0.2679 [-0.6991 - 0.1633]	0.2232914159	0.1524226893	-0.0095 [-0.0308 - 0.0118]	0.3825767568	0.8834196716	-0.0120 [-0.2700 - 0.2460]	0.9273011634	0.7153229637	-0.0381 [-0.3705 - 0.2942]	0.8220804685	0.1525796086
	Superior parietal cortical surface area	0.0621 [-0.1413 - 0.2655]	0.5493746725	0.8072434402	0.2183 [-0.2081 - 0.6447]	0.3156146048	0.09819820654	0.0046 [-0.0156 - 0.0249]	0.6530652875	0.5701334545	0.0669 [-0.1756 - 0.3093]	0.5887879684	0.5312137249	-0.1079 [-0.4022 - 0.1864]	0.4722510659	0.8801568036
	Superior parietal cortical thickness	-0.0803 [-0.2850 - 0.1243]	0.4417103772	0.9817017409	-0.3688 [-0.8101 - 0.0726]	0.1015191327	0.3344043907	0.0109 [-0.0101 - 0.0319]	0.308801036	0.791339994	-0.0757 [-0.3190 - 0.1675]	0.5417173845	0.3981332105	0.0717 [-0.2606 - 0.4039]	0.6724431152	0.5268781845
	Superior temporal cortical surface area	-0.0586 [-0.2911 - 0.1738]	0.621047126	0.8866429929	-0.1545 [-0.5403 - 0.2314]	0.4326341066	0.299804856	0.0104 [-0.0126 - 0.0333]	0.3767596927	0.443021274	-0.2483 [-0.4880 - -0.0087]	0.04222073643	0.009500302104	0.0624 [-0.2632 - 0.3879]	0.7071821325	0.694939586
	Superior temporal cortical thickness	0.0163 [-0.1790 - 0.2116]	0.8697578124	0.2860809531	-0.2502 [-0.7151 - 0.2148]	0.2915989496	0.2461618092	-0.0123 [-0.0334 - 0.0087]	0.2519437079	0.2048766523	0.0480 [-0.1981 - 0.2941]	0.7024832815	0.8239572058	0.2125 [-0.1199 - 0.5450]	0.2101920159	0.7924544514
	Supramarginal cortical surface area	-0.0052 [-0.2498 - 0.2394]	0.9666863674	0.6161060134	0.0250 [-0.3459 - 0.3959]	0.8950200136	0.05360892509	0.0253 [0.0028 - 0.0477]	0.02726909592	0.6195123757	-0.1981 [-0.4387 - 0.0425]	0.1065932303	0.533346318	-0.0605 [-0.3794 - 0.2585]	0.7101857806	0.7329669285
	Supramarginal cortical thickness	-0.0522 [-0.2565 - 0.1521]	0.6167993496	0.2395562552	-0.6051 [-1.0817 - -0.1285]	0.01283850037	0.5561793767	-0.0003 [-0.0216 - 0.0209]	0.9758331235	0.8124732904	0.1357 [-0.1214 - 0.3928]	0.3008000157	0.1133043338	-0.0230 [-0.3194 - 0.2733]	0.878843421	0.1395029078
	Temporal pole cortical surface area	0.1609 [-0.0659 - 0.3877]	0.1644286111	0.8950074644	-0.0945 [-0.5021 - 0.3131]	0.6495351876	0.2334386038	-0.0061 [-0.0290 - 0.0169]	0.6051485884	0.9417590128	-0.1551 [-0.4017 - 0.0916]	0.2178092912	0.2475146244	0.0644 [-0.2571 - 0.3860]	0.694546371	0.2250699023
	Temporal pole cortical thickness	0.1424 [-0.0657 - 0.3504]	0.1799094256	0.6538252201	-0.2626 [-0.6951 - 0.1699]	0.2340429144	0.0356494014	-0.0117 [-0.0332 - 0.0097]	0.2840153923	0.3544294041	-0.0551 [-0.3030 - 0.1928]	0.66295436	0.3226333238	-0.2005 [-0.5412 - 0.1402]	0.2486907533	0.7173762265
	Transverse temporal cortical surface area	-0.0651 [-0.2819 - 0.1517]	0.5563058333	0.3989233298	-0.3706 [-0.7772 - 0.0360]	0.07403815527	0.08069093281	0.0088 [-0.0140 - 0.0316]	0.4493855339	0.1580560032	-0.1696 [-0.4122 - 0.0731]	0.1708183182	0.02702079732	0.2914 [-0.0596 - 0.6424]	0.1037398894	0.8028696305
	Transverse temporal cortical thickness	-0.0182 [-0.2212 - 0.1849]	0.8608255991	0.4160986015	-0.2233 [-0.7533 - 0.3067]	0.4089370029	0.2865908981	0.0093 [-0.0130 - 0.0316]	0.4144077721	0.4796916289	-0.1278 [-0.3884 - 0.1328]	0.3365386393	0.1505165678	-0.0152 [-0.3263 - 0.2960]	0.9238907707	0.9705371236
Howard et al., 2019	Major depressive disorder	-0.2664 [-0.4624 - -0.0705]	0.007705632943	0.2210219968	0.1797 [-0.2594 - 0.6189]	0.422492401	0.007483411015	0.0017 [-0.0201 - 0.0234]	0.8800176004	0.5001971778	0.2513 [0.0215 - 0.4811]	0.0320831588	0.177192142	0.0539 [-0.2792 - 0.3869]	0.7512005876	0.3973940917
Kunkle et al., 2019	Alzheimer's disease	-0.1267 [-0.3901 - 0.1366]	0.3454970262	0.4555344525	0.2824 [-0.1488 - 0.7136]	0.1992125877	0.9211181945	0.0032 [-0.0191 - 0.0255]	0.7777753106	0.01572397115	-0.0578 [-0.2991 - 0.1834]	0.6383925601	0.8885021183	0.1077 [-0.3155 - 0.5310]	0.61789718	0.7636376518
Lee et al., 2018	Cognitive performance	0.3588 [0.1584 - 0.5593]	0.000450913998	0.8501963804	-0.2314 [-0.6649 - 0.2021]	0.2954154302	0.005140858713	0.0140 [-0.0078 - 0.0359]	0.2078486353	0.6828179273	0.1107 [-0.1411 - 0.3626]	0.3888582198	0.5146779853	0.0774 [-0.2118 - 0.3666]	0.5999596527	0.3082256193
	Educational attainment	0.3459 [0.1443 - 0.5476]	0.0007716186286	0.3509144398	-0.1652 [-0.5926 - 0.2621]	0.4484697139	0.4819955185	0.0029 [-0.0187 - 0.0245]	0.7904581028	0.9920026784	0.1453 [-0.0933 - 0.3838]	0.2326107608	0.797930793	0.0234 [-0.2598 - 0.3067]	0.8711135528	0.8255436247
Malik et al., 2018	Any ischemic stroke	-0.0991 [-0.2895 - 0.0912]	0.3074236555	0.8995429767	-0.0014 [-0.4660 - 0.4631]	0.9952214371	0.04805219676	0.0132 [-0.0066 - 0.0329]	0.1915569032	0.3293598	-0.2318 [-0.4686 - 0.0050]	0.05500524763	0.5124847888	-0.2857 [-0.6307 - 0.0593]	0.1045967038	0.9678832205
	Any stroke	-0.0836 [-0.2735 - 0.1062]	0.3877977502	0.9815461348	-0.0967 [-0.5470 - 0.3535]	0.6737067854	0.1007620608	0.0109 [-0.0092 - 0.0310]	0.2870411888	0.3242231229	-0.2034 [-0.4443 - 0.0376]	0.098094077	0.3930195838	-0.2864 [-0.6227 - 0.0499]	0.095136519	0.908093535
	Cardioembolic stroke	0.0147 [-0.1803 - 0.2097]	0.8827356193	0.5308808713	-0.2954 [-0.7231 - 0.1323]	0.1758201173	0.428749475	0.0003 [-0.0198 - 0.0204]	0.9745842082	0.195856407	-0.1169 [-0.3460 - 0.1122]	0.3172484889	0.008359183226	-0.0912 [-0.4102 - 0.2278]	0.5752312743	0.2736582263
	Large artery stroke	-0.1850 [-0.3851 - 0.0151]	0.06991423097	0.6802678466	0.1618 [-0.2508 - 0.5743]	0.4422059265	0.1081793444	0.0059 [-0.0158 - 0.0275]	0.5971048933	0.9371524369	0.0801 [-0.1717 - 0.3318]	0.5331668582	0.8503781198	-0.0051 [-0.3339 - 0.3237]	0.9759293444	0.5502700877
	Small vessel stroke	-0.2287 [-0.4281 - -0.0293]	0.02460104475	0.268318333	0.4190 [-0.0235 - 0.8616]	0.06348222456	0.1195099257	0.0065 [-0.0154 - 0.0284]	0.5596608604	0.1314328711	0.0336 [-0.2113 - 0.2785]	0.7882620886	0.7405334709	0.0134 [-0.3125 - 0.3392]	0.9359760502	0.8801684321
Nalls et al., 2019	Parkinson's disease or first degree relation to individual with Parkinson's disease	-0.0542 [-0.2591 - 0.1507]	0.6043223892	0.9922594112	-0.2852 [-0.7367 - 0.1662]	0.2156394803	0.01041855051	0.0065 [-0.0141 - 0.0270]	0.5372836875	0.6302756206	0.1098 [-0.1288 - 0.3484]	0.3669857007	0.7120516896	-0.0703 [-0.4211 - 0.2805]	0.6944024105	0.7402566734
Neale lab, 2018	Sleeplessness or insomnia	-0.0057 [-0.2069 - 0.1954]	0.9555539714	0.05409215991	-0.5532 [-0.9646 - -0.1417]	0.008418463902	0.2159295536	0.0154 [-0.0056 - 0.0364]	0.1495766057	0.795071053	0.1165 [-0.1125 - 0.3454]	0.3186886455	0.01225370913	-0.0019 [-0.3364 - 0.3326]	0.9912262109	0.05705454291
	Trouble falling asleep	-0.0600 [-0.2623 - 0.1422]	0.5605824329	0.260702515	-0.5934 [-1.0889 - -0.0979]	0.0189079197	0.03666923619	0.0054 [-0.0163 - 0.0271]	0.6259818885	0.4181472984	-0.0547 [-0.3013 - 0.1920]	0.6638879081	0.9428436385	0.1772 [-0.1530 - 0.5074]	0.2927774339	0.4645865632
Otowa et al., 2016	Anxiety disorder	-0.1126 [-0.3165 - 0.0912]	0.2787185505	0.5893274651	0.1316 [-0.3250 - 0.5881]	0.5721805451	0.2518520198	0.0066 [-0.0149 - 0.0281]	0.5461214384	0.8904346597	-0.1289 [-0.3510 - 0.0933]	0.2555513196	0.1523907244	0.0652 [-0.2648 - 0.3952]	0.6984952544	0.8893662096
Pulit et al., 2019	Body mass index	-0.0187 [-0.2200 - 0.1826]	0.8553729042	0.7686420202	-0.1689 [-0.6422 - 0.3044]	0.4843337683	0.5716362902	-0.0057 [-0.0273 - 0.0159]	0.6058296938	0.9068993521	0.0327 [-0.2152 - 0.2805]	0.7962512823	0.1062255739	-0.1372 [-0.4778 - 0.2033]	0.4296397756	0.9475112058
Sargurupremraj et al., 2020	White matter hyperintensities	-0.1030 [-0.3195 - 0.1136]	0.3513450729	0.2272374866	-0.1962 [-0.5993 - 0.2069]	0.3399882866	0.1501034065	0.0086 [-0.0130 - 0.0302]	0.4338684025	0.6332109644	-0.0748 [-0.3193 - 0.1697]	0.5487368705	0.3756296194	-0.0456 [-0.3889 - 0.2977]	0.794551606	0.4268062432
Satizabal et al., 2019	Accumbens subcortical volume	0.0748 [-0.1255 - 0.2751]	0.4643002888	0.2515422574	0.7847 [0.3430 - 1.2264]	0.0004971670091	0.2243859314	-0.0084 [-0.0297 - 0.0130]	0.4431590998	0.3626051293	0.1339 [-0.1033 - 0.3711]	0.268533908	0.2228265452	-0.2683 [-0.5822 - 0.0456]	0.09391834308	0.04835281512
	Amygdala subcortical volume	-0.0520 [-0.2596 - 0.1555]	0.6231198942	0.9587415492	0.1210 [-0.3336 - 0.5757]	0.6019197196	0.4845446941	-0.0128 [-0.0341 - 0.0085]	0.2385138964	0.2516950788	0.1561 [-0.0782 - 0.3905]	0.1916906239	0.05479793773	0.1701 [-0.1320 - 0.4722]	0.2697746437	0.2831445459
	Brainstem subcortical volume	0.1148 [-0.0821 - 0.3118]	0.2531660459	0.4081003226	0.1794 [-0.3078 - 0.6665]	0.4705114489	0.2202671929	-0.0016 [-0.0236 - 0.0205]	0.8885101407	0.9539780315	0.0599 [-0.1674 - 0.2873]	0.6054956844	0.5361165706	-0.1268 [-0.4358 - 0.1822]	0.4213018861	0.9977786466
	Caudate subcortical volume	-0.0397 [-0.2478 - 0.1683]	0.7081931882	0.6442999308	-0.0938 [-0.5504 - 0.3629]	0.6873678852	0.03844485121	0.0149 [-0.0067 - 0.0364]	0.1761762484	0.2345664284	-0.1079 [-0.3441 - 0.1284]	0.3708790046	0.6446808252	0.0504 [-0.2917 - 0.3925]	0.772685761	0.3601388441
	Pallidum subcortical volume	-0.0574 [-0.2585 - 0.1437]	0.5756606449	0.6202439395	0.0992 [-0.3117 - 0.5102]	0.6360625066	0.4078374547	0.0048 [-0.0171 - 0.0268]	0.6650216029	0.7536928922	0.0488 [-0.1769 - 0.2745]	0.6717870275	0.394001657	-0.0602 [-0.3825 - 0.2621]	0.7143107	0.3819693601
	Putamen subcortical volume	0.0709 [-0.1299 - 0.2717]	0.4888111082	0.4207416706	0.4813 [0.0746 - 0.8881]	0.02037838192	0.3113858896	0.0083 [-0.0135 - 0.0302]	0.4555462766	0.7835852553	-0.0690 [-0.2917 - 0.1538]	0.5438129041	0.08195222385	-0.0555 [-0.3671 - 0.2560]	0.7269437072	0.2120323355
	Thalamus subcortical volume	0.0410 [-0.1534 - 0.2353]	0.6795469431	0.945812107	0.3185 [-0.0748 - 0.7118]	0.1125144541	0.3729213098	-0.0061 [-0.0279 - 0.0156]	0.5795544995	0.9207968763	0.0811 [-0.1533 - 0.3154]	0.4978225153	0.9690578509	-0.1362 [-0.4665 - 0.1940]	0.4187441124	0.9940150501
Savage et al., 2018	Intelligence	0.5268 [0.2960 - 0.7577]	0.000007724659542	0.1777566008	0.0824 [-0.2947 - 0.4595]	0.668486689	0.04698359405	0.0111 [-0.0112 - 0.0334]	0.3275630691	0.8479048469	0.1245 [-0.1251 - 0.3741]	0.3281967981	0.443425403	0.0071 [-0.2710 - 0.2851]	0.960125876	0.02500181832
Schwartzentruber et al., 2021	Alzheimer's disease or family history of Alzheimer's disease	-0.2840 [-0.5479 - -0.0202]	0.03487334692	0.2634345833	0.2089 [-0.2054 - 0.6232]	0.3230476594	0.4019371784	0.0093 [-0.0131 - 0.0318]	0.4151536276	0.05443391089	0.0192 [-0.2201 - 0.2586]	0.8749641445	0.375980236	0.2349 [-0.1536 - 0.6235]	0.2359832413	0.9294637055
Yengo et al., 2018	Height	0.1603 [-0.0480 - 0.3687]	0.1315069784	0.6715605042	0.1866 [-0.2924 - 0.6656]	0.4451033115	0.01040576538	-0.0105 [-0.0328 - 0.0117]	0.3539543407	0.9770669245	-0.0937 [-0.3384 - 0.1511]	0.4531181255	0.3178198688	-0.1419 [-0.4761 - 0.1923]	0.4053847117	0.8399801806

Supplementary Table 7

		Meta-analysis (Iceberg. PPMI. PDBP. SURE-PD3. LCC)			DIGPD				Iceberg				PPMI				PDBP				SURE-PD3				LCC			
Study	Phenotype	Coefficient (95% CI)	p-value (coefficient)	Heterogeneity p-value	Coefficient	Lower bound (95% CI)	Upper bound (95% CI)	p-value	Coefficient	Lower bound (95% CI)	Upper bound (95% CI)	p-value	Coefficient	Lower bound (95% CI)	Upper bound (95% CI)	p-value	Coefficient	Lower bound (95% CI)	Upper bound (95% CI)	p-value	Coefficient	Lower bound (95% CI)	Upper bound (95% CI)	p-value	Coefficient	Lower bound (95% CI)	Upper bound (95% CI)	p-value
Adams et al., 2016	Intracranial volume	0.2149 [0.0737 - 0.3561]	0.002854888379	0.4067658561	-0.02583521534	-0.207895058	0.1562246273	0.780913463	0.03538516543	-0.3048362684	0.3756065993	0.838471843	0.3039011636	0.01433501714	0.5934673101	0.03968696592	0.3700020055	0.1020827029	0.6379213081	0.00679453776	0.1250218136	-0.1312211799	0.3812648071	0.3389343355	-0.4719263182	-1.97512706	1.031274424	0.538339412
Chia et al., 2021	Lewy body dementia	-0.1463 [-0.2619 - -0.0306]	0.01321723946	0.9242194371	-0.1967880037	-0.3548896284	-0.03868637888	0.0147054179	-0.1544808204	-0.4711420141	0.1621803734	0.3389944726	-0.2200314813	-0.4361725511	-0.003890411553	0.04601647288	-0.1417334307	-0.3622848985	0.07881803712	0.2078369495	-0.07398806726	-0.2864695123	0.1384933778	0.4949366438	-0.1967799763	-1.449759241	1.056199289	0.758225625
Davies et al., 2016	Memory performance	-0.0988 [-0.2144 - 0.0168]	0.09399882301	0.3693034514	-0.1625391346	-0.3228552414	-0.002223027836	0.04690667629	0.06434038501	-0.2325584479	0.3612392179	0.6710261545	-0.2822469537	-0.5016095149	-0.0628843925	0.01167479649	-0.02950862127	-0.2525392245	0.193521982	0.7953899443	-0.0719454738	-0.2853152268	0.1414242792	0.5086930751	-0.135353645	-1.436437357	1.165730067	0.8384332665
	Reasoning	0.2457 [0.1298 - 0.3615]	0.00003234898782	0.3390019851	0.1738439339	0.009435532314	0.3382523355	0.03822368866	0.1907888709	-0.1061326715	0.4877104133	0.2078903577	0.3832337466	0.1624802768	0.6039872165	0.0006676055646	0.3138973886	0.09664259544	0.5311521818	0.004628315717	0.06780616146	-0.1518237881	0.2874361111	0.5451139098	0.3018303326	-0.8678054811	1.471466146	0.613012193
Davies et al., 2018	Reaction time	-0.0889 [-0.2042 - 0.0264]	0.1308100059	0.02428025044	-0.07472226523	-0.236596543	0.08715201254	0.3656070251	-0.3313688395	-0.622328895	-0.04040878397	0.02560402823	-0.1425818077	-0.3624688872	0.07730527178	0.2037630636	-0.2052926226	-0.4243482033	0.01376295815	0.06623615234	0.1996938124	-0.01702421299	0.4164118378	0.07091839263	0.3353570892	-0.9494407286	1.620154907	0.6089390202
Grasby et al., 2020	Banks of the superior temporal sulcus cortical surface area	-0.0393 [-0.1770 - 0.0983]	0.5754826259	0.863129295	-0.09330663597	-0.2742954689	0.08768219692	0.3122864113	-0.01609532786	-0.3417160591	0.3095254033	0.9228213833	-0.09807884169	-0.3781794236	0.1820217402	0.4925285228	0.07434282499	-0.1829311604	0.3316168104	0.5711502295	-0.1168484411	-0.3747213375	0.1410244554	0.3744831732	-0.08737354867	-1.568719095	1.393971997	0.9079666398
	Banks of the superior temporal sulcus cortical thickness	0.0173 [-0.1011 - 0.1357]	0.7747439859	0.7778338348	-0.06449817413	-0.2259152736	0.09691892539	0.4335379526	-0.08294291348	-0.384328781	0.218442954	0.589616406	0.09376105395	-0.1344526114	0.3219747193	0.4206773629	0.08408188948	-0.1437005483	0.3118643273	0.4693811626	-0.05186069977	-0.2692312833	0.1655098838	0.6400620475	-0.2520338574	-1.446032009	0.9419642947	0.6790814063
	Caudal anterior cingulate cortical surface area	-0.0702 [-0.1981 - 0.0577]	0.2820479641	0.4028078193	0.1066475585	-0.0646415125	0.2779366296	0.2223482487	0.108406094	-0.2124673288	0.4292795169	0.507863705	-0.06243264326	-0.3197060305	0.194840744	0.634341347	-0.1204968767	-0.3599322418	0.1189384885	0.3239563394	-0.1519754096	-0.3851747711	0.08122395186	0.2014951657	1.005355015	-0.4197482249	2.430458255	0.1667634648
	Caudal anterior cingulate cortical thickness	-0.1101 [-0.2257 - 0.0055]	0.06196899147	0.5775219687	-0.1508276736	-0.3164294686	0.01477412143	0.07424407578	-0.1759504628	-0.4745104708	0.1226095452	0.2480633233	-0.1619868661	-0.3861706081	0.0621968758	0.1567177285	-0.1319369422	-0.3516841779	0.0878102935	0.2392878641	0.005741412503	-0.2049414635	0.2164242885	0.9574037654	-0.9781089208	-2.475139771	0.51892193	0.2003434609
	Caudal middle frontal cortical surface area	-0.0440 [-0.2207 - 0.1328]	0.6258782675	0.1354556869	0.08695475596	-0.1207859379	0.2946954499	0.4119943573	-0.3035556989	-0.6863211252	0.07920972739	0.1200967196	-0.0906341783	-0.4576667294	0.2763983728	0.6283936606	-0.1767452046	-0.5250534914	0.1715630823	0.3199492684	0.3114484388	-0.01678896683	0.6396858444	0.06292598966	-0.3983925449	-2.428804513	1.632019423	0.7005562035
	Caudal middle frontal cortical thickness	-0.0356 [-0.1545 - 0.0833]	0.5575815203	0.9095117706	-0.006665276701	-0.1722622141	0.1589316607	0.9371212706	-0.09497918705	-0.3966146671	0.206656293	0.5371326226	-0.04462358207	-0.2790821654	0.1898350012	0.7091244388	-0.08839944748	-0.3133711322	0.1365722373	0.4412167451	0.04698514178	-0.170456204	0.2644264875	0.6719211867	0.1498464354	-1.122634781	1.422327652	0.8174671579
	Cuneus cortical surface area	-0.0275 [-0.1501 - 0.0950]	0.6594211802	0.2816444411	0.0185178034	-0.1594380225	0.1964736293	0.83839197	-0.04240221831	-0.3555433689	0.2707389323	0.7907034039	-0.170184739	-0.4079367608	0.06756728281	0.1606295416	-0.02830680624	-0.2577232739	0.2011096614	0.8089100804	0.07589738112	-0.1532396653	0.3050344275	0.5162080879	1.015583242	-0.1888806753	2.22004716	0.09841069048
	Cuneus cortical thickness	0.0533 [-0.0727 - 0.1793]	0.4066802021	0.6651061726	0.1494044546	-0.02312470089	0.3219336101	0.08964695116	0.08511541704	-0.2581773549	0.428408189	0.627002556	-0.08499892358	-0.3310362819	0.1610384347	0.4983349442	0.09416254713	-0.1374616367	0.325786731	0.4255745897	0.09962311075	-0.1318731464	0.3311193679	0.398971527	0.6293938043	-0.583830251	1.84261786	0.3092552615
	Entorhinal cortical surface area	0.1677 [0.0399 - 0.2955]	0.01011182488	0.01997883717	-0.003614454763	-0.1724729421	0.1652440326	0.9665357831	0.4558610212	0.1551225932	0.7565994491	0.002969046988	0.3859651208	0.1382395933	0.6336906483	0.002260431884	-0.03036639149	-0.2739325588	0.2131997759	0.8069545459	-0.03025091878	-0.2772622516	0.216760414	0.810305176	-0.08216284767	-1.330046596	1.165720901	0.8973200988
	Entorhinal cortical thickness	0.0218 [-0.0934 - 0.1369]	0.7109284973	0.3079277275	0.05181757585	-0.1083954122	0.2120305639	0.5261396927	-0.01285569211	-0.3074406412	0.281729257	0.9318377718	-0.1056415695	-0.325382117	0.114098978	0.3460573448	0.1638202484	-0.0586274948	0.3862679917	0.1489069201	0.002888913156	-0.2089695104	0.2147473367	0.9786781343	0.8469490122	-0.3379223961	2.03182042	0.1612179301
	Frontal pole cortical surface area	-0.0102 [-0.1275 - 0.1072]	0.8652800123	0.4923643966	-0.007059388801	-0.1753007281	0.1611819505	0.93445596	0.01673153883	-0.2902424361	0.3237055137	0.9149259566	-0.02449580345	-0.2488696883	0.1998780814	0.830564848	-0.0939077083	-0.3128745818	0.1250591652	0.4005920926	0.09978219462	-0.1198367631	0.3194011524	0.3732003378	-0.9068508745	-2.203931447	0.3902296976	0.1705919391
	Frontal pole cortical thickness	0.0204 [-0.0936 - 0.1345]	0.7255271717	0.3092043777	0.07630044162	-0.09138405331	0.2439849365	0.372483885	-0.1758948464	-0.4724169179	0.1206272251	0.244976768	0.00363203907	-0.2178104327	0.2250745109	0.9743549856	-0.04643914461	-0.2664710413	0.1735927521	0.6791209397	0.1913879658	-0.01375394933	0.3965334809	0.06746594698	-0.1381304548	-1.364224842	1.087963932	0.8252423997
	Whole cortical surface area	0.2298 [0.0911 - 0.3685]	0.001167651168	0.005872223491	0.1515912236	-0.03389796589	0.3370804131	0.109203713	-0.03793534517	-0.3636325235	0.2877618332	0.8194243749	0.2476031181	-0.0354417278	0.530647964	0.08642863545	0.1559725287	-0.1092725326	0.42121759	0.2491069903	0.365826846	0.1098263252	0.6218273668	0.005097537056	2.375814965	1.077880065	3.673749866	0.0003336958109
	Whole cortical thickness	0.0293 [-0.0911 - 0.1498]	0.6329165397	0.8867134949	0.06476728973	-0.1071293233	0.2366639028	0.460225133	-0.1322933141	-0.4558490409	0.1912624127	0.4229134023	0.04267632678	-0.1914316158	0.2767842694	0.7208758772	0.05513741148	-0.1749183942	0.2851932172	0.6385379083	0.06373911364	-0.1513045408	0.2787827681	0.5612843196	0.1392667121	-1.115558216	1.39409164	0.8277980169
	Fusiform cortical surface area	0.0183 [-0.1036 - 0.1403]	0.7681365364	0.08349732982	-0.08942684015	-0.2512097372	0.07235605693	0.278636962	0.2000209027	-0.1002927858	0.5003345913	0.1917518321	0.1109033506	-0.1249367938	0.3467434949	0.3567012046	0.04292902708	-0.1923041726	0.2781622267	0.7205791871	-0.1534590379	-0.3795917667	0.07267369087	0.1834925444	-1.336771643	-2.669193385	-0.004349899754	0.04925674106
	Fusiform cortical thickness	-0.0445 [-0.1694 - 0.0804]	0.4847048499	0.9662524579	0.05495695171	-0.1129707317	0.2228846351	0.5212441511	-0.09229727286	-0.4177956744	0.2332011287	0.5783742214	0.0265585019	-0.2151598254	0.2682768292	0.8294955287	-0.06446469012	-0.3019170848	0.1729877046	0.5946565867	-0.07150494466	-0.2995450086	0.1565351193	0.5388371842	0.138813118	-1.173449601	1.451075837	0.8357537638
	Inferior parietal cortical surface area	-0.0139 [-0.1670 - 0.1393]	0.8590846924	0.9202450833	-0.09497151539	-0.2912204344	0.1012774036	0.3428784729	0.09788872961	-0.2569317189	0.4527091782	0.5887008406	-0.06782641256	-0.3727720369	0.2371192118	0.6628818559	-0.04764314265	-0.3384428577	0.2431565724	0.7481266487	0.007681681099	-0.2829282784	0.2982916406	0.958681962	-0.5806245039	-2.44365461	1.282405602	0.5413091323
	Inferior parietal cortical thickness	0.1004 [-0.0167 - 0.2176]	0.09286359346	0.5476639589	0.1037726774	-0.05983282763	0.2673781824	0.21380237	0.1182710804	-0.1778560458	0.4143982066	0.4337470593	0.08391226922	-0.1383972444	0.3062217828	0.4594201377	0.1312960068	-0.09578437984	0.3583763934	0.2571155748	0.04374905157	-0.1740380449	0.261536148	0.6937897069	1.063944471	-0.08645931814	2.214348261	0.06988402095
	Inferior temporal cortical surface area	0.0519 [-0.0651 - 0.1689]	0.3843098731	0.3425569687	-0.03011858635	-0.1977772408	0.1375400681	0.7247689389	0.3242094321	0.02069560861	0.6277232555	0.03629496092	-0.01329954678	-0.2413338835	0.2147347899	0.9089919594	-0.05314427858	-0.2734149017	0.1671263446	0.6363010628	0.08069546962	-0.1339497899	0.2953407291	0.4612168182	-0.1941585481	-1.435499344	1.047182248	0.7591793298
	Inferior temporal cortical thickness	-0.0305 [-0.1512 - 0.0901]	0.619641921	0.5779078226	0.06096620684	-0.1039932001	0.2259256138	0.468838895	-0.3138631853	-0.672647787	0.0449214165	0.08642448947	0.001440857291	-0.2207562232	0.2236379378	0.9898595067	0.0419377495	-0.1819832521	0.2658587511	0.7135608197	-0.02465153047	-0.2471303554	0.1978272944	0.8280745131	-0.05146260221	-1.386811182	1.283885978	0.939789473
	Insula cortical surface area	-0.0774 [-0.2544 - 0.0997]	0.3916979679	0.8758221848	0.1460386046	-0.05080369496	0.3428809041	0.1459157423	-0.1959579968	-0.5747083683	0.1827923747	0.3105604039	-0.05151940836	-0.4087204441	0.3056816274	0.7774165771	-0.008782425423	-0.3513803516	0.3338155008	0.9599285366	-0.09625306393	-0.4424204736	0.2499143457	0.5857708021	0.7828642161	-1.30568397	2.871412403	0.4625428366
	Insula cortical thickness	-0.0697 [-0.1835 - 0.0440]	0.2295943056	0.3236683723	-0.004114236076	-0.1676701492	0.1594416771	0.9606780366	-0.2848441746	-0.5759768807	0.006288531558	0.05515815601	0.08052647862	-0.1384929559	0.2995459131	0.4711455566	-0.01968862164	-0.2369321505	0.1975549072	0.8590134856	-0.134431452	-0.3437296803	0.07486677634	0.208074627	-0.3447972597	-1.592536666	0.9029421461	0.5880860576
	Isthmus cingulate cortical surface area	0.1021 [-0.0738 - 0.2780]	0.2553832337	0.5359965553	0.1299694153	-0.08776057839	0.3476994089	0.2420170855	0.1319391377	-0.2646256243	0.5285038997	0.5143430457	-0.05249095515	-0.4224781088	0.3174961985	0.7809626623	0.02694630127	-0.30915563	0.3630482326	0.8751375091	0.2348522244	-0.08937353904	0.5590779879	0.1556963091	1.323408562	-0.5669493211	3.213766445	0.1700202788
	Isthmus cingulate cortical thickness	-0.0537 [-0.1708 - 0.0634]	0.3687763528	0.008049135719	-0.1529325117	-0.3187817787	0.0129167553	0.0707128525	-0.2421452096	-0.5491492097	0.0648587906	0.1221299748	0.04533145022	-0.1770328225	0.2676957229	0.6894801558	-0.1737881735	-0.3966583793	0.04908203226	0.1264313605	0.1218709083	-0.0955472861	0.3392891028	0.2719286033	-1.645349665	-2.752074586	-0.5386247445	0.003570058667
	Lateral occipital cortical surface area	-0.0422 [-0.1684 - 0.0839]	0.5113871706	0.4471905543	0.04087225243	-0.1411374338	0.2228819387	0.6598420815	-0.1878676526	-0.506259736	0.1305244308	0.2474851885	0.01622323569	-0.236195119	0.2686415904	0.8997562442	0.09696437045	-0.13180489	0.3257336309	0.4061232451	-0.1730071206	-0.4119029525	0.0658887113	0.1557836745	0.1721356604	-1.024081949	1.36835327	0.7779138134
	Lateral occipital cortical thickness	-0.0682 [-0.1892 - 0.0528]	0.2694531997	0.2540695061	0.1120365538	-0.05465318829	0.2787262959	0.1877235864	-0.1393641304	-0.4466008698	0.1678726089	0.3739764655	-0.216710444	-0.4509684626	0.01754757457	0.069808861	0.1500655547	-0.08304357516	0.3831746845	0.2070424063	-0.08607718926	-0.3074920344	0.1353376559	0.4460867477	-0.3399192129	-1.536542189	0.8567037635	0.5776927834
	Lateral orbitofrontal cortical surface area	-0.0697 [-0.2173 - 0.0779]	0.3545323892	0.3398546923	0.01579549769	-0.1654983122	0.1970893076	0.8644086845	-0.1040491031	-0.4370031101	0.228904904	0.5402106426	0.1370510635	-0.1649972386	0.4390993656	0.3738354795	-0.2725022545	-0.5404695992	-0.004534909689	0.04624686623	0.01571178716	-0.2774987061	0.3089222804	0.9163556874	-0.3925639589	-1.995801296	1.210673378	0.6312906848
	Lateral orbitofrontal cortical thickness	-0.0614 [-0.1830 - 0.0602]	0.3222678947	0.5862891011	0.02677427574	-0.1383965012	0.1919450527	0.7507039668	-0.232625903	-0.5395819886	0.07433018255	0.137450124	-0.02190768991	-0.2663619719	0.2225465921	0.8605692768	-0.1297380194	-0.3613175378	0.1018414991	0.2721902832	0.04388932471	-0.1739387844	0.2617174338	0.6929127074	0.314242505	-1.019785481	1.648270491	0.644305465
	Lingual cortical surface area	-0.0251 [-0.2092 - 0.1590]	0.7889576588	0.6667330807	-0.07540341241	-0.2685439837	0.1177371589	0.4441623011	-0.03563471334	-0.4211358439	0.3498664172	0.856231057	-0.1917005745	-0.5802176152	0.1968164661	0.3335051206	-0.09577989925	-0.4472781074	0.2557183089	0.5932921107	0.1841329423	-0.1730038565	0.541269741	0.3122461455	0.4563578252	-1.684842318	2.597557968	0.6761440692
	Lingual cortical thickness	0.0814 [-0.0349 - 0.1977]	0.1700556678	0.04267696579	0.09607800262	-0.06711790126	0.2592739065	0.2485470199	0.01028526839	-0.3065115118	0.3270820486	0.9492623892	-0.1928489848	-0.4153666505	0.02966868093	0.08938732553	0.1804200979	-0.03353721909	0.3943774149	0.09838276414	0.2734549025	0.05677873183	0.4901310731	0.01337739043	0.07082835521	-1.152814136	1.294470847	0.9096745887
	Medial orbitofrontal cortical surface area	0.0397 [-0.0995 - 0.1789]	0.5763525379	0.8909214964	-0.01287136738	-0.1971992725	0.1714565377	0.8911401727	0.1224256678	-0.2029149893	0.4477663249	0.4607974814	0.08626728614	-0.1944858865	0.3670204588	0.5470144332	-0.05269043009	-0.3130575887	0.2076767286	0.6916348902	0.05192657408	-0.2131547889	0.3170079371	0.7010260598	-0.3493442056	-1.803132791	1.10444438	0.6376566318
	Medial orbitofrontal cortical thickness	-0.0090 [-0.1330 - 0.1150]	0.8867304763	0.08675116499	-0.01427675325	-0.186450391	0.1578968845	0.870895179	-0.3502328653	-0.6592845325	-0.04118119814	0.02634218553	0.08197419335	-0.1729575535	0.3369059402	0.5285419796	0.02750697879	-0.2100207259	0.2650346835	0.8204437157	0.09035045464	-0.1284223472	0.3091232565	0.4182616279	-0.9919615597	-2.327843907	0.3439207882	0.1455656478
	Middle temporal cortical surface area	-0.0680 [-0.1842 - 0.0483]	0.2517149014	0.1218204765	0.000638449069	-0.1629884818	0.1642653799	0.9938982362	0.1151063592	-0.1968120409	0.4270247594	0.4695079209	-0.3170786363	-0.5410585641	-0.09309870848	0.00552635707	0.02346504109	-0.1992602082	0.2461902904	0.8364078291	-0.02547645096	-0.2341197689	0.1831668669	0.8108559438	0.3241234853	-0.894155226	1.542402197	0.6020539434
	Middle temporal cortical thickness	-0.0654 [-0.1897 - 0.0588]	0.3021231886	0.1196935808	0.06100550239	-0.1081065738	0.2301175785	0.4795431447	0.3568262737	0.01931984219	0.6943327052	0.03825071072	-0.1877341453	-0.4303760626	0.05490771195	0.1294078661	-0.1103920316	-0.3352891574	0.1145050942	0.3360197396	-0.1086453312	-0.3392623366	0.1219716743	0.3558232032	0.1044804511	-1.426026678	1.63498758	0.8935627591
	Paracentral cortical surface area	0.0139 [-0.1299 - 0.1577]	0.84979105	0.2830704419	0.06077723817	-0.1198268459	0.2413813222	0.509529235	-0.06155493993	-0.3985271523	0.2754172724	0.7203224083	0.3007578762	0.01039266034	0.5911230921	0.04234491232	-0.10359708	-0.3785673746	0.1713732147	0.4602526797	-0.07156873154	-0.3381977137	0.1950602506	0.5988223019	0.06318454161	-1.574764615	1.701133698	0.939732251
	Paracentral cortical thickness	0.0235 [-0.0973 - 0.1442]	0.7033380296	0.7131416512	-0.05661408277	-0.2256734828	0.1124453172	0.5116011373	-0.03417546313	-0.3605325049	0.2921815787	0.8373818479	0.1074434707	-0.130071507	0.3449584484	0.375283968	0.01362250417	-0.2139907656	0.241235774	0.9066203023	-0.03311058889	-0.248728627	0.1825074493	0.7634338316	0.7326341778	-0.5099171166	1.975185472	0.2478302098
	Para hippocampal cortical surface area	-0.0508 [-0.1671 - 0.0655]	0.3918720794	0.2113483183	0.09454543014	-0.07373791474	0.262828775	0.2708302597	-0.01962731651	-0.320064459	0.280809826	0.8981150482	0.01566260049	-0.2076724454	0.2389976464	0.8906725254	0.0231020822	-0.2004136534	0.2466178178	0.8394653123	-0.1544028119	-0.367353243	0.05854761913	0.1552875632	-1.281418936	-2.468248263	-0.09458960901	0.03433018361
	Para hippocampal cortical thickness	0.1251 [-0.0044 - 0.2547]	0.05829443383	0.2169714261	-0.108043944	-0.2735318086	0.05744392065	0.2006774142	0.2704185446	-0.02933267834	0.5701697675	0.07703277318	0.2748716915	0.01756190111	0.5321814819	0.03628355802	0.004158707186	-0.255895702	0.2642131164	0.9749959033	-0.01671395973	-0.2526358676	0.2192079482	0.8895653254	0.8879319303	-0.4002540295	2.17611789	0.1767015569
	Pars opercularis cortical surface area	0.0355 [-0.1801 - 0.2511]	0.7470959595	0.4934542736	0.0270035618	-0.2017144334	0.255721557	0.8170018083	-0.1681149551	-0.6503603635	0.3141304533	0.494442225	0.1074313344	-0.3552415376	0.5701042064	0.649038663	0.1956838326	-0.186668604	0.5780362693	0.3158182485	-0.01615133165	-0.4405869842	0.4082843209	0.9405458555	-1.78644685	-4.384024155	0.8111304545	0.1776785315
	Pars opercularis cortical thickness	0.0274 [-0.0892 - 0.1440]	0.6451818168	0.3034101739	-0.02945552266	-0.1908790639	0.1319680186	0.7206115888	-0.1377108421	-0.4413202475	0.1658985633	0.3740039129	-0.0883831075	-0.3079625628	0.1311963478	0.4301665285	0.2105701794	-0.0128330544	0.4339734131	0.06469253839	0.0533039364	-0.1634517716	0.2700596444	0.6298139943	-0.01715376592	-1.244087603	1.209780071	0.9781388935
	Pars orbitalis cortical surface area	-0.0782 [-0.2054 - 0.0491]	0.2285141083	0.7189235901	0.1438008943	-0.02306006242	0.3106618511	0.09120057585	-0.08294029589	-0.3972538594	0.2313732676	0.6050231747	-0.1372151209	-0.3868105958	0.1123803541	0.2812612965	-0.0896260826	-0.3292852852	0.15003312	0.4635744664	0.01407385489	-0.2240020413	0.2521497511	0.9077609163	-0.8562588854	-2.198849166	0.4863313955	0.211299894
	Pars orbitalis cortical thickness	-0.0650 [-0.1934 - 0.0635]	0.3213979332	0.8183344308	-0.02265608915	-0.1929932319	0.1476810535	0.7943318996	-0.1603166526	-0.4972469312	0.176613626	0.3510362106	-0.02588418603	-0.2749436228	0.2231752507	0.8385921308	-0.1408838902	-0.3827667642	0.1009989839	0.2536308773	0.02694369895	-0.2082943592	0.2621817571	0.8223757565	-0.3671762953	-1.76467147	1.030318879	0.6065818721
	Pars triangularis cortical surface area	0.0066 [-0.1169 - 0.1300]	0.9170089856	0.7255791831	-0.1419157834	-0.3063621475	0.02253058071	0.09075434607	0.04571395636	-0.2608970538	0.3523249665	0.7701188232	0.08608782015	-0.1540002125	0.3261758528	0.4821929683	0.05757817575	-0.1827465151	0.2979026866	0.6386566553	-0.1289264573	-0.3535324685	0.09567955396	0.2605712054	0.0185471461	-1.362452384	1.399546676	0.9789998903
	Pars triangularis cortical thickness	0.1141 [-0.0041 - 0.2323]	0.05839396324	0.2688542707	-0.112915832	-0.2774246821	0.05159301818	0.1785338525	0.1288645818	-0.175831095	0.4335602585	0.407146708	-0.03840190182	-0.2659851278	0.1891813242	0.7408559275	0.1482101813	-0.07654999903	0.3729703615	0.1962085539	0.2400461788	0.02266788993	0.4574244676	0.03043779246	-0.8606145538	-2.187516832	0.4662877239	0.2036533909
	Pericalcarine cortical surface area	0.0461 [-0.0710 - 0.1632]	0.4405383184	0.3924176253	-0.000415719857	-0.1612556427	0.160424203	0.9959580289	0.02535404532	-0.2648602825	0.3155683731	0.8640439587	0.09208783172	-0.1348157683	0.3189914317	0.426355975	-0.07392556595	-0.2996341061	0.1517829742	0.5209118348	0.09169508892	-0.12551996	0.3089101379	0.4080225355	0.9827234305	-0.1410066289	2.10645349	0.08652395227
	Pericalcarine cortical thickness	0.1141 [-0.0050 - 0.2333]	0.06044242885	0.1603949547	0.2013064031	0.04156490357	0.3610479026	0.0135132451	0.3726604355	0.0660170566	0.6793038144	0.01722248066	0.04849246558	-0.1787111659	0.275696097	0.6757142532	0.1184328485	-0.1132453653	0.3501110623	0.3163799772	0.007593026354	-0.2101143116	0.2253003643	0.9455005817	1.093933719	-0.09744745394	2.285314891	0.0719157135
	Postcentral cortical surface area	0.0063 [-0.1545 - 0.1672]	0.9385739955	0.7991330161	0.1367274988	-0.07246796687	0.3459229644	0.2001910102	0.1709834362	-0.1921305389	0.5340974114	0.3560543124	-0.01528944593	-0.332799856	0.3022209641	0.9248069752	0.03093627424	-0.2832159586	0.3450885131	0.8469524673	-0.0969098278	-0.4027016517	0.2088819961	0.5345069992	-0.5502759193	-2.309681479	1.209129641	0.5398743196
	Postcentral cortical thickness	0.0577 [-0.0613 - 0.1767]	0.3420652425	0.7217768105	0.004400502044	-0.1600869328	0.1688879369	0.9581824407	0.2294127339	-0.09053484944	0.5493603172	0.1599150349	0.1075545424	-0.1213174826	0.3364265673	0.3570232947	-0.008274107017	-0.2326266094	0.2160783954	0.9423764102	-0.01030929233	-0.2275335336	0.2069149489	0.9258889789	0.2264163075	-0.9534466887	1.406279304	0.7068201114
	Posterior cingulate cortical surface area	0.0288 [-0.0995 - 0.1571]	0.6599567895	0.4749678667	0.06034051581	-0.118698964	0.2393799957	0.5088984015	0.0101554911	-0.3290215502	0.3493325324	0.9532034952	-0.001009749293	-0.2514037113	0.2493842128	0.9936937187	0.0745143648	-0.1674245789	0.3164533085	0.5460790346	-0.01925183248	-0.2526302608	0.2141265958	0.8715569304	1.139789779	-0.08648405378	2.366063612	0.06849508624
	Posterior cingulate cortical thickness	-0.1300 [-0.2447 - -0.0152]	0.02639388444	0.5308565102	-0.1623680948	-0.3229940406	-0.001742148956	0.04756641076	-0.256602516	-0.5598057052	0.04660067318	0.09717056791	-0.07384372134	-0.2927916327	0.14510419	0.5085933779	-0.1423216191	-0.3620425918	0.07739935371	0.2042474243	-0.08066142499	-0.2906998751	0.1293770252	0.451637231	-0.9287946308	-2.031189958	0.173600696	0.09867388472
	Precentral cortical surface area	0.0141 [-0.1455 - 0.1737]	0.8623084933	0.6083843453	-0.02701962137	-0.2181200715	0.1640808287	0.781689058	0.2697190348	-0.07818256851	0.6176206382	0.1286344411	-0.05161621621	-0.3741119254	0.2708794929	0.753751207	-0.08334450258	-0.3787942853	0.2121052802	0.5803364339	-0.02371463683	-0.3447959601	0.2973666864	0.8849001147	0.03160478591	-2.072686529	2.135896101	0.976515981
	Precentral cortical thickness	-0.0208 [-0.1426 - 0.1010]	0.7381216539	0.8745937444	-0.003320782938	-0.1708092019	0.164167636	0.9690019365	0.003685573499	-0.2916807569	0.2990519039	0.9804885767	0.01882696747	-0.2256624175	0.2633163524	0.880032886	-0.124356433	-0.3488322556	0.1001193895	0.2775708735	0.03368246576	-0.1961314033	0.2634963348	0.7739129554	0.1727475146	-1.370121792	1.715616822	0.8263014241
	Precuneus cortical surface area	-0.0049 [-0.1320 - 0.1221]	0.9392080741	0.9062743849	0.03121359045	-0.1419629537	0.2043901346	0.7238884782	0.04064700207	-0.2852206222	0.3665146263	0.8068625514	0.02328209596	-0.2193681165	0.2659323084	0.8508319733	-0.06933706262	-0.3144794069	0.1758052817	0.5793296049	-0.01337299912	-0.2472614257	0.2205154274	0.910772241	0.5092332222	-0.7646128588	1.783079303	0.4333241667
	Precuneus cortical thickness	0.1169 [-0.0061 - 0.2398]	0.06245916875	0.8856897067	-0.2093391958	-0.3776106012	-0.04106779036	0.01475625097	0.2297886395	-0.08349266838	0.5430699474	0.1505437302	0.1629185897	-0.08112100043	0.4069581798	0.1907193805	0.05537186231	-0.1774261554	0.28816988	0.6410833557	0.07125452946	-0.1521780631	0.2946871221	0.5319389197	0.3025706859	-0.9141014337	1.519242805	0.6259622823
	Rostral anterior cingulate cortical surface area	-0.0909 [-0.3272 - 0.1454]	0.4506592769	0.3330621278	0.04540307453	-0.1868766121	0.2776827612	0.7016390987	-0.1247735554	-0.6189815552	0.3694344443	0.6207162257	0.06496800703	-0.4314245151	0.5613605292	0.7975488039	-0.3596289311	-0.8016455833	0.08238772112	0.1107915888	0.03296633795	-0.4397664915	0.5056991674	0.8912842205	1.902578429	-0.5682732984	4.373430157	0.1312501853
	Rostral anterior cingulate cortical thickness	-0.0786 [-0.1990 - 0.0418]	0.2007641121	0.8902914209	-0.0834881421	-0.2514873061	0.0845110219	0.3300489557	-0.1948081951	-0.4966610495	0.1070446594	0.205903174	-0.08300070369	-0.3209279097	0.1549265023	0.4941445575	-0.03684393145	-0.2677504699	0.194062607	0.7544812704	-0.04021038228	-0.2587440122	0.1783232476	0.7183722344	-0.4258485575	-1.647970527	0.796273412	0.4946386257
	Rostral middle frontal cortical surface area	-0.0081 [-0.1226 - 0.1065]	0.8903643244	0.06367310143	-0.03744546575	-0.2013863231	0.1264953916	0.6543897878	0.1055066906	-0.1841312119	0.395144593	0.4752531165	-0.1360455889	-0.3534812847	0.08139010687	0.2200805061	-0.06987273959	-0.2937027464	0.1539572672	0.5406439126	0.05657530631	-0.1549040606	0.2680546732	0.6000471064	1.433796046	0.3101867948	2.557405296	0.01238301468
	Rostral middle frontal cortical thickness	0.0506 [-0.0674 - 0.1686]	0.400568319	0.3237738763	0.02658558339	-0.1379163954	0.1910875622	0.7514296888	0.0227176794	-0.2978069087	0.3432422675	0.8895169225	-0.00174761152	-0.2286150012	0.2251197781	0.9879539551	-0.01435731279	-0.2350233821	0.2063087566	0.8985271274	0.2005000346	-0.01521795836	0.4162180276	0.06850095505	-0.9251580483	-2.202809933	0.3524938361	0.1558332109
	Superior frontal cortical surface area	0.0598 [-0.0577 - 0.1773]	0.3185371804	0.8312105896	0.1412684459	-0.02398271509	0.306519607	0.09383291365	-0.02478260287	-0.3347918531	0.2852266474	0.8754951185	0.1409168171	-0.08395881776	0.3657924519	0.2193725136	0.04001152338	-0.1818847982	0.2619078449	0.7237785031	0.02987788453	-0.1868101368	0.2465659058	0.7869692073	0.5431287509	-0.6959034281	1.78216093	0.3902585243
	Superior frontal cortical thickness	-0.1244 [-0.2390 - -0.0098]	0.03337536254	0.6811380624	0.0001848381322	-0.1614713521	0.1618410284	0.9982119197	-0.04021389372	-0.3352643718	0.2548365843	0.7893665932	-0.2097071493	-0.430754086	0.01133978743	0.06296803607	-0.188254719	-0.4048130061	0.02830356809	0.08841806136	-0.01998596006	-0.2324648507	0.1924929306	0.8537341719	-0.3552624024	-1.517910412	0.8073856072	0.5492443777
	Superior parietal cortical surface area	0.0464 [-0.0722 - 0.1651]	0.4431859076	0.04144458278	0.001781931615	-0.1708834405	0.1744473037	0.9838622002	-0.3035755282	-0.6094303656	0.002279309199	0.05173192678	0.04615302694	-0.190414245	0.2827202988	0.7021805352	0.00676415406	-0.2149975959	0.228525904	0.9523287683	0.2718666665	0.05642125218	0.4873120808	0.01338930463	-0.4496399461	-1.725338298	0.826058406	0.4896785842
	Superior parietal cortical thickness	-0.0125 [-0.1358 - 0.1108]	0.8421609754	0.3486437826	-0.1618302392	-0.332103548	0.008443069583	0.06249345125	0.2689772165	-0.03178971601	0.569744149	0.07963555663	-0.04825643511	-0.2891416074	0.1926287372	0.6945862479	-0.0451832689	-0.2880073577	0.1976408199	0.7153364821	-0.1155523042	-0.3390463832	0.1079417749	0.3108916679	0.2333753356	-1.197253282	1.664003954	0.7491770621
	Superior temporal cortical surface area	-0.0925 [-0.2441 - 0.0590]	0.231311899	0.607002476	0.04084011988	-0.1323919933	0.214072233	0.6440319443	-0.2319637912	-0.5629217962	0.09899421378	0.1695313976	-0.134305447	-0.4341640259	0.1655531319	0.3800197224	0.066629091	-0.2080892809	0.3413474629	0.63452912	-0.105024455	-0.4263365336	0.2162876236	0.5217590738	-0.7788030482	-2.433155987	0.8755498911	0.3561779127
	Superior temporal cortical thickness	0.0022 [-0.1153 - 0.1197]	0.9709614637	0.4408669681	-0.0745949374	-0.2354974285	0.08630755367	0.3635370634	0.0992582731	-0.21915044	0.4176669862	0.5412101493	0.1547391598	-0.07002076052	0.3794990801	0.1772197423	-0.126008915	-0.3489681559	0.09695032593	0.2679894929	-0.06236574561	-0.2757517721	0.1510202809	0.5667575902	0.0291588147	-1.165296895	1.223614524	0.9618387765
	Supramarginal cortical surface area	0.0461 [-0.1078 - 0.1999]	0.5572552603	0.4884386624	0.04567545848	-0.1480482277	0.2393991446	0.6440002147	-0.1336487441	-0.4857563172	0.218458829	0.4569130892	0.02413601936	-0.2896569421	0.3379289808	0.8801693101	0.2494361106	-0.03643324413	0.5353054653	0.08723405426	-0.03517456663	-0.3300309303	0.2596817971	0.8151311236	0.4339567556	-1.379380037	2.247293548	0.6390362664
	Supramarginal cortical thickness	-0.0559 [-0.1796 - 0.0678]	0.3757743065	0.1481803058	0.06114331739	-0.1098932612	0.232179896	0.4835144552	-0.09236191236	-0.4111532366	0.2264294119	0.5701360409	0.1504525638	-0.09412063237	0.3950257599	0.2279336522	-0.246240684	-0.4803726525	-0.01210871548	0.03927172213	-0.05919680779	-0.28284963	0.1644560144	0.6039235209	0.7911906115	-0.5866625647	2.169043788	0.2603983178
	Temporal pole cortical surface area	0.1406 [-0.0061 - 0.2872]	0.06031796925	0.6965324258	0.1723229791	-0.01258958071	0.357235539	0.0677716915	0.2668035563	-0.08982098642	0.6234280991	0.1425613876	0.2333606936	-0.0552867371	0.5220081244	0.1130668259	-0.02521539466	-0.3104337599	0.2600029706	0.8624351271	0.1400810534	-0.1277499288	0.4079120357	0.3053155128	-0.01817080924	-1.625618375	1.589276756	0.9823237524
	Temporal pole cortical thickness	0.0031 [-0.1166 - 0.1229]	0.9590258328	0.7797471366	0.0249123198	-0.1409267834	0.190751423	0.7684328359	0.07265788579	-0.2332315678	0.3785473393	0.6415376296	-0.08420524148	-0.3144786451	0.1460681622	0.4735530825	0.107897949	-0.1221410739	0.3379369719	0.3579359041	-0.04857812721	-0.2686589872	0.1715027327	0.6652908634	-0.008852706902	-1.259888307	1.242182893	0.9889342553
	Transverse temporal cortical surface area	-0.1156 [-0.2577 - 0.0265]	0.1107541257	0.1859206237	0.1001410276	-0.06979758736	0.2700796425	0.2481055908	-0.4666704253	-0.7935088381	-0.1398320125	0.00513406044	-0.110263313	-0.3814485245	0.1609218985	0.4254992004	-0.01885876256	-0.2912240294	0.2535065043	0.8920511079	0.04054846637	-0.2397023414	0.3207992741	0.7767322394	-0.3486978735	-2.054567522	1.357171775	0.6886870658
	Transverse temporal cortical thickness	0.0062 [-0.1149 - 0.1273]	0.9204495453	0.278078041	-0.1005778781	-0.2731724372	0.07201668094	0.2533913305	0.1906601029	-0.1488235324	0.5301437382	0.2710050636	0.1022456879	-0.1233023618	0.3277937375	0.3742756135	-0.1135066139	-0.3430532365	0.1160400087	0.3324621696	-0.07579277623	-0.2970788703	0.1454933179	0.5020243784	0.9706840962	-0.4841754593	2.425543652	0.190978342
Howard et al., 2019	Major depressive disorder	-0.1130 [-0.2294 - 0.0033]	0.05695330395	0.4637404329	0.04829584002	-0.1234284037	0.2200200837	0.581481796	0.07201274431	-0.2193783793	0.3634038679	0.6281202207	-0.2517338001	-0.4765382041	-0.02692939609	0.02818162653	-0.160877951	-0.3837698087	0.06201390671	0.1571697943	-0.04401955307	-0.2607327562	0.1726936501	0.690545548	-0.04434224349	-1.113895018	1.025210531	0.9352371606
Kunkle et al., 2019	Alzheimer's disease	-0.1537 [-0.2685 - -0.0390]	0.008637965208	0.607553232	-0.1854459888	-0.343119397	-0.02777258062	0.02115582983	-0.3489503182	-0.6453432882	-0.05255734814	0.02102634647	-0.1896389571	-0.4057945426	0.0265166285	0.08551832071	-0.09643332962	-0.3160352556	0.1231685764	0.3894170822	-0.07964940993	-0.2936971932	0.1343983733	0.4658040966	0.1475164043	-1.124879453	1.419912262	0.8202447002
Lee et al., 2018	Cognitive performance	0.4290 [0.3120 - 0.5459]	6.46323E-13	0.3448856367	0.1409219956	-0.02603075492	0.3078747462	0.09805185437	0.1461847064	-0.1620608521	0.4544302649	0.3526258023	0.5086183857	0.2868889617	0.7303478098	0.00000692757032	0.5088122609	0.2899113365	0.7277131853	0.000005220373328	0.404926316	0.1852030192	0.6246496128	0.000303842178	0.6990954432	-0.5013135694	1.899504456	0.2536844323
	Educational attainment	0.3646 [0.2440 - 0.4852]	0.0000000000309893807	0.2104839349	0.1544630862	-0.02006135213	0.3289875246	0.08279949792	0.2499418687	-0.05107133567	0.5509550731	0.1036464095	0.4949158156	0.2655992206	0.7242324106	0.00002336551768	0.4405078752	0.2113005201	0.6697152302	0.0001653495892	0.1996714798	-0.02911804794	0.4284610076	0.08716988251	1.225427778	-0.09348965449	2.544345211	0.06860159388
Malik et al., 2018	Any ischemic stroke	-0.1222 [-0.2393 - -0.0052]	0.04072093586	0.7542699567	-0.04775186775	-0.2202168407	0.1247131052	0.5873555395	-0.03685629714	-0.3629133594	0.2892007651	0.8246664231	-0.01908423933	-0.243392597	0.2052241183	0.867563243	-0.187394074	-0.4058504232	0.03106227513	0.0927091226	-0.1934989588	-0.4061030388	0.01910512111	0.07445018124	0.02294513034	-1.247133256	1.293023517	0.9717539702
	Any stroke	-0.1470 [-0.2647 - -0.0292]	0.01445566812	0.7950332834	-0.05568339044	-0.2270837278	0.115716947	0.5242942561	-0.2252532987	-0.5453108122	0.09480421482	0.1677704785	-0.02484952207	-0.2491765711	0.199477527	0.8281208052	-0.1743603433	-0.3944313297	0.04571064314	0.1204559754	-0.2008989866	-0.4182518217	0.01645384857	0.07004917598	-0.07726309277	-1.326180848	1.171654662	0.9034920064
	Cardioembolic stroke	-0.0360 [-0.1555 - 0.0835]	0.5545043331	0.7097329909	0.1365043477	-0.07498504604	0.3479937415	0.2058551883	0.1440533162	-0.2389262974	0.5270329297	0.4609900322	0.03361261002	-0.19356042	0.2607856401	0.7718185062	-0.1262410955	-0.3434067125	0.09092452147	0.2545565203	-0.05721121247	-0.2705417816	0.1561193567	0.5991486674	-0.3371857843	-1.566679483	0.892307914	0.59091154
	Large artery stroke	-0.1045 [-0.2289 - 0.0198]	0.09938194781	0.4608709981	-0.08618880776	-0.3207259315	0.148348316	0.4713668956	0.07124354209	-0.3242255454	0.4667126296	0.72402363	-0.1168231346	-0.3610557744	0.1274095052	0.3484992418	-0.1222113582	-0.3473241943	0.1029014778	0.2873089725	-0.09918696396	-0.3166196214	0.1182456935	0.3712773995	-1.17931351	-2.434050769	0.07542374798	0.06545347212
	Small vessel stroke	-0.1223 [-0.2461 - 0.0014]	0.05266066666	0.7302294968	-0.02237851801	-0.2468201481	0.2020631121	0.8450616985	0.06862500841	-0.3689906581	0.5062406749	0.7585745993	-0.1750156148	-0.4004134883	0.05038225867	0.1280438367	-0.1813032776	-0.4083679047	0.0457613494	0.1175914256	-0.08071872972	-0.3022331881	0.1407957287	0.475103023	0.4171633058	-0.8520446407	1.686371252	0.5194450889
Nalls et al., 2019	rkinson's disease or first degree relation to individual with Parkinson's dise	-0.0315 [-0.1527 - 0.0897]	0.6108393878	0.9205356394	0.01546130714	-0.1604626654	0.1913852797	0.8632378591	-0.08337744019	-0.3928368789	0.2260819985	0.5974495542	-0.01254759846	-0.2498781544	0.2247829575	0.9174687457	0.04180509747	-0.1904559195	0.2740661144	0.724255174	-0.07796820078	-0.2973156957	0.1413792941	0.4860034981	-0.3682749821	-1.714763293	0.9782133285	0.5919133998
Neale lab, 2018	Sleeplessness or insomnia	0.0615 [-0.0548 - 0.1777]	0.3000522108	0.07942979214	0.1479789291	-0.01618077442	0.3121386326	0.0772658035	-0.1240677999	-0.4343999762	0.1862643764	0.4332897484	0.304134063	0.08643944091	0.521828685	0.006177615276	0.07500723086	-0.145358004	0.2953724657	0.5046913831	-0.09124398316	-0.3083224027	0.1258344364	0.4100382364	-0.2656489666	-1.451601396	0.9203034627	0.6606436406
	Trouble falling asleep	-0.0466 [-0.1625 - 0.0692]	0.4304157878	0.06451041312	-0.02464796098	-0.186526483	0.1372305611	0.7653763693	-0.07777225566	-0.3886740965	0.2331295852	0.623931532	0.08915927529	-0.1295569785	0.307875529	0.4243045117	-0.3022705514	-0.5220737753	-0.08246732745	0.007032166672	0.06002949906	-0.1534589288	0.273517927	0.5815581145	0.6649378533	-0.6120681298	1.941943836	0.3074655402
Otowa et al., 2016	Anxiety disorder	-0.1031 [-0.2185 - 0.0122]	0.07975407144	0.2685884621	0.09808993962	-0.06375138359	0.2599312628	0.2348693013	-0.2025245542	-0.4959909777	0.09094186934	0.1761854699	-0.1293470789	-0.3465119638	0.08781780597	0.2430538348	-0.04133570183	-0.2649175998	0.1822461961	0.7170851977	-0.1264949226	-0.3423223494	0.08933250412	0.2506710531	1.059106331	-0.03277549629	2.150988159	0.05728487111
Pulit et al., 2019	Body mass index	-0.0733 [-0.1928 - 0.0461]	0.2286869831	0.4090688919	0.03865490753	-0.1511097753	0.2284195835	0.6897141414	0.04230619366	-0.2884030611	0.3730154484	0.8020231349	-0.07328414978	-0.311889053	0.1653207534	0.5471904785	-0.1981583922	-0.4214878245	0.02517104006	0.08202418638	-0.0379663335	-0.2481318865	0.1721992195	0.723288817	0.8168350307	-0.3597404572	1.993410519	0.1736082526
Sargurupremraj et al., 202	White matter hyperintensities	-0.0934 [-0.2236 - 0.0369]	0.159993827	0.02328842674	0.009538354097	-0.1577582976	0.1768350058	0.9110243364	-0.02417533616	-0.3479793941	0.2996287218	0.8836595668	-0.4246082673	-0.6772083125	-0.172008222	0.000985607547	-0.04983773075	-0.2982904681	0.1986150066	0.694205832	0.1483954015	-0.09402806857	0.3908188716	0.2302331081	-0.628819037	-1.994128619	0.7364905454	0.3666860287
Satizabal et al., 2019	Accumbens subcortical volume	0.0798 [-0.0362 - 0.1957]	0.1774478326	0.2690791481	-0.04513686853	-0.2064759726	0.1162022355	0.5834671794	0.04916525675	-0.2527779174	0.3511084309	0.749620383	0.2564396255	0.03552305993	0.477356191	0.02289853307	0.01514509417	-0.2062029359	0.2364931242	0.8933196833	-0.03174357786	-0.2455848387	0.182097683	0.7710925841	0.8015696339	-0.4324953321	2.0356346	0.2029942018
	Amygdala subcortical volume	-0.0598 [-0.1758 - 0.0562]	0.3123885907	0.5544132945	0.00325883572	-0.1591184355	0.1656361069	0.968622857	0.02654743925	-0.2630454665	0.316140345	0.857409295	-0.06720860958	-0.2911162096	0.1566989905	0.5563261108	0.03633663937	-0.187323645	0.2599969237	0.7501643458	-0.1705654202	-0.384386048	0.04325520765	0.1179421487	-0.6999489899	-1.998130355	0.5982323756	0.2906180848
	Brainstem subcortical volume	0.1469 [0.0332 - 0.2605]	0.01130194186	0.3418120982	0.06617352109	-0.09608443357	0.2284314758	0.4240988479	0.2600079068	-0.03733421412	0.5573500277	0.08655236152	-0.0352980142	-0.2566303099	0.1860342815	0.7546039373	0.2556944607	0.04192285715	0.4694665643	0.01906139864	0.1595567682	-0.04812394559	0.367237482	0.1321186466	-0.228106864	-1.49236038	1.036146652	0.7236142517
	Caudate subcortical volume	-0.0453 [-0.1665 - 0.0760]	0.4645732418	0.1328340594	-0.1492690943	-0.3120790996	0.01354091106	0.07234296328	-0.3976423357	-0.6989105873	-0.09637408418	0.00968308046	0.02792612346	-0.2056583806	0.2615106275	0.8147343163	0.09264837496	-0.1347067833	0.3200035333	0.4244671592	-0.05385373178	-0.2853112376	0.177603774	0.6483690009	0.008642984499	-1.250232783	1.267518752	0.9892636687
	Pallidum subcortical volume	-0.0266 [-0.1439 - 0.0907]	0.6567844694	0.8817343761	0.004983029641	-0.1608262621	0.1707923214	0.9530298781	-0.007429673592	-0.3104723586	0.2956130114	0.9616745754	0.03876594658	-0.1847488145	0.2622807077	0.733907605	-0.08632551214	-0.3129975459	0.1403465216	0.4554074665	-0.05612425347	-0.2710928139	0.1588443069	0.6088541381	0.4268519926	-0.8366632437	1.690367229	0.5078862936
	Putamen subcortical volume	0.0739 [-0.0472 - 0.1951]	0.2317332432	0.4110824963	-0.07585909801	-0.2469393979	0.09522120186	0.3848077453	-0.09903561432	-0.4161513311	0.2180801024	0.5404722088	0.004337505359	-0.2325318408	0.2412068515	0.9713696877	0.1243856841	-0.1022111626	0.3509825308	0.2819800479	0.1464034276	-0.07520089613	0.3680077512	0.1953702877	1.0527239	-0.343845908	2.449293708	0.1395667782
	Thalamus subcortical volume	0.0610 [-0.0539 - 0.1760]	0.2980091385	0.3924262215	0.1015421251	-0.0610402685	0.2641245187	0.2209103298	0.3034738794	0.01087924255	0.5960685163	0.0420687622	-0.06872689187	-0.2889923408	0.1515385571	0.5408388402	0.04932628081	-0.1728927671	0.2715453287	0.6635217677	0.07020330407	-0.1401917638	0.2805983719	0.5131192368	-0.1703841074	-1.519007756	1.178239541	0.8044281329
Savage et al., 2018	Intelligence	0.5592 [0.4082 - 0.7102]	3.86173E-13	0.0864013204	0.195655232	0.01455750923	0.3767529547	0.03421613381	0.2076607696	-0.1448328025	0.5601543418	0.2482327058	0.6801864141	0.3866678916	0.9737049365	0.00000557412885	0.7068508583	0.4309351459	0.9827665707	0.000000513746163	0.4702811264	0.1653683089	0.7751939438	0.002503293807	1.881559863	0.1518259836	3.611293743	0.03300699221
chwartzentruber et al., 20	Alzheimer's disease or family history of Alzheimer's disease	-0.1683 [-0.2833 - -0.0533]	0.00412151882	0.6699979892	-0.2192044461	-0.3760047354	-0.06240415685	0.006143915213	-0.2854235893	-0.5930070919	0.02215991323	0.06894835893	-0.2409381373	-0.4541051626	-0.0277711119	0.02673910651	-0.1291381356	-0.349115686	0.09083941474	0.2498969726	-0.09023234697	-0.3041591398	0.1236944458	0.4084101142	0.4151583581	-0.9298294345	1.760146151	0.5451899564
Yengo et al., 2018	Height	0.1109 [-0.0205 - 0.2422]	0.09804329872	0.07778046864	-0.06598113832	-0.252566825	0.1206045484	0.4882531363	0.4546823215	0.101543564	0.807821079	0.01161787248	0.05171451542	-0.2136025878	0.3170316186	0.7024405249	-0.1353369424	-0.3871425708	0.1164686859	0.2921513655	0.2020042494	-0.02466713726	0.4286756361	0.08069387536	0.4901118618	-0.9132089218	1.893432645	0.49364666
