# Supplementary material for: Proxy-analysis of the genetics of cognitive decline in Parkinson’s disease through polygenic scores
Source: NPJ Parkinsons Dis. 2024 Jan 4;10:8. doi: 10.1038/s41531-023-00619-5 (PMC10767119; doi:10.1038/s41531-023-00619-5)
Supplement: Supplementary file 2 — Reporting summary [file 41531_2023_619_MOESM2_ESM.pdf]

nature portfolio

Corresponding author(s): Jean-Christophe Corvol

Last updated by author(s): Jun 23, 2023

Reporting Summary

Nature Portfolio wishes to improve the reproducibility of the work that we publish. This form provides structure for consistency and transparency in reporting. For further information on Nature Portfolio policies, see our Editorial Policies and the Editorial Policy Checklist.

Statistics

For all statistical analyses, confirm that the following items are present in the figure legend, table legend, main text, or Methods section.

n/a	Confirmed	
☐	☒	The exact sample size (n) for each experimental group/condition, given as a discrete number and unit of measurement
☐	☒	A statement on whether measurements were taken from distinct samples or whether the same sample was measured repeatedly
☐	☒	The statistical test(s) used AND whether they are one- or two-sided<br>*Only common tests should be described solely by name; describe more complex techniques in the Methods section.*
☐	☒	A description of all covariates tested
☐	☒	A description of any assumptions or corrections, such as tests of normality and adjustment for multiple comparisons
☐	☒	A full description of the statistical parameters including central tendency (e.g. means) or other basic estimates (e.g. regression coefficient) AND variation (e.g. standard deviation) or associated estimates of uncertainty (e.g. confidence intervals)
☐	☒	For null hypothesis testing, the test statistic (e.g. F, t, r) with confidence intervals, effect sizes, degrees of freedom and P value noted<br>*Give P values as exact values whenever suitable.*
☒	☐	For Bayesian analysis, information on the choice of priors and Markov chain Monte Carlo settings
☒	☐	For hierarchical and complex designs, identification of the appropriate level for tests and full reporting of outcomes
☒	☐	Estimates of effect sizes (e.g. Cohen's d, Pearson's r), indicating how they were calculated

Our web collection on statistics for biologists contains articles on many of the points above.

Software and code

Policy information about availability of computer code

Data collection	*Provide a description of all commercial, open source and custom code used to collect the data in this study, specifying the version used OR state that no software was used.*
Data analysis	pandas, scipy, statsmodels, bigsnpr

For manuscripts utilizing custom algorithms or software that are central to the research but not yet described in published literature, software must be made available to editors and reviewers. We strongly encourage code deposition in a community repository (e.g. GitHub). See the Nature Portfolio guidelines for submitting code & software for further information.

Data

Policy information about availability of data

All manuscripts must include a data availability statement. This statement should provide the following information, where applicable:

- Accession codes, unique identifiers, or web links for publicly available datasets
- A description of any restrictions on data availability
- For clinical datasets or third party data, please ensure that the statement adheres to our policy

The datasets generated and analyzed during the current study are available from the corresponding author upon reasonable request. The genotype and clinical data for the Parkinson's progression markers initiative (PPMI) included in this study are publicly available upon request to ppmi@loni.usc.edu through a PPMI Whole Genome Sequencing Data Agreement. Clinical data for the Parkinson's disease biomarker program (PDBP) included in this study are publicly available through

https://pdbp.ninds.nih.gov. Clinical longitudinal data and genotyping data for the other cohorts included are accessible through appropriate data sharing agreements that protect patient privacy with the institutions that conducted or are conducting study consents and clinical assessments under local institutional review board approvals.

Research involving human participants, their data, or biological material

Policy information about studies with human participants or human data. See also policy information about sex, gender (identity/presentation), and sexual orientation and race, ethnicity and racism.

Reporting on sex and gender	Only sex, and not gender, was reported in the studies used for this work. Sex was used as a covariate in the different analyses of this work.
Reporting on race, ethnicity, or other socially relevant groupings	Years of education, as self-reported by the subject, was used as a covariate in the different analyses of this work.<br>Race or ethnicity were not used in this study.
Population characteristics	Population characteristics are described in the supplementary information:<br>AMP-PD cohorts key inclusion criteria<br><br>Further details of inclusion and exclusion criteria can be found at https://amp-pd.org/unified-cohorts. As several of these studies included multiple study arms, only the inclusion criteria for the idioapthic PD study arm are summarised here as these are the patients we included for our analysis.<br><br>PPMI: PD subjects must have 2 of the following symptoms: resting tremor, bradykinesia, rigidity, OR either asymmetric resting tremor or asymmetric bradykinesia. PD participants were required be 30 years or older at time of PD diagnosis, have a diagnosis of PD for 2 years or less at screening, Hoehn and Yahr stage I or II at baseline, confirmation of dopamine transporter deficit by DaTSCAN, and not expected to requrie PD medication for at least 6 months from baseline visit.<br><br>SURE-PD3: PD subjects had to fulfill diagnostic criteria for idiopathic PD with at least 2 of the cardinal signs of PD (resting tremor, bradykinesia, and rigidity), Hoehn and Yahr stage 1 to 2.5 (inclusive), absence of current or imminent PD disability requiring dopaminergic therapy (within 90 days of enrollment), aged 30 years or older at time of PD diagnosis, a diagnosis of PD within 3 years prior to the screening visit, and non-fasting serum urate ≤ 5.7 mg/dL at the first screening visit.<br><br>LCC: Idiopathic PD participants were eligible for this study if they were of Ashkenazi Jewish (AJ) descent and have PD or parkinsonism, aged 18 years or older, and no history of neurological or psychological illness.<br><br>PDBP: Clinically diagnosed with PD and aged 21 years or older.<br><br>DIGPD: Subjects were recruited in this longitudinal multicenter study at 4 University Hospitals and 4 General hospital in France between 2009 and 2013 and followed annually for up to 7 years. Inclusion criteria were patients with a diagnosis of Parkinson's disease according to the UK Parkinson's disease Society Brain Bank criteria with a disease duration of less than 6 years at baseline. Exclusion criteria were atypical parkinsonism or history of treatment with neuroleptics. Patients for whom the diagnosis was revised to atypical parkinsonism during the follow-up of the study were excluded from the analysis. A complete description of the population is available elsewhere (Corvol et al., Neurology 2018, PMID: 29925549).<br><br>Iceberg: This is an ongoing monocenter longitudinal clinical study conducted at the Pitié-Salpêtrière Hospital, Paris, France. Inclusion criteria for PD patients were a diagnosis of Parkinson's disease according to UK Parkinson's Disease Society Brain Bank criteria with a disease duration of less than 4 years at baseline. Exclusion criteria were atypical parkinsonism such as multiple system atrophy, supranuclear palsy, dementia with Lewy bodies, or history of treatment with neuroleptics.
Recruitment	See above.
Ethics oversight	All studies were conducted according to good clinical practice, obtained approval from local ethic committees and regulatory authorities, and all patients provided informed consent before inclusion.

Note that full information on the approval of the study protocol must also be provided in the manuscript.

Field-specific reporting

Please select the one below that is the best fit for your research. If you are not sure, read the appropriate sections before making your selection.

☒ Life sciences ☐ Behavioural & social sciences ☐ Ecological, evolutionary & environmental sciences

For a reference copy of the document with all sections, see nature.com/documents/nr-reporting-summary-flat.pdf

Life sciences study design

All studies must disclose on these points even when the disclosure is negative.

Sample size	*Describe how sample size was determined, detailing any statistical methods used to predetermine sample size OR if no sample-size calculation was performed, describe how sample sizes were chosen and provide a rationale for why these sample sizes are sufficient.*
Data exclusions	*Describe any data exclusions. If no data were excluded from the analyses, state so OR if data were excluded, describe the exclusions and the rationale behind them, indicating whether exclusion criteria were pre-established.*
Replication	*Describe the measures taken to verify the reproducibility of the experimental findings. If all attempts at replication were successful, confirm this OR if there are any findings that were not replicated or cannot be reproduced, note this and describe why.*
Randomization	*Describe how samples/organisms/participants were allocated into experimental groups. If allocation was not random, describe how covariates were controlled OR if this is not relevant to your study, explain why.*
Blinding	*Describe whether the investigators were blinded to group allocation during data collection and/or analysis. If blinding was not possible, describe why OR explain why blinding was not relevant to your study.*

Reporting for specific materials, systems and methods

We require information from authors about some types of materials, experimental systems and methods used in many studies. Here, indicate whether each material, system or method listed is relevant to your study. If you are not sure if a list item applies to your research, read the appropriate section before selecting a response.

Materials & experimental systems

n/a	Involved in the study	
☒	☐	Antibodies
☒	☐	Eukaryotic cell lines
☒	☐	Palaeontology and archaeology
☒	☐	Animals and other organisms
☐	☒	Clinical data
☒	☐	Dual use research of concern
☒	☐	Plants

Methods

n/a	Involved in the study	
☒	☐	ChIP-seq
☒	☐	Flow cytometry
☒	☐	MRI-based neuroimaging

Clinical data

Policy information about clinical studies

All manuscripts should comply with the ICMJE guidelines for publication of clinical research and a completed CONSORT checklist must be included with all submissions.

Clinical trial registration	PPMI: NCT01141023<br>SURE-PD3: NCT02642393<br>LCC: https://www.michaeljfox.org/news/lrrk2-cohort-consortium<br>PDBP: NCT01767818<br>DIGPD: NCT01564992<br>ICEBERG: NCT02305147
Study protocol	For AMP-PD cohorts (PPMI, SURE-P3, LLC and PDBP) all information available at: https://amp-pd.org/unified-cohorts<br>DIGPD: available on request to the corresponding author (JC Corvol)<br>ICEBERG: available on request to the corresponding author (JC Corvol)
Data collection	For AMP-PD cohorts (PPMI, SURE-P3, LLC and PDBP) all information available at: https://amp-pd.org/unified-cohorts<br>DIGPD: Recruitment between 2009 and 2016. Database stored at the Assistance Publique Hôpitaux de Paris.<br>ICEBERG: Recruitment since 2014, ongoing. Database stored at the Paris Brain Institute.
Outcomes	MOCA (or MMSE when MOCA was not available) were pre-defined as primary outcome for this study.
